# Deaths from COPD in patients with cancer: a population-based study

**DOI:** 10.18632/aging.202939

**Published:** 2021-04-27

**Authors:** Yongqiang Zheng, Yan Huang, Xiwen Zheng, Jiangtong Peng, Ying Chen, Kaixu Yu, Yun Yang, Xi Wang, Xue Yang, Jiaxin Qian, Xindi Wang, Xiaolan Gao, Bian Wu

**Affiliations:** 1 Cancer Center, Union Hospital, Tongji Medical College, Huazhong University of Science and Technology, Wuhan 430022, China; 2 State Key Laboratory of Oncology in South China, Sun Yat-sen University Cancer Center, Sun Yat-sen University, Guangzhou 510060, China; 3 Department of Emergency Surgery, Union Hospital, Tongji Medical College, Huazhong University of Science and Technology, Wuhan 430022, China; 4 Department of Clinical Medicine, Fujian Medical University, Fuzhou 350000, China; 5 Department of Obstetrics and Gynecology, Tongji Hospital, Tongji Medical College, Huazhong University of Science and Technology, Wuhan 430022, China; 6 Department of Orthopedics, Tongji Hospital, Tongji Medical College, Huazhong University of Science and Technology, Wuhan 430022, China

**Keywords:** cancer survivors, chronic obstructive pulmonary disease, mortality, surveillance, epidemiology, end results program

## Abstract

Features of the deaths caused by COPD (chronic obstructive pulmonary disease) in cancer patients remained a controversial issue. This study aimed to characterize the demographic characteristics and mortality rates of the deaths from COPD in patients with cancer. In total, 7,846,370 cancer patients aged 40 years or older in the United States were identified from the Surveillance, Epidemiology, and End Results database (1975–2016). Mortality rates and SMRs (standardized mortality ratios) adjusted by age, race, sex, and calendar year were calculated to investigate the risk of COPD deaths in cancer survivors and to compare it with the general population. A total of 119,228 COPD deaths in patients with cancer were recorded, with a mortality rate of 261.5/100,000 person-years, nearly two-fold that of the general population (SMR, 2.17; 95% CI [confidence interval], 2.16–2.18). The proportion of cancer survivors dying from COPD increased from 0.9% in 1975 to 3.4% in 2016. Patients with lung cancer had a higher overall risk (SMR, 9.23; 95% CI, 9.12–9.35) than those with extrapulmonary malignancies. Among all extrapulmonary sites, laryngeal (SMR, 5.54; 95% CI, 5.34–5.75) and esophageal cancers (SMR, 4.33; 95% CI, 4.04–4.63) had the highest SMR. The risk of death from COPD increased with follow-up time.

## INTRODUCTION

Cancer and COPD (chronic obstructive pulmonary disease) are both leading causes of death in the USA and worldwide [1, 2]. In the USA, 595,930 deaths were due to cancer, and 155,041 deaths were due to COPD in 2015 [1]. Worldwide, 8.8 million deaths were due to cancer [3], and 3.2 million deaths were due to COPD in 2015 [4].

COPD and lung cancer have been proved to be closely related diseases. COPD is an independent risk factor and sometimes could be the driving factor for lung cancer, particularly for squamous cell cancer [5]. The high prevalence of lung cancer in COPD was interpreted as the common mechanism between these two closely related diseases, such as premature aging in the lungs, genetic predispositions to either disease or common pathogenic factors, such as growth factors, activation of intracellular pathways, or epigenetics [5]. The relationship between COPD and extrapulmonary cancers has not been well investigated, but the epidemiological studies showed that the risks of developing extrapulmonary cancers were increasing than those without [6]. In contrast, an increased risk of COPD has also been found in patients with cancer [7].

Similar risk factors are shared between cancer and COPD, such as age and smoking. As aging populations increase, older adults increase in number and make up a growing proportion of the population in nearly all countries [8]. Most of the new cancer cases were diagnosed among the elderly, and it is predicted that the number of incident cancers in the elderly will double in 2035 compared with 2012 [9]. Given the progressive decline in pulmonary function with increasing age and structural/morphological alveolar changes in the elderly [10, 11], COPD is also prevalent in the elderly population [12]. The aging of the population will increase the number of patients with cancer and COPD. It has been well known for many years that smoking causes COPD [13]. Tobacco smoke is a complex mixture with many types of carcinogens [14], associated with at least 17 types of human cancer, including lung, laryngeal, and pharyngeal cancers [15]. With shared risk factors, cancer and COPD might occur in the same individual, leading to a challenging, complicated condition for both clinic physicians and researchers. Besides, as the aging population increases, this particular population with cancer and COPD will rise. As the survival rates of patients with cancer continue to increase, the estimated number of cancer survivors in 2030 is expected to reach 22.1 million in the USA, and the majority of these are elderly patients [16]. The burden of COPD is likely to increase among cancer survivors, and respiratory care provision is of particular importance in this patient group [17].

Given that COPD is a significant cause of death in the aging population, the population with cancer will not be an exception. However, the studies describing COPD's characteristics and mortality in patients with cancer are lacking. Another aspect of the optimal management of cancer survivors is cancer-specific follow-up care [18], which requires a multidisciplinary care team, including specialists (such as oncologists or pulmonologists) and PCPs (primary care physicians) [19]. In this team, PCPs are mainly responsible for primary prevention, while pulmonologists provide COPD management. Therefore, identifying and targeting sub-populations of cancer survivors with the highest risk of COPD mortality is crucial. While few achievements have been made in the overlapping fields of COPD and cancer care, there is currently no contemporary resource that can assist specialists and policymakers in creating cancer survivorship programs to mitigate COPD's mortality risk.

In this study, we conducted a comprehensive analysis of the landscape of COPD mortality in cancer survivors. We aimed to characterize the characteristics and incidence of COPD mortality in patients with cancer. Our work provides a contemporary resource for oncologists, pulmonologists, and PCPs, as we highlight both cancer types and basic clinical characteristics, which together may influence patient-level decisions regarding respiratory care.

## RESULTS

### Objective 1: COPD deaths by demographic characteristics

**Figure 1:**
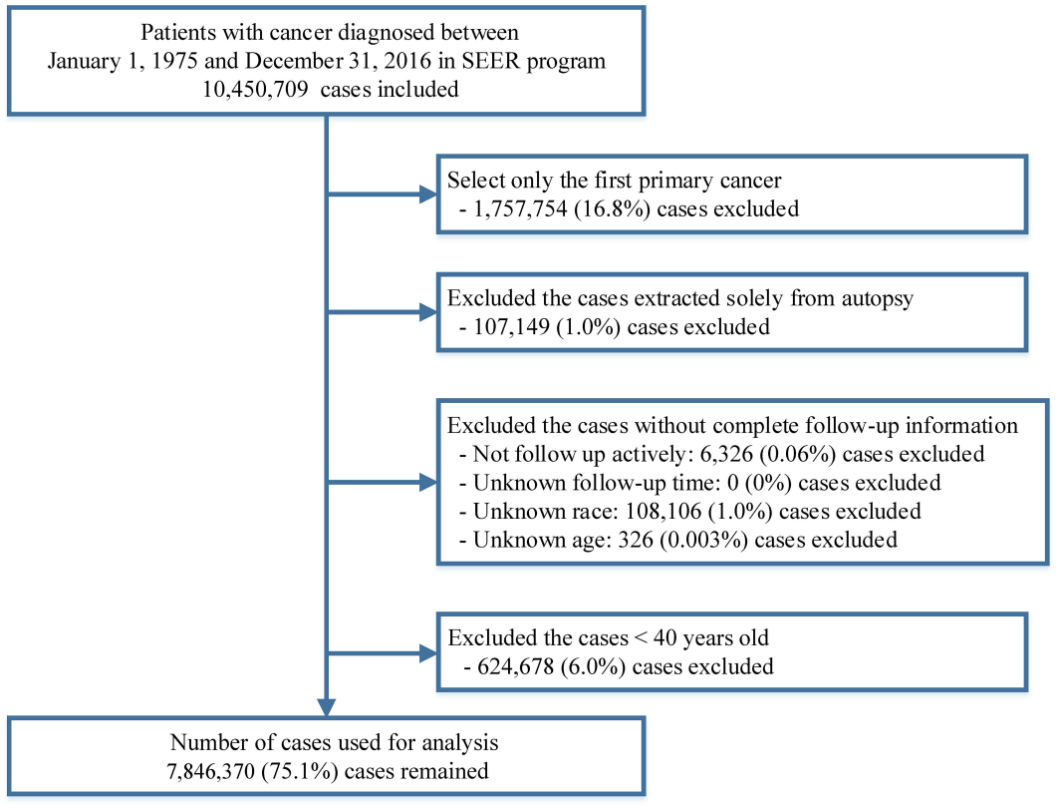
Flow chart of inclusion and exclusion criteria for cases included in this study.

**Figure 2:**
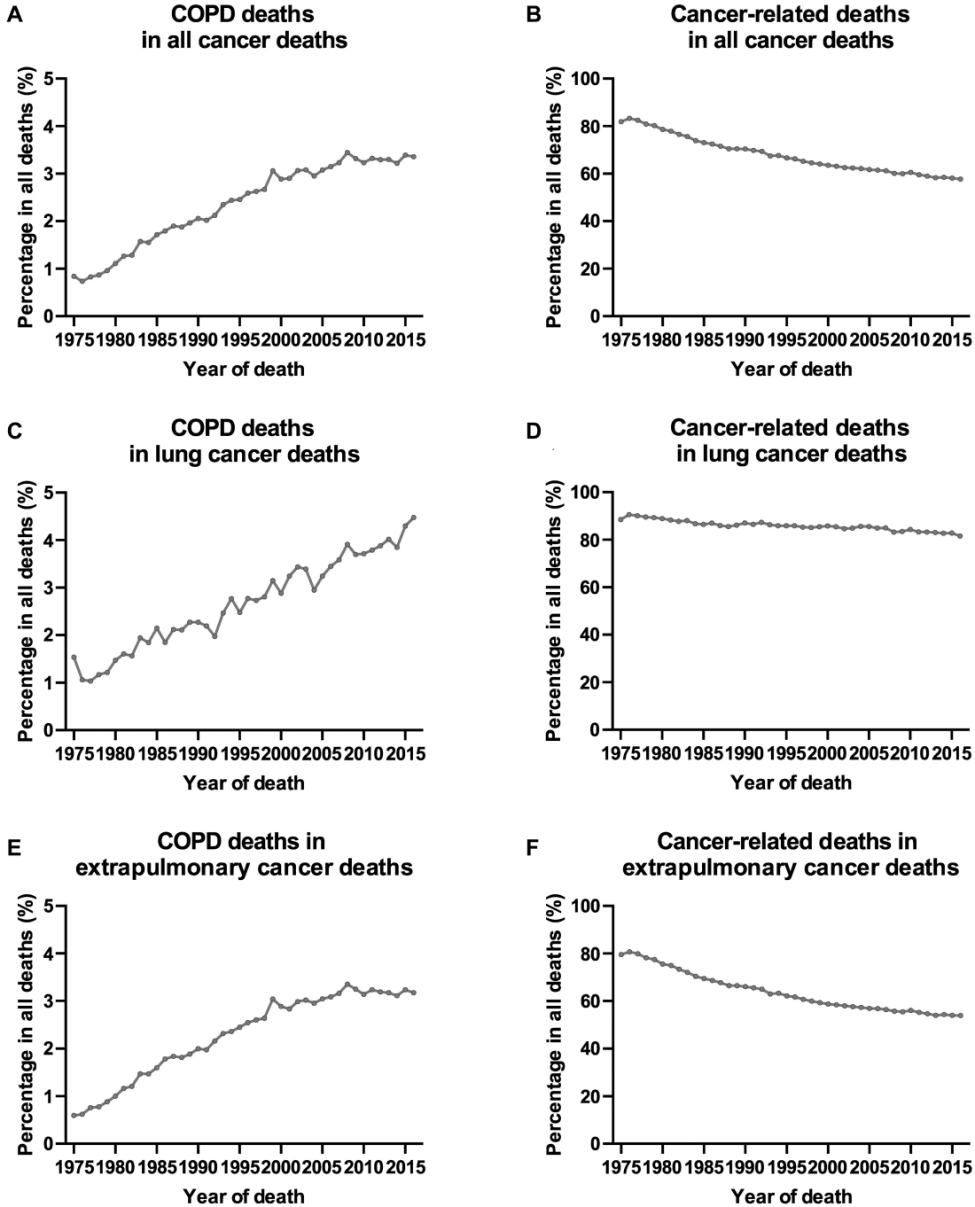
**Trends of COPD deaths and cancer-related deaths among all cancer deaths in SEER 9 registries by calendar year of death.** (**A**) Trends of COPD deaths among all cancer deaths in SEER 9 registries by calendar year of death; (**B**) trends of cancer-related deaths among all cancer deaths in SEER 9 registries by calendar year of death; (**C**) trends of COPD deaths among lung cancer deaths in SEER 9 registries by calendar year of death; (**D**) trends of cancer-related deaths among lung cancer deaths in SEER 9 registries by calendar year of death; (**E**) trends of COPD deaths among extrapulmonary cancer deaths in SEER 9 registries by calendar year of death; (**F**) trends of cancer-related deaths among extrapulmonary cancer deaths in SEER 9 registries by calendar year of death.

A total of 7,846,370 patients diagnosed with a first primary cancer between 1975 and 2016 were included in this study, with a median follow-up time of 3.5 years (range: 0–41.9 years) (Figure 1). COPD was the second leading cause of death among all non-cancer causes (Supplementary Tables 1 and 2), accounting for 119,228 deaths during the follow-up period (Table 1). The COPD mortality rate in patients with cancer was 261.5 per 100,000 person-years. The corresponding age-, sex-, race-, and calendar-year-adjusted COPD mortality rate in the general population was 120.5 per 100,000 person-years. This yielded an SMR (standardized mortality rate) of 2.17 (95% CI [confidence interval], 2.16–2.18). As expected, the proportion of cancer-related deaths for all patients with cancer declined over time (Figure 2). In contrast, the percentage of patients with cancer dying from COPD increased steadily over time. The proportion of cancer survivors dying from COPD increased from 0.9% in 1975 to 3.4% in 2016. This increase was more pronounced in patients with lung cancer in recent years (2005–2015) (Figure 2).

**Table 1:** COPD mortality among patients diagnosed with cancer between 1975 and 2016 in SEER 18 registries by baseline characteristics.

**Characteristics**		**No. of patients with cancer (%)**	**Person-years of follow-up**	**Death from COPD**				SMR^*^ (95% CI)
				**Patients with cancer**		**General population**		
				**No. of observed COPD deaths (%)**	**Mortality**	**No. of Expected COPD deaths (%)**	**Mortality**	
All		7,846,370 (100%)	45,601,152	119,228 (100%)	261.5	54,955	120.5	2.17 (2.16–2.18)
Age							
	40–59	2,519,598 (32.1%)	19,189,793	14,459 (12.1%)	75.3	2,795	14.6	5.17 (5.09–5.26)
	60–79	4,165,541 (53.1%)	23,176,048	79,553 (66.7%)	343.3	35,821	154.6	2.22 (2.21–2.24)
	80+	1,161,231 (14.8%)	3,235,312	25,216 (21.1%)	779.4	16,339	505.0	1.54 (1.52–1.56)
Sex							
	Female	3,899,940 (49.7%)	24,323,346	51,969 (43.6%)	213.7	21,463	88.2	2.420 (2.40–2.44)
	Male	3,946,430 (50.3%)	21,277,807	67,259 (56.4%)	316.1	33,492	157.4	2.01 (1.99–2.02)
Race							
	White	6,531,177 (83.2%)	38,835,087	108,069 (90.6%)	278.3	50,481	130.0	2.14 (2.13–2.15)
	Black	795,400 (10.1%)	3,889,420	7,426 (6.2%)	190.9	3,085	79.3	2.41 (2.35–2.46)
	Other	519,793 (6.6%)	2,876,646	3,733 (3.1%)	129.8	1,389	48.3	2.69 (2.60–2.78)
Year							
	1975–1989	1,015,383 (12.9%)	8,110,919	23,883 (20.0%)	294.5	7,218	89.0	3.31 (3.27–3.35)
	1990–1999	1,213,899 (15.5%)	10,580,651	29,878 (25.1%)	282.4	12,979	122.7	2.30 (2.28–2.33)
	2000–2009	3,181,105 (40.5%)	21,014,768	51,214 (43.0%)	243.7	26,925	128.1	1.90 (1.89–1.92)
	2010–2016	2,435,983 (31.0%)	5,894,814	14,253 (12.0%)	241.8	7,833	132.9	1.82 (1.79–1.85)
Marital status							
	Married	4,428,425 (56.4%)	28,888,368	60,504 (50.7%)	209.4	32,318	111.9	1.87 (1.86–1.89)
	Unmarried	2,862,961 (36.5%)	13,449,450	50,655 (42.5%)	376.6	18,097	134.6	2.80 (2.77–2.82)
	Unknown	554,984 (7.1%)	3,263,335	8,069 (6.8%)	247.3	4,540	139.1	1.78 (1.74–1.82)
Stage							
	*In situ*	447,307 (5.7%)	4,136,939	5,942 (5.0%)	143.6	3,836	92.7	1.55 (1.51–1.59)
	Localized	2,938,939 (37.5%)	24,566,825	56,065 (47.0%)	228.2	29,528	120.2	1.90 (1.88–1.91)
	Regional	1,291,329 (16.5%)	7,318,490	19,633 (16.5%)	268.3	7,400	101.1	2.65 (2.62–2.69)
	Distant	1,376,743 (17.5%)	2,725,893	10,987 (9.2%)	403.1	3,475	127.5	3.16 (3.10–3.22)
	Unstaged	1,792,052 (22.8%)	6,853,006	26,601 (22.3%)	388.2	10,716	156.4	2.48 (2.45–2.51)
Surgery							
	Yes	4,627,898 (59.0%)	35,043,784	71,451 (59.9%)	203.9	36,740	104.8	1.94 (1.93–1.96)
	No	3,096,678 (39.5%)	10,147,592	46,310 (38.8%)	456.4	17,664	174.1	2.62 (2.60–2.65)
	Unknown	121,794 (1.6%)	409,777	1,467 (1.2%)	358.0	551	134.5	2.66 (2.53–2.80)
Chemotherapy							
	Yes	1,913,324 (24.4%)	8,070,822	13,749 (11.5%)	170.4	6,442	79.8	2.13 (2.10–2.17)
	No/Unknown	5,933,046 (75.6%)	37,530,330	105,479 (88.5%)	281.1	48,513	129.3	2.17 (2.16–2.19)
Radiotherapy							
	Yes	2,147,710 (27.4%)	12,695,889	27,279 (22.9%)	214.9	13,735	108.2	1.99 (1.96–2.01)
	No/unknown	5,698,660 (72.6%)	32,905,263	91,949 (77.1%)	279.4	41,220	125.3	2.23 (2.22–2.25)
Smoking prevalence							
	Low	1,308,905 (16.7%)	8,406,893	17,316 (14.5%)	206.0	10,599	126.1	1.63 (1.61–1.66)
	Median	1,308,906 (16.7%)	9,119,074	21,469 (18.0%)	235.4	11,513	126.3	1.86 (1.84–1.89)
	High	1,308,906 (16.7%)	8,433,315	24,940 (20.9%)	295.7	10,969	130.1	2.27 (2.25–2.30)
	Unknown	3,919,653 (50.0%)	19,641,871	55,503 (46.6%)	282.6	21,874	111.4	2.54 (2.52–2.56)
SES							
	Low	2,276,448 (29.0%)	10,011,552	31,310 (26.3%)	312.7	13,472	134.6	2.32 (2.30–2.35)
	Median	2,276,449 (29.0%)	13,393,773	34,245 (28.7%)	255.7	17,115	127.8	2.00 (1.98–2.02)
	High	2,276,450 (29.0%)	14,074,860	29,766 (25.0%)	211.5	17,136	121.7	1.74 (1.72–1.76)
	Unknown	1,017,023 (13.0%)	8,120,968	23,907 (20.1%)	294.4	7,232	89.1	3.31 (3.26–3.35)

Abbreviations: COPD, chronic obstructive pulmonary disease; SMR, standardized mortality ratios; CI, confidence interval; SES, socioeconomic status.

^*^The SMRs were estimated as the ratios of observed to expected number of deaths. The observed values represented the number of COPD deaths in cancer patients, whereas the expected values represented the number of individuals who died of COPD in the general population, with a similar distribution of age, sex, race, and calendar year.

**Figure 3:**
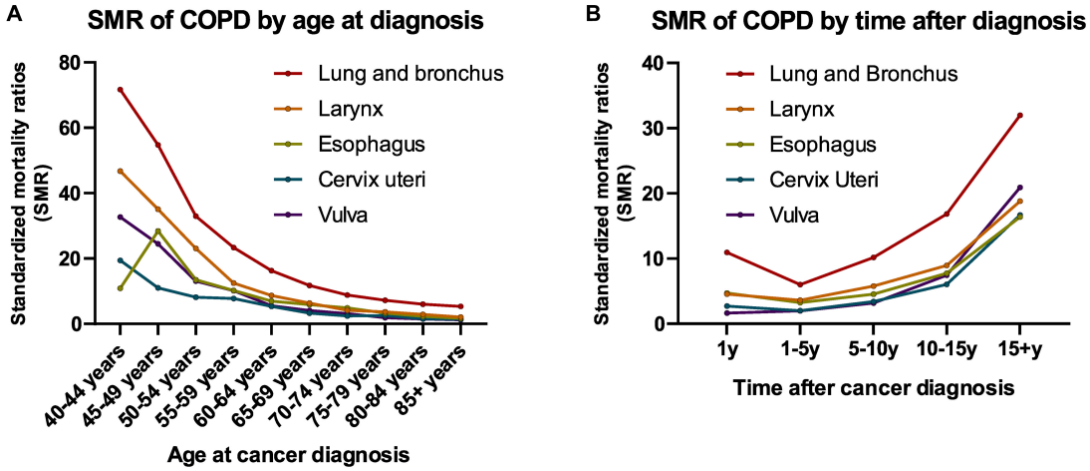
Trends of COPD mortality among patients diagnosed with cancer in SEER 18 registries by (**A**) age at diagnosis; (**B**) time after diagnosis.

Patients with cancer diagnosed at nearly all ages (all sites) had an increased risk of death due to COPD compared with the general population with the same age distribution in the USA (Figure 3A and Supplementary Figure 1). Patients aged 40–45 years had the highest risk of death from COPD than the general population (SMR, 10.84; 95% CI, 10.12–11.61) (Figure 3A and Supplementary Figure 1). The risk of death due to COPD in cancer survivors (all sites) gradually decreased as age at cancer diagnosis increased, and this trend was observed for both lung and extrapulmonary cancers (Figure 3A and Supplementary Figure 1). This was due to the increased risk of COPD death with increasing age in the general population. SMRs for risk of COPD death by age at cancer diagnosis for all sites are presented in Supplementary Figure 1.

Higher SMRs among patients with cancer were observed in female, black, and unmarried patients. The risk of COPD mortality was the highest in patients diagnosed between 1975 and 1989 and declined gradually thereafter. Patients living in the census tracts with a lower socioeconomic status (SES) level and a higher smoking prevalence were more likely to die from COPD (SMR, 2.32 and 2.27, respectively).

### Objective 2: COPD deaths by cancer type and stage

**Figure 4:**
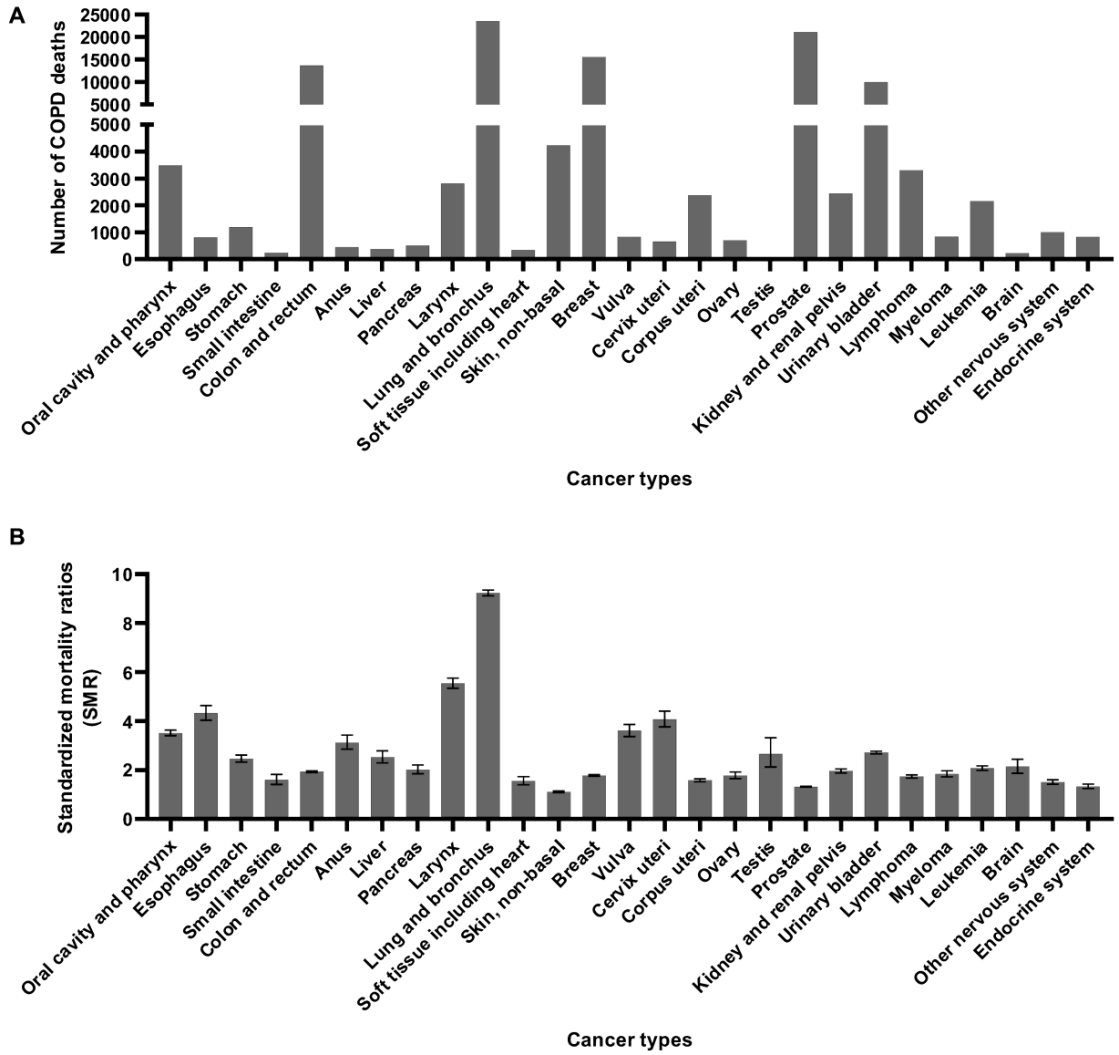
**COPD death number and SMR among patients diagnosed with cancer between 1975 and 2016 in SEER 18 registries by anatomic sites.** (**A**) COPD death number by anatomic site; (**B**) COPD SMR by anatomic site.

Most COPD deaths occurred in patients with lung, prostate, breast, colorectal, and bladder cancers, accounting for 70.0% of total deaths (Figure 4A). Compared with the general population with a similar demographic distribution, patients with lung cancer had the highest risk of dying from COPD, with an SMR of 9.23 (95% CI, 9.12–9.35), followed by those with laryngeal cancer (SMR, 5.54; 95% CI, 5.34–5.75), esophageal cancer (SMR, 4.33; 95% CI, 4.04–4.63), cervical cancer (SMR, 4.07; 95% CI, 4.06–4.40), vulva cancer (SMR, 3.61; 95% CI, 3.37–3.86), and cancers of the oral cavity and pharynx (SMR, 3.51; 95% CI, 3.40–3.63) (Figure 4B).

Analyses stratified by histology for lung cancer revealed that adenocarcinoma was associated with the lowest risk among all histological types, with an SMR of 5.72 (95% CI, 5.58–5.87). Tumor in the main bronchus was associated with the highest risk compared with other anatomic sites in the lung, with an SMR of 13.5 (95% CI, 12.6–14.4) (Table 2).

**Table 2:** COPD mortality among patients with lung cancer between 1975 and 2016 in SEER 18 registries by histology and anatomic sites.

**Characteristics**		**No. of patients with cancer (%)**	**Total person years of follow-up**	**Death from COPD**				SMR^*^ (95% CI)
				**Patients with cancer**		**General population**		
				**No. of observed deaths (%)**	**Mortality rates (per 100,000 person-years)**	**No. of expected deaths (%)**	**Mortality rates (per 100,000 person-years)**	
All lung cancer		965,341 (100%)	1,812,077	23,542 (100%)	1299.2	2,548	140.6	9.24 (9.12–9.36)
Histology							
	SCLC	135,702 (14.1%)	151,831	1,598 (6.8%)	1052.5	180	118.4	8.89 (8.47–9.34)
	NSCLC	829,639 (85.9%)	1,660,246	21,944 (93.2%)	1321.7	2,369	142.7	9.26 (9.14–9.39)
	AC	334,962 (34.7%)	846,564	6,101 (25.9%)	720.7	1,066	126.0	5.72 (5.58–5.87)
	SC	202,955 (21.0%)	417,356	6,969 (29.6%)	1669.8	669	160.2	10.4 (10.2–10.7)
	Other	291,722 (30.2%)	396,326	8,874 (37.7%)	2239.1	634	159.9	14.0 (13.7–14.3)
Site							
	Main bronchus	50,204 (5.2%)	57,763	864 (3.7%)	1495.8	64	110.8	13.5 (12.6–14.4)
	Upper lobe	465,097 (48.2%)	1,014,284	12,809 (54.4%)	1262.9	1,389	136.9	9.22 (9.06–9.38)
	Middle lobe	39,527 (4.1%)	93,545	1,020 (4.3%)	1090.4	122	130.0	8.39 (7.89–8.92)
	Lower lobe	218,819 (22.7%)	458,962	5,630 (23.9%)	1226.7	692	150.7	8.14 (7.93–8.36)
	Lung, NOS	191,694 (19.9%)	187,523	3,219 (13.7%)	1716.6	282	150.6	11.4 (11.0–11.8)

Abbreviations: COPD, chronic obstructive pulmonary disease; SMR, standardized mortality ratios; CI, confidence interval; SCLC, small cell lung cancer; NSCLC, non-small cell lung cancer; NOS, not otherwise specified.

^*^The SMRs were estimated as the ratios of observed to expected number of deaths. The observed values represented the number of COPD deaths in cancer patients, whereas the expected values represented the number of individuals who died of COPD in the general population, with a similar distribution of age, sex, race, and calendar year.

Deaths due to COPD were most commonly observed in patients with localized cancer (47.0%). Although there were only 10,987 (9.2%) patients with advanced disease, these patients had the highest risk of COPD mortality (SMR, 3.16; 95% CI, 3.10–3.22) (Table 3).

**Table 3:** COPD mortality among patients diagnosed with cancer between 1975 and 2016 in SEER 18 registries by cancer stage.

**Cancer type and stage**		**No. of patients (%)**	**Deaths from COPD**	
			**No. of observed deaths (%)**	SMR^*^ (95% CI)
All cancer patients				
	*In situ*	447,307 (5.7%)	5,942 (5.0%)	1.55 (1.51–1.59)
	Localized	2,938,939 (37.5%)	56,065 (47.0%)	1.90 (1.88–1.91)
	Regional	1,291,329 (16.5%)	19,633 (16.5%)	2.65 (2.62–2.69)
	Distant	1,376,743 (17.5%)	10,987 (9.2%)	3.16 (3.10–3.22)
	Unstaged	1,792,052 (22.8%)	26,601 (22.3%)	2.48 (2.45–2.51)
Lung cancer patients				
	*In situ*	723 (0.1%)	40 (0.2%)	10.8 (7.91–14.7)
	Localized	130,972 (13.7%)	7,484 (31.8%)	7.94 (7.77–8.13)
	Regional	188,902 (19.8%)	5,805 (24.7%)	8.51 (8.30–8.74)
	Distant	415,214 (43.4%)	4,812 (20.5%)	10.2 (9.91–10.5)
	Unstaged	220,635 (23.1%)	5,375 (22.9%)	12.0 (11.7–12.3)
Extrapulmonary cancer patients				
	*In situ*	446,584 (6.5%)	5,902 (6.2%)	1.54 (1.50–1.58)
	Localized	2,807,967 (40.8%)	48,581 (50.8%)	1.70 (1.68–1.71)
	Regional	1,102,427 (16.0%)	13,828 (14.4%)	2.06 (2.02–2.09)
	Distant	961,529 (14.0%)	6,175 (6.5%)	2.06 (2.01–2.11)
	Unstaged	1,571,417 (22.8%)	21,226 (22.2%)	2.07 (2.04–2.10)

Abbreviations: COPD, chronic obstructive pulmonary disease; SMR, standardized mortality ratios; CI, confidence interval.

^*^The SMRs were estimated as the ratios of observed to expected number of deaths. The observed values represented the number of COPD deaths in cancer patients, whereas the expected values represented the number of individuals who died of COPD in the general population, with a similar distribution of age, sex, race, and calendar year.

### Objective 3: COPD deaths during follow-up

For patients with a short-term cancer diagnosis, COPD deaths were more likely to occur in those with lung cancer, with nearly 40% of all COPD deaths occurring in patients with lung cancer within the first year after a cancer diagnosis. In long-term cancer survivors, deaths due to COPD were more likely to occur in patients with prostate cancer and breast cancer (nearly 35% of all COPD deaths in patients surviving more than 5 years) (Supplementary Figure 2).

The COPD mortality risk in cancer survivors was higher than that in the general population over the entire follow-up after cancer diagnosis (Figure 3B and Supplementary Figure 3). An increasing trend for risk of COPD death was observed in patients with cancer surviving more than 5 years, and the highest risk of COPD mortality was observed after a long-term follow-up of more than 15 years. Patients with lung cancer had a relatively high SMR of 10.9 (95% CI, 10.7–11.12) in the first year after diagnosis (Figure 3B and Supplementary Figure 3). This SMR decreased to 6.02 (95% CI, 5.87–6.16) after 5 years of follow-up and increased again throughout survivorship. The SMRs for risk of COPD death in all cancer types according to follow-up time after a cancer diagnosis are presented in Supplementary Figure 3.

## DISCUSSION

This study's findings revealed that patients with cancer are at increased risk of death due to COPD and highlight the importance of respiratory care throughout cancer survivorship. Previous studies have mainly focused on COPD's impact on cancer mortality [20, 21]. However, the relationship between cancer and COPD mortality remains unclear. Our study provided a large and comprehensive characterization of COPD mortality in patients with cancer, using a population-based cancer registry across 28 cancer sites and including 40 years of data.

Our results suggest that COPD prevention strategies should be aimed at patients with lung, prostate, breast, colorectal, and bladder cancers. Although less common, certain types of cancers of the head and neck and genitourinary system (particularly, the larynx, oral cavity, pharynx, cervix uteri, and vulva) are associated with a higher risk of death due to COPD; this information may be useful for clinicians and PCPs to develop targeted prevention strategies.

Lung cancer and COPD are associated with cigarette smoking and often occur as comorbidities. There is increasing evidence linking the diseases beyond this common mechanism, such as premature aging, genetic predisposition, telomere shortening, mitochondrial dysfunction, and epigenetic changes [5, 22]. In this study, lung cancer was identified as the cancer type with the highest risk of COPD mortality. Furthermore, COPD was the second most common cause of non-cancer deaths in patients with lung cancer. These two diseases are likely to place a considerable burden on health services in the future, and our data underscore the importance of the close involvement of pulmonologists and PCPs with patients with lung cancer throughout survivorship. Notably, the observed very high risk of dying from COPD within the first year after lung cancer diagnosis supports pulmonologists' early involvement in treating such patients.

We found that patients with cancers of the larynx had the highest mortality rate amongst those with cancers of the extrapulmonary organs. Cigarette smoking is also a significant risk factor for laryngeal cancer. Two studies have reported a relatively high prevalence of COPD in patients with laryngeal cancer [7, 23] and suggested that COPD might be associated with worse survival rates. Other smoking-related cancers, including esophageal and oropharyngeal cancers, have also been associated with a higher SMR. Some genitourinary malignancies, such as cervical, vulva, and bladder cancers, were identified as having a relatively higher risk of death from COPD. Further in-depth studies are necessary to explore this association. Furthermore, we observed that cancer stages were associated with the COPD mortality risk, highlighting the impact and burden of cancer on COPD outcomes.

Our work also evaluated the SMRs of COPD as a function of age at diagnosis and follow-up time after a cancer diagnosis. Young patients had a very high risk of COPD mortality compared with the age-matched general population. This was in agreement with previous studies showing that young adults with cancer have an elevated mortality burden from non-cancer causes [24, 25]. In patients with lung cancer, we observed a peak window of COPD deaths within the first year of diagnosis. This finding may be due to the impact of aggressive treatment, such as chest surgery and chest radiation therapy, or anti-cancer drugs with pulmonary toxicity. Another plausible explanation is that the diagnosis of cancer could have been made incidentally when treating severe or fatal co-existing COPD. For nearly all cancers, the risk of death from COPD began to increase by 5 years after diagnosis, and this trend continued to increase with follow-up time. This can be partly explained by the fact that patients with cancer are more prone to developing COPD with aging and lung function impairment [7]. Other characteristics associated with a higher risk of COPD deaths in patients with cancer, such as black ethnicity, high smoking prevalence, low SES level, and unmarried status, were similar to those in the general population [26–29].

Our study had some limitations. First, there is a risk of reporting bias in death certificates leading to misclassification of the causes of death [30, 31]. The SEER (Surveillance, Epidemiology, and End Results) mortality data were provided by the National Center for Health Statistics and National Vital Statistics System. Systematic and standardized data collection procedures are used to ensure that the causes of death recorded in SEER are accurate [32]. Previous studies also examined the validity and reliability of death certificates in SEER and found acceptable results [33, 34]. Second, SEER does not contain information regarding pre-existing comorbidities, performance status, quality of life, lung function, or detailed and complete cancer treatment information. Thus, we could not analyze the cause-and-effect relationship between different risk factors and COPD. Nevertheless, analysis of the SEER database's extensive available data remains a powerful, useful, and integral tool in exploratory medical research [32]. Third, smoking status and SES were not available at an individual level. Consequently, we used an area-based measure of adult smoking prevalence or SES level that was available in SEER as an approximation [35]. Fourth, the study was based on mortality data of COPD in patients with cancer and the relevant general population; thus, we could not analyze COPD's influence on lung and extrapulmonary cancers. We recommend further studies to fill in the gaps. To reduce the impact of this limitation, we extracted similar sub-groups from cancer population and general population, and then compared the COPD morality between these two groups to evaluate the impact of cancer diagnosis to COPD mortality. This approach hypothesized that factors other than cancer diagnosis were highly similar in this population. It cannot eliminate the impact of time difference, but can reduce them.

## CONCLUSIONS

This study's findings show that patients with cancer are at an increased risk of dying from COPD. The COPD mortality risk is much higher in patients with lung cancer than in those with extrapulmonary cancers. Among all extrapulmonary cancers, laryngeal and esophageal cancers had the highest COPD mortality risk, which rises with time. Our results suggest a need for enhanced, coordinated multidisciplinary care between oncologists, pulmonologists, and PCPs throughout cancer survivorship.

## MATERIALS AND METHODS

### Data sources and study population

A retrospective cohort study was performed using data from the SEER program. This program involves population-based cancer registries from the National Cancer Institute and routinely collects and reports data on cancer demographics, incidence, follow-up data, anatomic sites, morphology, stage, therapy, and SES of patients with cancer in the US [36].

All patients diagnosed with cancer between 1975 and 2016 were identified from the SEER 18 database (2019 submission) using SEER*Stat software, version 8.3.6 [37]. Data from patients with only one type of cancer or those with a first primary cancer were included. Patients were excluded if their diagnosis was obtained exclusively from death certificates or autopsy reports. We further excluded patients without complete follow-up information, including data on follow-up duration, age at diagnosis, or race. Given that COPD mainly occurred in elderly patients, we also excluded patients younger than 40 years of age. For comparison with the general population, mortality data for the general US population registered in the National Center for Health Statistics between 1975 and 2016 were also obtained from the SEER database [37] (Supplementary Table 3).

Since the SEER is a publicly available database, access to the data required a signed research data agreement form. Institutional review board approval and the need for informed consent were waived for data obtained from the SEER database, as the study did not involve human subjects and all data were anonymized.

### Definition of variables

All patients were observed from the time of cancer diagnosis until death, exiting the study, or until the end of the study (December 31, 2016). Death from COPD was chosen as the event of interest. We evaluated the following variables for the patients included in this study: age at diagnosis, sex, race, year of diagnosis, marital status, survival (in months), cause of death, anatomic site of the cancer, cancer stage, surgical therapy, chemotherapy, radiotherapy, SES, and smoking prevalence.

Patients with the cause of death coded as "Chronic Obstructive Pulmonary Disease and Allied Conditions (50130)" were considered to have died due to COPD. The cause of death variable for COPD was 490–493 and 519.3 in the ICD-8 (International Statistical Classification of Diseases and Related Health Problems, eighth revision) codes for cases diagnosed between 1975 to 1978, 490–496 in the ICD-9 for cases diagnosed between 1979 and 1998, and J40-J47 in the ICD-10 for cases diagnosed between 1999 and 2016.

The SES of patients was measured using Yost *et al*.'s census tract-level composite SES index provided by SEER for patients diagnosed between 1990 and 2016, formulated using a principal components analysis on the SES measures [38, 39]. An SES index is a complex matrix of seven aspects of SES information, including the median household income, median house value, median gross rent, percentage of the population below 150% of the poverty level, education index, percentage of working class, and percentage of unemployed workers. The SES scores were further divided into tertiles, as previously described [39].

Smoking prevalence (percentage of current smokers in the population aged 18 years or above) was determined with model-based small area estimation techniques using data from national surveys and multiple related sources [35]. The smoking prevalence was estimated at various time points between 1997 and 2010; thus, the cases were restricted to this period when assessing the impact of smoking prevalence. The smoking prevalence was further divided into tertiles for analysis.

As the SEER database records the duration of survival in months and a month was the shortest time interval available for analysis, survival durations shorter than 1 month were recorded as 0 months in the SEER program. Therefore, according to standard epidemiologic conventions, patients with durations of survival coded as 0 were converted to half-a-month periods [40].

### Statistical analyses

The COPD mortality rates were calculated as the number of deaths due to COPD divided by person-years of follow-up. The SMRs and corresponding 95% CIs of non-cancer deaths were calculated according to previously published methods [32, 40–42]. The SMRs were estimated as the ratios of observed deaths to the expected number of deaths. The observed values represented the number of COPD deaths in patients with cancer, whereas the expected values represented the number of individuals who died of COPD in the general population, given a similar distribution of age, sex, race, and calendar year. For the standardization of age and calendar year, 5-year-categories were created, and the values at the time of diagnosis were adopted. The race groups were defined as white, black, and other. The relative demographic distribution of the patients with cancer and general population are shown in Supplementary Tables 3 and 4. The 95% CIs of the SMRs were obtained using an approximation from the Poisson distribution [40, 43]. For objectives 1, 2, and 3, we describe the risk of death from COPD as a function of demographic characteristics, cancer types and stages, and follow-up time after a cancer diagnosis.

All statistical tests were two-sided, and *P* < .05 was considered to indicate statistical significance. Analyses were performed using SEER*Stat software, version 8.3.6 (US Department of Health and Human Services) and the R version 3.52 (The R Project for Statistical Computing) statistical software package [37, 44].

## Supplementary Materials

Supplementary Material

Supplementary Figures

Supplementary Tables 1-2

Supplementary Table 3

Supplementary Table 4
