# Supplementary material for: Deaths from COPD in patients with cancer: a population-based study
Source: Aging (Albany NY). 2021 Apr 27;13(9):12641–59. doi: 10.18632/aging.202939 (PMC8148461; doi:10.18632/aging.202939)
Supplement: Supplementary Material [file aging-13-202939-s001.pdf]

SUPPLEMENTARY MATERIALS

The calculation of standardized mortality ratio (SMR)

To facilitate a comparison between the cancer population and the general population, we used the so-called indirect method of adjustment to calculate the SMR [1–4]. The progress of calculation was as follow:

(1) Choose a reference or standard population: in this study, general US population collected by the National Center for Health Statistics between 1975 and 2016 were chosen as reference or standard population [5].

(2) Choose variables for standardization: four variables, age, sex, race and calendar year were chosen as variables used for standardization. For age, five-year categories were created in the course of standardization. Thus age was divided into 19 groups: "0 years", "1–4 years", "5–9 years", "10–14 years", "15–19 years", "20–24 years", "25–29 years", "30–34 years", "35–39 years", "40–44 years", "45–49 years", "50–54 years", "55–59 years", "60–64 years", "65–69 years", "70–74 years", "75–79 years", "80–84 years", "85+ years". Sex were characterized as two group: "female" and "male". If not specifically addressed, races were characterized as three categories: white, black and other. The "Other" group included the less frequent races: "Asian or Pacific Islander", "American Indian/Alaska Native" and so on [6]. Calendar year of cancer diagnosis was also divided into five-years categories: "1975–1979", "1980–1984", "1985–1989", "1990–1994", "1995–1999", "2000–2004", "2005–2009", "2010–2016".

(3) Divide the cancer patients and general population into subgroups: both cancer patients and the general population were divided into subgroups characterized by the above variables for standardization. For each subgroup in cancer patients, total survival time (person-years) were calculated. Besides, the mortality rates for each subgroup of the chosen reference population, namely the general population abstracted from mortality data collected by the National Center for Health Statistics spanning 1975 to 2016 and accessed through the SEER program.

(4) Calculate the observed number of deaths in cancer patients: observed number of deaths represents the total number of deaths from certain causes in cancer patients recorded during the study period.

(5) Calculate the expected number of deaths: the expected number of deaths for each subgroup of population was the product of corresponding observed person-years in this group of the cancer patients and the corresponding mortality rate in the comparable subgroup of the reference population. The total expected number of deaths was then a sum of the expected number of deaths for each subgroup of population.

(6) Calculate SMR: divide the total number of observed number of deaths by the expected number of deaths:

$$\text{SMR} = \frac{\text{Observed number of deaths}}{\text{Expected number of deaths}} = \frac{\text{Observed}}{\text{Expected}}$$

A SMR greater than 1.00 indicates that more mortality has occurred in cancer patients than expected, while a ratio less than 1.00 indicates that less mortality has occurred.

(7) Calculate 95% confidence intervals (CI): 95% CIs were obtained using an approximation based on an assumption of a Poisson regression model for mortality. 95% CI of SMR were obtained using an approximate Wald confidence limits from a Poisson distribution. The Rothman Greenland Method were used [3]:

$$\text{Lower limit} = \exp[\ln(\text{SMR}) - Z_{1-\alpha/2}(1/\text{Observed})^{\frac{1}{2}}]$$

$$\text{Upper limit} = \exp[\ln(\text{SMR}) + Z_{1-\alpha/2}(1/\text{Observed})^{\frac{1}{2}}]$$

Where (1-α/2) represents the desired confidence percentage for interval estimation, and $Z_{1-\alpha/2}$ represents the number such that the chance that a standard normal variable falls between $-Z_{1-\alpha/2}$ and $Z_{1-\alpha/2}$ is $(1-\alpha/2)$ % (e.g. $Z_{95} = 1.96$ and $Z_{99} = 2.58$). ln(SMR) represents the natural logarithm of SMR. Observed represents the observed number of deaths in cancer patients.

REFERENCES

1. Hall DJ. Standardization and life tables. Islamic World Medical Journal. 1984; 14:22–25.
2. Naing NN. Easy way to learn standardization: direct and indirect methods. Malays J Med Sci. 2000; 7:10–15. PMID:22844209
3. Rothman KJ, Greenland S, Lash TL. (2008). Modern Epidemiology. (Philadelphia, PA: Lippincott Williams & Wilkins).
4. Ulm K. A simple method to calculate the confidence interval of a standardized mortality ratio (SMR). Am J Epidemiol. 1990; 131:373–75.

https://doi.org/10.1093/oxfordjournals.aje.a115507 PMID:2296988

5. Cortopassi F, Gurung P, Pinto-Plata V. Chronic Obstructive Pulmonary Disease in Elderly Patients. Clin Geriatr Med. 2017; 33:539–52. https://doi.org/10.1016/j.cger.2017.06.006 PMID:28991649

6. Surveillance, Epidemiology, and End Results Program. Race recode changes for the 1973–2005 SEER research data (November 2007 submission) and later releases. https://seer.cancer.gov/seerstat/variables/seer/race_ethnicity/. Published March 2017. Accessed July 15, 2019.
