# Supplementary material for: Deaths from COPD in patients with cancer: a population-based study
Source: Aging (Albany NY). 2021 Apr 27;13(9):12641–59. doi: 10.18632/aging.202939 (PMC8148461; doi:10.18632/aging.202939)
Supplement: Supplementary Figures [file aging-13-202939-s002.pdf]

SUPPLEMENTARY FIGURES

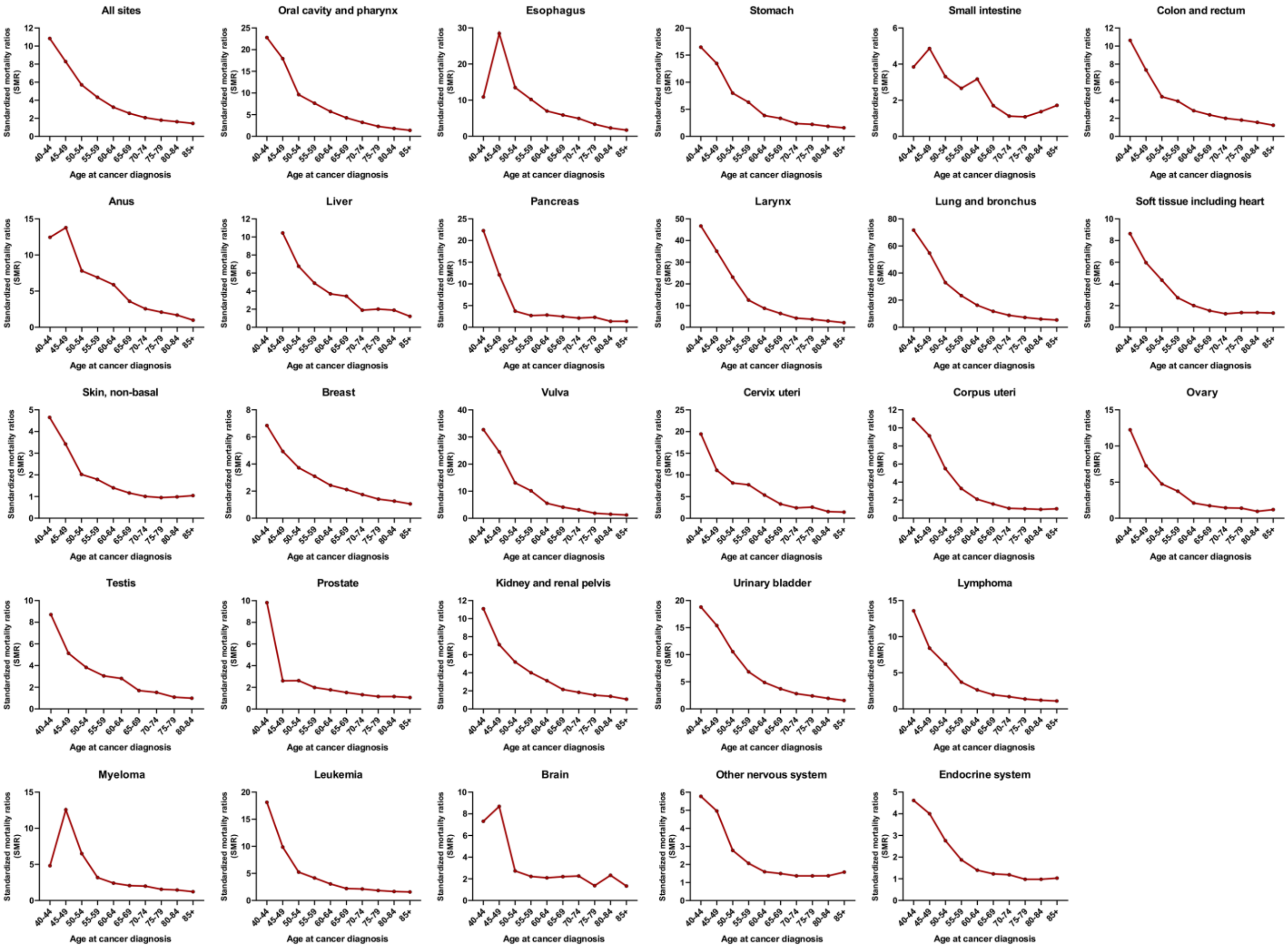

Supplementary Figure 1. Trends of COPD mortality among patients diagnosed cancer in SEER 18 registries by age at cancer diagnosis and anatomic sites.

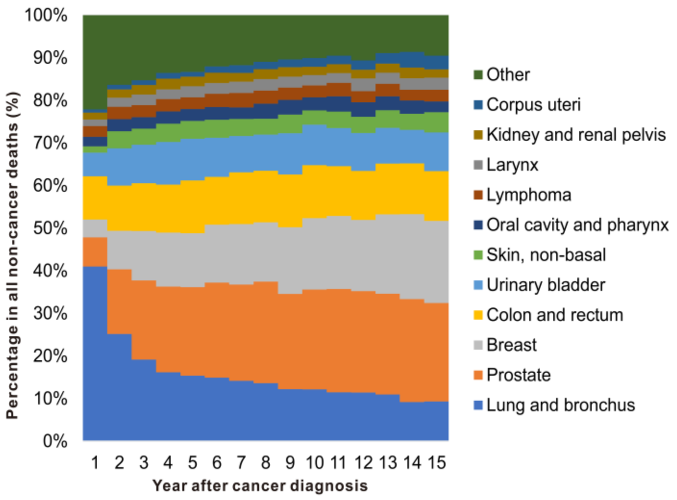

Supplementary Figure 2. Site distribution of COPD deaths among patients diagnosed with cancer in SEER 18 registries by time after diagnosis.

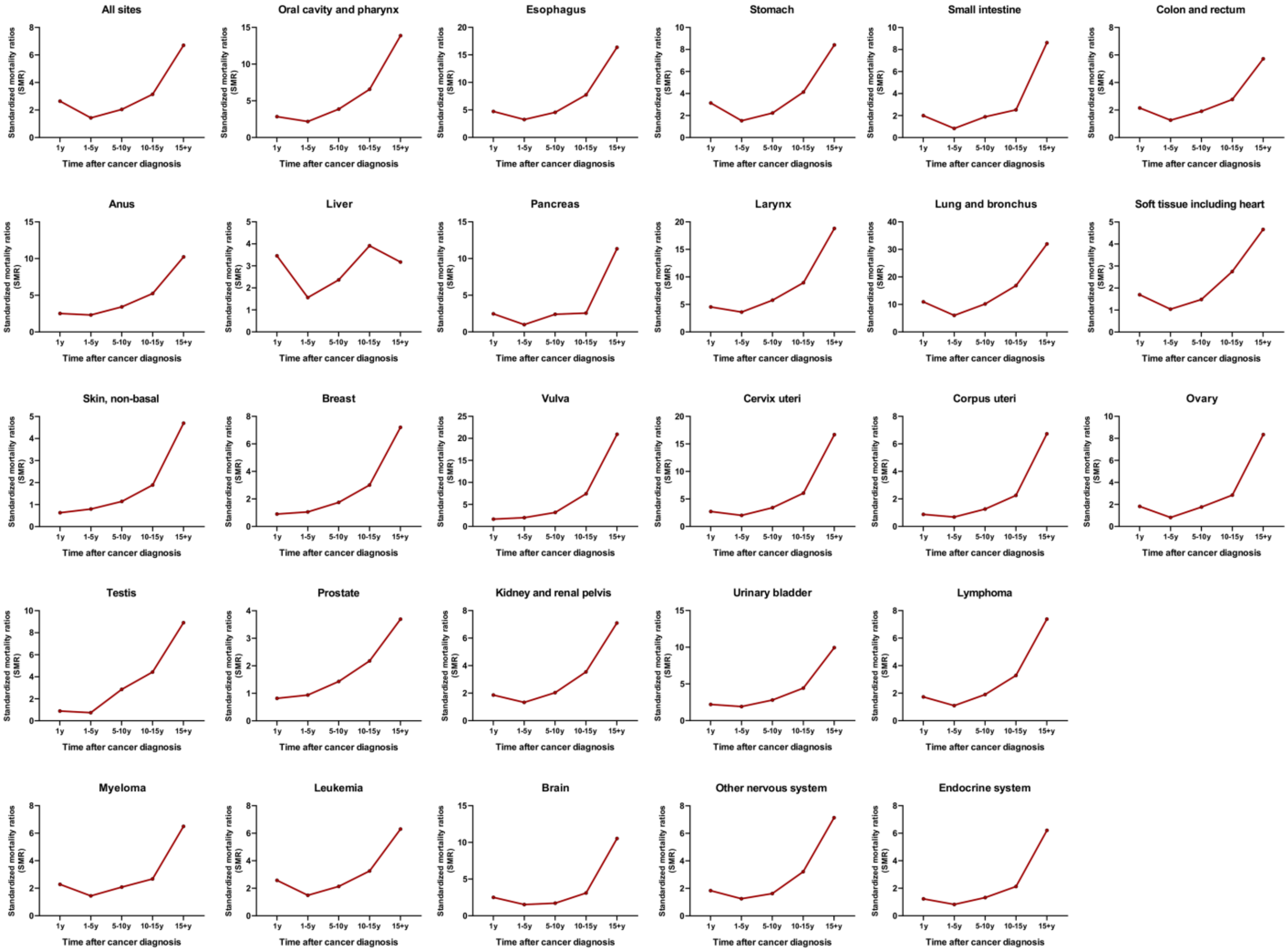

Supplementary Figure 3. Trends of COPD mortality among patients diagnosed cancer in SEER 18 registries by time from cancer diagnosis and anatomic sites.
