# Supplementary material for: Deaths from COPD in patients with cancer: a population-based study
Source: Aging (Albany NY). 2021 Apr 27;13(9):12641–59. doi: 10.18632/aging.202939 (PMC8148461; doi:10.18632/aging.202939)
Supplement: Supplementary Tables 1-2 [file aging-13-202939-s003.pdf]

SUPPLEMENTARY TABLES

Supplementary Table 1. Non-cancer causes of death among patients diagnosed with cancer between 1975 and 2016 in SEER 18 registries.

Cause of death	No. of deaths	Percentage
Total	1,436,158	100%
Diseases of heart	543,917	37.87%
Chronic obstructive pulmonary disease and allied conditions	119,228	8.30%
Cerebrovascular diseases	116,642	8.12%
Pneumonia and influenza	61,056	4.25%
Alzheimer’s	51,087	3.56%
Diabetes mellitus	47,823	3.33%
Other infectious and parasitic diseases including HIV	40,991	2.85%
Accidents and adverse effects	33,671	2.34%
Nephritis, nephrotic syndrome and nephrosis	30,666	2.14%
Septicemia	28,166	1.96%
Chronic liver disease and cirrhosis	20,052	1.40%
Hypertension without heart disease	19,984	1.39%
Symptoms, signs and ill-defined conditions	19,213	1.34%
Suicide and self-inflicted injury	11,956	0.83%
Atherosclerosis	11,659	0.81%
Aortic aneurysm and dissection	10,017	0.70%
Other diseases of arteries, arterioles, capillaries	7,549	0.53%
Stomach and duodenal ulcers	4,651	0.32%
Congenital anomalies	1,790	0.12%
Homicide and legal intervention	1,209	0.08%
Tuberculosis	690	0.05%
Complications of pregnancy, childbirth, puerperium	214	0.01%
Certain conditions originating in perinatal period	61	0.004%
Syphilis	25	0.002%
Other cause of death	253,841	17.68%

Supplementary Table 2. Non-cancer causes of death among patients diagnosed with lung cancer between 1975 and 2016 in SEER 18 registries.

Cause of death	No. of deaths	Percentage
Total	110,404	100%
Diseases of heart	39,306	35.60%
Chronic obstructive pulmonary disease and allied conditions	23,516	21.30%
Cerebrovascular diseases	6,810	6.17%
Pneumonia and influenza	5,279	4.78%
Accidents and adverse effects	2,632	2.38%
Septicemia	2,285	2.07%
Diabetes mellitus	2,149	1.95%
Other infectious and parasitic diseases including HIV	1,758	1.59%
Symptoms, signs and ill-defined conditions	1,747	1.58%
Nephritis, nephrotic syndrome and nephrosis	1,647	1.49%
Alzheimer’s	1,438	1.30%

Suicide and self-inflicted injury	1,236	1.12%
Hypertension without heart disease	1,011	0.92%
Aortic aneurysm and dissection	987	0.89%
Chronic liver disease and cirrhosis	908	0.82%
Atherosclerosis	688	0.62%
Other diseases of arteries, arterioles, capillaries	587	0.53%
Stomach and duodenal ulcers	472	0.43%
Tuberculosis	111	0.10%
Congenital anomalies	103	0.09%
Homicide and legal intervention	73	0.07%
Complications of pregnancy, childbirth, puerperium	30	0.03%
Certain conditions originating in perinatal period	15	0.01%
Syphilis	2	0.002%
Other cause of death	15,614	14.14%

Please browse Full Text version to see the data of Supplementary Tables 3 and 4.

Supplementary Table 3. COPD mortality in US general population by age at diagnosis, sex, race and calendar year of diagnosis.

Supplementary Table 4. COPD mortality in all patients with cancer by age at diagnosis, sex, race and calendar year of diagnosis.
