# Supplementary material for: Deaths from COPD in patients with cancer: a population-based study
Source: Aging (Albany NY). 2021 Apr 27;13(9):12641–59. doi: 10.18632/aging.202939 (PMC8148461; doi:10.18632/aging.202939)
Supplement: Supplementary Table 3 [file aging-13-202939-s004.pdf]

Supplementary Table 3. COPD mortality in US general population by age at diagnosis, sex, race and calendar year of diagnosis.

Age at diagnosis	Sex	Race	Calendar year of diagnosis	Mortality rates (per 100,000 person-years)	No. of deaths from COPD	Population
00 years	Male and female	All races	1975-1979	2.4	388	16,306,151
00 years	Male and female	All races	1980-1984	1.6	287	18,033,145
00 years	Male and female	All races	1985-1989	1.4	268	18,697,186
00 years	Male and female	All races	1990-1994	1.4	266	19,694,347
00 years	Male and female	All races	1995-1999	1.1	206	18,846,097
00 years	Male and female	All races	2000-2004	0.9	182	19,810,204
00 years	Male and female	All races	2005-2009	0.8	165	20,330,450
00 years	Male and female	All races	2010-2016	0.7	186	27,666,515
00 years	Male and female	White	1975-1979	2.0	275	13,501,735
00 years	Male and female	White	1980-1984	1.3	189	14,719,150
00 years	Male and female	White	1985-1989	1.2	182	15,029,517
00 years	Male and female	White	1990-1994	1.1	165	15,562,635
00 years	Male and female	White	1995-1999	0.8	113	14,876,288
00 years	Male and female	White	2000-2004	0.8	116	15,397,897
00 years	Male and female	White	2005-2009	0.6	95	15,451,285
00 years	Male and female	White	2010-2016	0.5	104	20,758,459
00 years	Male and female	Black	1975-1979	4.1	99	2,393,131
00 years	Male and female	Black	1980-1984	3.4	91	2,688,294
00 years	Male and female	Black	1985-1989	2.6	74	2,880,836
00 years	Male and female	Black	1990-1994	2.9	93	3,157,537
00 years	Male and female	Black	1995-1999	2.8	83	2,944,800
00 years	Male and female	Black	2000-2004	1.9	61	3,142,168
00 years	Male and female	Black	2005-2009	1.7	59	3,387,505
00 years	Male and female	Black	2010-2016	1.4	68	4,721,339
00 years	Male and female	Other	1975-1979	3.4	14	411,285
00 years	Male and female	Other	1980-1984	0.0	0	625,701
00 years	Male and female	Other	1985-1989	1.5	12	786,833
00 years	Male and female	Other	1990-1994	0.0	0	974,175
00 years	Male and female	Other	1995-1999	1.0	10	1,025,009
00 years	Male and female	Other	2000-2004	0.0	0	1,270,139
00 years	Male and female	Other	2005-2009	0.7	11	1,491,660
00 years	Male and female	Other	2010-2016	0.6	14	2,186,717
00 years	Male	All races	1975-1979	3.0	248	8,344,898
00 years	Male	All races	1980-1984	2.0	180	9,225,369
00 years	Male	All races	1985-1989	1.9	182	9,565,111
00 years	Male	All races	1990-1994	1.6	163	10,078,032

00 years	Male	All races	1995-1999	1.3	128	9,651,025
00 years	Male	All races	2000-2004	1.1	115	10,127,688
00 years	Male	All races	2005-2009	1.0	103	10,388,610
00 years	Male	All races	2010-2016	0.9	123	14,148,332
00 years	Male	White	1975-1979	2.6	177	6,927,665
00 years	Male	White	1980-1984	1.6	118	7,553,162
00 years	Male	White	1985-1989	1.7	130	7,709,086
00 years	Male	White	1990-1994	1.3	100	7,982,023
00 years	Male	White	1995-1999	1.0	74	7,633,307
00 years	Male	White	2000-2004	1.0	78	7,881,308
00 years	Male	White	2005-2009	0.7	55	7,905,435
00 years	Male	White	2010-2016	0.6	67	10,624,492
00 years	Male	Black	1975-1979	5.3	64	1,207,966
00 years	Male	Black	1980-1984	4.2	57	1,353,955
00 years	Male	Black	1985-1989	3.0	44	1,454,048
00 years	Male	Black	1990-1994	3.6	57	1,597,902
00 years	Male	Black	1995-1999	3.4	50	1,492,176
00 years	Male	Black	2000-2004	2.1	33	1,596,625
00 years	Male	Black	2005-2009	2.4	41	1,720,349
00 years	Male	Black	2010-2016	1.8	43	2,402,525
00 years	Male	Other	1975-1979	0.0	0	209,267
00 years	Male	Other	1980-1984	0.0	0	318,252
00 years	Male	Other	1985-1989	0.0	0	401,977
00 years	Male	Other	1990-1994	0.0	0	498,107
00 years	Male	Other	1995-1999	0.0	0	525,542
00 years	Male	Other	2000-2004	0.0	0	649,755
00 years	Male	Other	2005-2009	0.0	0	762,826
00 years	Male	Other	2010-2016	1.2	13	1,121,315
00 years	Female	All races	1975-1979	1.8	140	7,961,253
00 years	Female	All races	1980-1984	1.2	107	8,807,776
00 years	Female	All races	1985-1989	0.9	86	9,132,075
00 years	Female	All races	1990-1994	1.1	103	9,616,315
00 years	Female	All races	1995-1999	0.8	78	9,195,072
00 years	Female	All races	2000-2004	0.7	67	9,682,516
00 years	Female	All races	2005-2009	0.6	62	9,941,840
00 years	Female	All races	2010-2016	0.5	63	13,518,183
00 years	Female	White	1975-1979	1.5	98	6,574,070
00 years	Female	White	1980-1984	1.0	71	7,165,988
00 years	Female	White	1985-1989	0.7	52	7,320,431
00 years	Female	White	1990-1994	0.9	65	7,580,612
00 years	Female	White	1995-1999	0.5	39	7,242,981
00 years	Female	White	2000-2004	0.5	38	7,516,589
00 years	Female	White	2005-2009	0.5	40	7,545,850

00 years	Female	White	2010-2016	0.4	37	10,133,967
00 years	Female	Black	1975-1979	3.0	35	1,185,165
00 years	Female	Black	1980-1984	2.5	34	1,334,339
00 years	Female	Black	1985-1989	2.1	30	1,426,788
00 years	Female	Black	1990-1994	2.3	36	1,559,635
00 years	Female	Black	1995-1999	2.3	33	1,452,624
00 years	Female	Black	2000-2004	1.8	28	1,545,543
00 years	Female	Black	2005-2009	1.1	18	1,667,156
00 years	Female	Black	2010-2016	1.1	25	2,318,814
00 years	Female	Other	1975-1979	0.0	0	202,018
00 years	Female	Other	1980-1984	0.0	0	307,449
00 years	Female	Other	1985-1989	0.0	0	384,856
00 years	Female	Other	1990-1994	0.0	0	476,068
00 years	Female	Other	1995-1999	0.0	0	499,467
00 years	Female	Other	2000-2004	0.0	0	620,384
00 years	Female	Other	2005-2009	0.0	0	728,834
00 years	Female	Other	2010-2016	0.0	0	1,065,402
01-04 years	Male and female	All races	1975-1979	0.5	301	62,826,784
01-04 years	Male and female	All races	1980-1984	0.4	253	67,780,270
01-04 years	Male and female	All races	1985-1989	0.3	218	71,862,312
01-04 years	Male and female	All races	1990-1994	0.3	264	77,404,324
01-04 years	Male and female	All races	1995-1999	0.3	232	77,701,818
01-04 years	Male and female	All races	2000-2004	0.3	262	77,473,829
01-04 years	Male and female	All races	2005-2009	0.3	276	80,167,440
01-04 years	Male and female	All races	2010-2016	0.3	363	112,174,133
01-04 years	Male and female	White	1975-1979	0.4	213	51,859,143
01-04 years	Male and female	White	1980-1984	0.3	149	55,260,254
01-04 years	Male and female	White	1985-1989	0.2	139	57,985,768
01-04 years	Male and female	White	1990-1994	0.2	134	61,142,729
01-04 years	Male and female	White	1995-1999	0.2	117	60,715,402
01-04 years	Male and female	White	2000-2004	0.2	124	60,169,051
01-04 years	Male and female	White	2005-2009	0.2	131	61,405,358
01-04 years	Male and female	White	2010-2016	0.2	166	84,136,965
01-04 years	Male and female	Black	1975-1979	0.9	82	9,345,966
01-04 years	Male and female	Black	1980-1984	1.0	97	10,185,424
01-04 years	Male and female	Black	1985-1989	0.7	74	10,813,686
01-04 years	Male and female	Black	1990-1994	0.9	117	12,526,173
01-04 years	Male and female	Black	1995-1999	0.8	101	12,754,577
01-04 years	Male and female	Black	2000-2004	1.0	122	12,474,033
01-04 years	Male and female	Black	2005-2009	1.0	131	12,950,678
01-04 years	Male and female	Black	2010-2016	0.9	169	19,035,900
01-04 years	Male and female	Other	1975-1979	0.0	0	1,621,675
01-04 years	Male and female	Other	1980-1984	0.0	0	2,334,592

01-04 years	Male and female	Other	1985-1989	0.0	0	3,062,858
01-04 years	Male and female	Other	1990-1994	0.3	13	3,735,422
01-04 years	Male and female	Other	1995-1999	0.3	14	4,231,839
01-04 years	Male and female	Other	2000-2004	0.3	16	4,830,745
01-04 years	Male and female	Other	2005-2009	0.2	14	5,811,404
01-04 years	Male and female	Other	2010-2016	0.3	28	9,001,268
01-04 years	Male	All races	1975-1979	0.6	178	32,115,668
01-04 years	Male	All races	1980-1984	0.4	151	34,662,039
01-04 years	Male	All races	1985-1989	0.3	116	36,766,042
01-04 years	Male	All races	1990-1994	0.4	168	39,608,837
01-04 years	Male	All races	1995-1999	0.4	144	39,757,266
01-04 years	Male	All races	2000-2004	0.4	169	39,604,862
01-04 years	Male	All races	2005-2009	0.4	160	40,959,583
01-04 years	Male	All races	2010-2016	0.4	229	57,318,292
01-04 years	Male	White	1975-1979	0.5	127	26,584,490
01-04 years	Male	White	1980-1984	0.3	82	28,341,746
01-04 years	Male	White	1985-1989	0.2	73	29,745,100
01-04 years	Male	White	1990-1994	0.3	87	31,356,441
01-04 years	Male	White	1995-1999	0.2	64	31,135,325
01-04 years	Male	White	2000-2004	0.2	75	30,825,855
01-04 years	Male	White	2005-2009	0.2	77	31,426,329
01-04 years	Male	White	2010-2016	0.2	103	43,043,142
01-04 years	Male	Black	1975-1979	1.0	49	4,710,334
01-04 years	Male	Black	1980-1984	1.3	66	5,138,116
01-04 years	Male	Black	1985-1989	0.8	41	5,466,612
01-04 years	Male	Black	1990-1994	1.1	71	6,340,461
01-04 years	Male	Black	1995-1999	1.1	70	6,469,374
01-04 years	Male	Black	2000-2004	1.4	86	6,338,277
01-04 years	Male	Black	2005-2009	1.2	76	6,584,168
01-04 years	Male	Black	2010-2016	1.1	107	9,682,012
01-04 years	Male	Other	1975-1979	0.0	0	820,844
01-04 years	Male	Other	1980-1984	0.0	0	1,182,177
01-04 years	Male	Other	1985-1989	0.0	0	1,554,330
01-04 years	Male	Other	1990-1994	0.5	10	1,911,935
01-04 years	Male	Other	1995-1999	0.5	10	2,152,567
01-04 years	Male	Other	2000-2004	0.0	0	2,440,730
01-04 years	Male	Other	2005-2009	0.0	0	2,949,086
01-04 years	Male	Other	2010-2016	0.4	19	4,593,138
01-04 years	Female	All races	1975-1979	0.4	123	30,711,116
01-04 years	Female	All races	1980-1984	0.3	102	33,118,231
01-04 years	Female	All races	1985-1989	0.3	102	35,096,270
01-04 years	Female	All races	1990-1994	0.3	96	37,795,487
01-04 years	Female	All races	1995-1999	0.2	88	37,944,552

01-04 years	Female	All races	2000-2004	0.2	93	37,868,967
01-04 years	Female	All races	2005-2009	0.3	116	39,207,857
01-04 years	Female	All races	2010-2016	0.2	134	54,855,841
01-04 years	Female	White	1975-1979	0.3	86	25,274,653
01-04 years	Female	White	1980-1984	0.2	67	26,918,508
01-04 years	Female	White	1985-1989	0.2	66	28,240,668
01-04 years	Female	White	1990-1994	0.2	47	29,786,288
01-04 years	Female	White	1995-1999	0.2	53	29,580,077
01-04 years	Female	White	2000-2004	0.2	49	29,343,196
01-04 years	Female	White	2005-2009	0.2	54	29,979,029
01-04 years	Female	White	2010-2016	0.2	63	41,093,823
01-04 years	Female	Black	1975-1979	0.7	33	4,635,632
01-04 years	Female	Black	1980-1984	0.6	31	5,047,308
01-04 years	Female	Black	1985-1989	0.6	33	5,347,074
01-04 years	Female	Black	1990-1994	0.7	46	6,185,712
01-04 years	Female	Black	1995-1999	0.5	31	6,285,203
01-04 years	Female	Black	2000-2004	0.6	36	6,135,756
01-04 years	Female	Black	2005-2009	0.9	55	6,366,510
01-04 years	Female	Black	2010-2016	0.7	62	9,353,888
01-04 years	Female	Other	1975-1979	0.0	0	800,831
01-04 years	Female	Other	1980-1984	0.0	0	1,152,415
01-04 years	Female	Other	1985-1989	0.0	0	1,508,528
01-04 years	Female	Other	1990-1994	0.0	0	1,823,487
01-04 years	Female	Other	1995-1999	0.0	0	2,079,272
01-04 years	Female	Other	2000-2004	0.0	0	2,390,015
01-04 years	Female	Other	2005-2009	0.0	0	2,862,318
01-04 years	Female	Other	2010-2016	0.0	0	4,408,130
05-09 years	Male and female	All races	1975-1979	0.1	124	87,074,598
05-09 years	Male and female	All races	1980-1984	0.2	164	81,011,395
05-09 years	Male and female	All races	1985-1989	0.2	172	86,869,121
05-09 years	Male and female	All races	1990-1994	0.2	200	92,459,615
05-09 years	Male and female	All races	1995-1999	0.2	246	100,669,521
05-09 years	Male and female	All races	2000-2004	0.2	214	99,584,719
05-09 years	Male and female	All races	2005-2009	0.3	274	98,760,467
05-09 years	Male and female	All races	2010-2016	0.3	478	143,119,956
05-09 years	Male and female	White	1975-1979	0.1	87	72,399,906
05-09 years	Male and female	White	1980-1984	0.2	107	66,066,833
05-09 years	Male and female	White	1985-1989	0.1	87	70,103,875
05-09 years	Male and female	White	1990-1994	0.1	95	73,683,911
05-09 years	Male and female	White	1995-1999	0.2	120	78,505,936
05-09 years	Male and female	White	2000-2004	0.1	89	77,064,834
05-09 years	Male and female	White	2005-2009	0.2	122	75,840,713
05-09 years	Male and female	White	2010-2016	0.2	170	108,025,255

05-09 years	Male and female	Black	1975-1979	0.3	34	12,591,267
05-09 years	Male and female	Black	1980-1984	0.4	52	12,267,725
05-09 years	Male and female	Black	1985-1989	0.6	75	13,201,267
05-09 years	Male and female	Black	1990-1994	0.7	100	14,396,032
05-09 years	Male and female	Black	1995-1999	0.7	116	16,922,640
05-09 years	Male and female	Black	2000-2004	0.7	118	16,584,385
05-09 years	Male and female	Black	2005-2009	0.9	140	16,080,595
05-09 years	Male and female	Black	2010-2016	1.2	287	23,681,532
05-09 years	Male and female	Other	1975-1979	0.0	0	2,083,425
05-09 years	Male and female	Other	1980-1984	0.0	0	2,676,837
05-09 years	Male and female	Other	1985-1989	0.3	10	3,563,979
05-09 years	Male and female	Other	1990-1994	0.0	0	4,379,672
05-09 years	Male and female	Other	1995-1999	0.2	10	5,240,945
05-09 years	Male and female	Other	2000-2004	0.0	0	5,935,500
05-09 years	Male and female	Other	2005-2009	0.2	12	6,839,159
05-09 years	Male and female	Other	2010-2016	0.2	21	11,413,169
05-09 years	Male	All races	1975-1979	0.1	59	44,449,008
05-09 years	Male	All races	1980-1984	0.2	89	41,453,966
05-09 years	Male	All races	1985-1989	0.2	108	44,455,715
05-09 years	Male	All races	1990-1994	0.2	116	47,334,774
05-09 years	Male	All races	1995-1999	0.3	150	51,547,947
05-09 years	Male	All races	2000-2004	0.2	127	50,971,713
05-09 years	Male	All races	2005-2009	0.3	174	50,467,479
05-09 years	Male	All races	2010-2016	0.4	299	73,075,947
05-09 years	Male	White	1975-1979	0.1	41	37,074,812
05-09 years	Male	White	1980-1984	0.2	59	33,903,800
05-09 years	Male	White	1985-1989	0.1	53	35,978,330
05-09 years	Male	White	1990-1994	0.2	62	37,810,591
05-09 years	Male	White	1995-1999	0.2	68	40,273,350
05-09 years	Male	White	2000-2004	0.1	49	39,536,987
05-09 years	Male	White	2005-2009	0.2	78	38,858,924
05-09 years	Male	White	2010-2016	0.2	94	55,269,068
05-09 years	Male	Black	1975-1979	0.2	15	6,320,141
05-09 years	Male	Black	1980-1984	0.5	29	6,189,700
05-09 years	Male	Black	1985-1989	0.7	48	6,669,570
05-09 years	Male	Black	1990-1994	0.7	52	7,292,224
05-09 years	Male	Black	1995-1999	0.9	74	8,587,748
05-09 years	Male	Black	2000-2004	0.9	76	8,427,606
05-09 years	Male	Black	2005-2009	1.1	89	8,171,374
05-09 years	Male	Black	2010-2016	1.6	190	12,032,667
05-09 years	Male	Other	1975-1979	0.0	0	1,054,055
05-09 years	Male	Other	1980-1984	0.0	0	1,360,466
05-09 years	Male	Other	1985-1989	0.0	0	1,807,815

05-09 years	Male	Other	1990-1994	0.0	0	2,231,959
05-09 years	Male	Other	1995-1999	0.0	0	2,686,849
05-09 years	Male	Other	2000-2004	0.0	0	3,007,120
05-09 years	Male	Other	2005-2009	0.0	0	3,437,181
05-09 years	Male	Other	2010-2016	0.3	15	5,774,212
05-09 years	Female	All races	1975-1979	0.2	65	42,625,590
05-09 years	Female	All races	1980-1984	0.2	75	39,557,429
05-09 years	Female	All races	1985-1989	0.2	64	42,413,406
05-09 years	Female	All races	1990-1994	0.2	84	45,124,841
05-09 years	Female	All races	1995-1999	0.2	96	49,121,574
05-09 years	Female	All races	2000-2004	0.2	87	48,613,006
05-09 years	Female	All races	2005-2009	0.2	100	48,292,988
05-09 years	Female	All races	2010-2016	0.3	179	70,044,009
05-09 years	Female	White	1975-1979	0.1	46	35,325,094
05-09 years	Female	White	1980-1984	0.1	48	32,163,033
05-09 years	Female	White	1985-1989	0.1	34	34,125,545
05-09 years	Female	White	1990-1994	0.1	33	35,873,320
05-09 years	Female	White	1995-1999	0.1	52	38,232,586
05-09 years	Female	White	2000-2004	0.1	40	37,527,847
05-09 years	Female	White	2005-2009	0.1	44	36,981,789
05-09 years	Female	White	2010-2016	0.1	76	52,756,187
05-09 years	Female	Black	1975-1979	0.3	19	6,271,126
05-09 years	Female	Black	1980-1984	0.4	23	6,078,025
05-09 years	Female	Black	1985-1989	0.4	27	6,531,697
05-09 years	Female	Black	1990-1994	0.7	48	7,103,808
05-09 years	Female	Black	1995-1999	0.5	42	8,334,892
05-09 years	Female	Black	2000-2004	0.5	42	8,156,779
05-09 years	Female	Black	2005-2009	0.6	51	7,909,221
05-09 years	Female	Black	2010-2016	0.8	97	11,648,865
05-09 years	Female	Other	1975-1979	0.0	0	1,029,370
05-09 years	Female	Other	1980-1984	0.0	0	1,316,371
05-09 years	Female	Other	1985-1989	0.0	0	1,756,164
05-09 years	Female	Other	1990-1994	0.0	0	2,147,713
05-09 years	Female	Other	1995-1999	0.0	0	2,554,096
05-09 years	Female	Other	2000-2004	0.0	0	2,928,380
05-09 years	Female	Other	2005-2009	0.0	0	3,401,978
05-09 years	Female	Other	2010-2016	0.0	0	5,638,957
10-14 years	Male and female	All races	1975-1979	0.2	214	97,636,858
10-14 years	Male and female	All races	1980-1984	0.3	292	89,999,085
10-14 years	Male and female	All races	1985-1989	0.5	384	83,170,387
10-14 years	Male and female	All races	1990-1994	0.4	402	90,937,294
10-14 years	Male and female	All races	1995-1999	0.5	482	98,281,400
10-14 years	Male and female	All races	2000-2004	0.4	403	105,704,828

10-14 years	Male and female	All races	2005-2009	0.3	305	104,453,978
10-14 years	Male and female	All races	2010-2016	0.4	520	144,627,354
10-14 years	Male and female	White	1975-1979	0.2	156	81,580,676
10-14 years	Male and female	White	1980-1984	0.2	162	73,882,997
10-14 years	Male and female	White	1985-1989	0.3	205	67,010,198
10-14 years	Male and female	White	1990-1994	0.3	214	72,512,804
10-14 years	Male and female	White	1995-1999	0.3	232	77,606,043
10-14 years	Male and female	White	2000-2004	0.2	184	81,882,216
10-14 years	Male and female	White	2005-2009	0.1	103	80,024,746
10-14 years	Male and female	White	2010-2016	0.2	202	109,895,831
10-14 years	Male and female	Black	1975-1979	0.4	53	13,953,593
10-14 years	Male and female	Black	1980-1984	0.9	124	13,361,405
10-14 years	Male and female	Black	1985-1989	1.3	169	12,759,322
10-14 years	Male and female	Black	1990-1994	1.3	177	14,045,044
10-14 years	Male and female	Black	1995-1999	1.5	230	15,409,819
10-14 years	Male and female	Black	2000-2004	1.2	209	17,671,640
10-14 years	Male and female	Black	2005-2009	1.1	190	17,505,148
10-14 years	Male and female	Black	2010-2016	1.3	303	23,678,159
10-14 years	Male and female	Other	1975-1979	0.0	0	2,102,589
10-14 years	Male and female	Other	1980-1984	0.0	0	2,754,683
10-14 years	Male and female	Other	1985-1989	0.3	10	3,400,867
10-14 years	Male and female	Other	1990-1994	0.3	11	4,379,446
10-14 years	Male and female	Other	1995-1999	0.4	20	5,265,538
10-14 years	Male and female	Other	2000-2004	0.2	10	6,150,972
10-14 years	Male and female	Other	2005-2009	0.2	12	6,924,084
10-14 years	Male and female	Other	2010-2016	0.1	15	11,053,364
10-14 years	Male	All races	1975-1979	0.3	134	49,826,574
10-14 years	Male	All races	1980-1984	0.4	169	46,010,522
10-14 years	Male	All races	1985-1989	0.5	218	42,601,260
10-14 years	Male	All races	1990-1994	0.5	246	46,590,828
10-14 years	Male	All races	1995-1999	0.6	297	50,358,686
10-14 years	Male	All races	2000-2004	0.4	238	54,150,302
10-14 years	Male	All races	2005-2009	0.4	191	53,476,364
10-14 years	Male	All races	2010-2016	0.4	320	73,860,715
10-14 years	Male	White	1975-1979	0.2	98	41,737,149
10-14 years	Male	White	1980-1984	0.2	88	37,869,520
10-14 years	Male	White	1985-1989	0.3	108	34,416,093
10-14 years	Male	White	1990-1994	0.3	118	37,258,228
10-14 years	Male	White	1995-1999	0.4	141	39,855,465
10-14 years	Male	White	2000-2004	0.2	104	42,029,372
10-14 years	Male	White	2005-2009	0.2	64	41,059,281
10-14 years	Male	White	2010-2016	0.2	121	56,253,956
10-14 years	Male	Black	1975-1979	0.5	33	7,018,443

10-14 years	Male	Black	1980-1984	1.1	77	6,733,288
10-14 years	Male	Black	1985-1989	1.6	101	6,447,716
10-14 years	Male	Black	1990-1994	1.7	121	7,105,383
10-14 years	Male	Black	1995-1999	1.8	142	7,817,097
10-14 years	Male	Black	2000-2004	1.4	127	8,974,252
10-14 years	Male	Black	2005-2009	1.4	122	8,903,587
10-14 years	Male	Black	2010-2016	1.6	190	12,034,015
10-14 years	Male	Other	1975-1979	0.0	0	1,070,982
10-14 years	Male	Other	1980-1984	0.0	0	1,407,714
10-14 years	Male	Other	1985-1989	0.0	0	1,737,451
10-14 years	Male	Other	1990-1994	0.0	0	2,227,217
10-14 years	Male	Other	1995-1999	0.5	14	2,686,124
10-14 years	Male	Other	2000-2004	0.0	0	3,146,678
10-14 years	Male	Other	2005-2009	0.0	0	3,513,496
10-14 years	Male	Other	2010-2016	0.0	0	5,572,744
10-14 years	Female	All races	1975-1979	0.2	80	47,810,284
10-14 years	Female	All races	1980-1984	0.3	123	43,988,563
10-14 years	Female	All races	1985-1989	0.4	166	40,569,127
10-14 years	Female	All races	1990-1994	0.4	156	44,346,466
10-14 years	Female	All races	1995-1999	0.4	185	47,922,714
10-14 years	Female	All races	2000-2004	0.3	165	51,554,526
10-14 years	Female	All races	2005-2009	0.2	114	50,977,614
10-14 years	Female	All races	2010-2016	0.3	200	70,766,639
10-14 years	Female	White	1975-1979	0.1	58	39,843,527
10-14 years	Female	White	1980-1984	0.2	74	36,013,477
10-14 years	Female	White	1985-1989	0.3	97	32,594,105
10-14 years	Female	White	1990-1994	0.3	96	35,254,576
10-14 years	Female	White	1995-1999	0.2	91	37,750,578
10-14 years	Female	White	2000-2004	0.2	80	39,852,844
10-14 years	Female	White	2005-2009	0.1	39	38,965,465
10-14 years	Female	White	2010-2016	0.2	81	53,641,875
10-14 years	Female	Black	1975-1979	0.3	20	6,935,150
10-14 years	Female	Black	1980-1984	0.7	47	6,628,117
10-14 years	Female	Black	1985-1989	1.1	68	6,311,606
10-14 years	Female	Black	1990-1994	0.8	56	6,939,661
10-14 years	Female	Black	1995-1999	1.2	88	7,592,722
10-14 years	Female	Black	2000-2004	0.9	82	8,697,388
10-14 years	Female	Black	2005-2009	0.8	68	8,601,561
10-14 years	Female	Black	2010-2016	1.0	113	11,644,144
10-14 years	Female	Other	1975-1979	0.0	0	1,031,607
10-14 years	Female	Other	1980-1984	0.0	0	1,346,969
10-14 years	Female	Other	1985-1989	0.0	0	1,663,416
10-14 years	Female	Other	1990-1994	0.0	0	2,152,229

10-14 years	Female	Other	1995-1999	0.0	0	2,579,414
10-14 years	Female	Other	2000-2004	0.0	0	3,004,294
10-14 years	Female	Other	2005-2009	0.0	0	3,410,588
10-14 years	Female	Other	2010-2016	0.0	0	5,480,620
15-19 years	Male and female	All races	1975-1979	0.3	293	106,999,880
15-19 years	Male and female	All races	1980-1984	0.4	379	99,932,452
15-19 years	Male and female	All races	1985-1989	0.5	438	92,869,269
15-19 years	Male and female	All races	1990-1994	0.6	486	87,637,775
15-19 years	Male and female	All races	1995-1999	0.6	544	96,616,183
15-19 years	Male and female	All races	2000-2004	0.4	422	103,261,327
15-19 years	Male and female	All races	2005-2009	0.3	349	109,765,429
15-19 years	Male and female	All races	2010-2016	0.3	441	149,504,383
15-19 years	Male and female	White	1975-1979	0.2	199	90,193,835
15-19 years	Male and female	White	1980-1984	0.3	219	82,602,531
15-19 years	Male and female	White	1985-1989	0.3	253	75,502,429
15-19 years	Male and female	White	1990-1994	0.4	281	69,935,594
15-19 years	Male and female	White	1995-1999	0.4	307	76,434,331
15-19 years	Male and female	White	2000-2004	0.3	204	80,831,098
15-19 years	Male and female	White	2005-2009	0.2	171	83,998,067
15-19 years	Male and female	White	2010-2016	0.2	203	113,295,633
15-19 years	Male and female	Black	1975-1979	0.6	89	14,682,413
15-19 years	Male and female	Black	1980-1984	1.0	150	14,565,593
15-19 years	Male and female	Black	1985-1989	1.2	167	13,776,464
15-19 years	Male and female	Black	1990-1994	1.4	191	13,500,648
15-19 years	Male and female	Black	1995-1999	1.5	221	14,996,082
15-19 years	Male and female	Black	2000-2004	1.2	200	16,163,524
15-19 years	Male and female	Black	2005-2009	0.9	162	18,441,039
15-19 years	Male and female	Black	2010-2016	0.9	218	24,996,079
15-19 years	Male and female	Other	1975-1979	0.0	0	2,123,632
15-19 years	Male and female	Other	1980-1984	0.4	10	2,764,328
15-19 years	Male and female	Other	1985-1989	0.5	18	3,590,376
15-19 years	Male and female	Other	1990-1994	0.3	14	4,201,533
15-19 years	Male and female	Other	1995-1999	0.3	16	5,185,770
15-19 years	Male and female	Other	2000-2004	0.3	18	6,266,705
15-19 years	Male and female	Other	2005-2009	0.2	16	7,326,323
15-19 years	Male and female	Other	2010-2016	0.2	20	11,212,671
15-19 years	Male	All races	1975-1979	0.3	162	54,298,469
15-19 years	Male	All races	1980-1984	0.4	212	50,844,067
15-19 years	Male	All races	1985-1989	0.5	248	47,480,182
15-19 years	Male	All races	1990-1994	0.6	284	45,001,220
15-19 years	Male	All races	1995-1999	0.6	316	49,666,099
15-19 years	Male	All races	2000-2004	0.5	274	53,186,259
15-19 years	Male	All races	2005-2009	0.4	209	56,392,302

15-19 years	Male	All races	2010-2016	0.4	280	76,593,224
15-19 years	Male	White	1975-1979	0.2	110	45,898,389
15-19 years	Male	White	1980-1984	0.3	114	42,121,305
15-19 years	Male	White	1985-1989	0.4	137	38,685,279
15-19 years	Male	White	1990-1994	0.4	154	36,020,519
15-19 years	Male	White	1995-1999	0.4	173	39,436,479
15-19 years	Male	White	2000-2004	0.3	121	41,763,017
15-19 years	Male	White	2005-2009	0.2	98	43,264,904
15-19 years	Male	White	2010-2016	0.2	129	58,175,405
15-19 years	Male	Black	1975-1979	0.7	50	7,314,774
15-19 years	Male	Black	1980-1984	1.3	92	7,288,802
15-19 years	Male	Black	1985-1989	1.5	101	6,933,407
15-19 years	Male	Black	1990-1994	1.7	118	6,820,511
15-19 years	Male	Black	1995-1999	1.8	134	7,580,432
15-19 years	Male	Black	2000-2004	1.7	142	8,207,787
15-19 years	Male	Black	2005-2009	1.1	100	9,363,700
15-19 years	Male	Black	2010-2016	1.1	140	12,716,935
15-19 years	Male	Other	1975-1979	0.0	0	1,085,306
15-19 years	Male	Other	1980-1984	0.0	0	1,433,960
15-19 years	Male	Other	1985-1989	0.5	10	1,861,496
15-19 years	Male	Other	1990-1994	0.6	12	2,160,190
15-19 years	Male	Other	1995-1999	0.0	0	2,649,188
15-19 years	Male	Other	2000-2004	0.3	11	3,215,455
15-19 years	Male	Other	2005-2009	0.3	11	3,763,698
15-19 years	Male	Other	2010-2016	0.2	11	5,700,884
15-19 years	Female	All races	1975-1979	0.2	131	52,701,411
15-19 years	Female	All races	1980-1984	0.3	167	49,088,385
15-19 years	Female	All races	1985-1989	0.4	190	45,389,087
15-19 years	Female	All races	1990-1994	0.5	202	42,636,555
15-19 years	Female	All races	1995-1999	0.5	228	46,950,084
15-19 years	Female	All races	2000-2004	0.3	148	50,075,068
15-19 years	Female	All races	2005-2009	0.3	140	53,373,127
15-19 years	Female	All races	2010-2016	0.2	161	72,911,159
15-19 years	Female	White	1975-1979	0.2	89	44,295,446
15-19 years	Female	White	1980-1984	0.3	105	40,481,226
15-19 years	Female	White	1985-1989	0.3	116	36,817,150
15-19 years	Female	White	1990-1994	0.4	127	33,915,075
15-19 years	Female	White	1995-1999	0.4	134	36,997,852
15-19 years	Female	White	2000-2004	0.2	83	39,068,081
15-19 years	Female	White	2005-2009	0.2	73	40,733,163
15-19 years	Female	White	2010-2016	0.1	74	55,120,228
15-19 years	Female	Black	1975-1979	0.5	39	7,367,639
15-19 years	Female	Black	1980-1984	0.8	58	7,276,791

15-19 years	Female	Black	1985-1989	1.0	66	6,843,057
15-19 years	Female	Black	1990-1994	1.1	73	6,680,137
15-19 years	Female	Black	1995-1999	1.2	87	7,415,650
15-19 years	Female	Black	2000-2004	0.7	58	7,955,737
15-19 years	Female	Black	2005-2009	0.7	62	9,077,339
15-19 years	Female	Black	2010-2016	0.6	78	12,279,144
15-19 years	Female	Other	1975-1979	0.0	0	1,038,326
15-19 years	Female	Other	1980-1984	0.0	0	1,330,368
15-19 years	Female	Other	1985-1989	0.0	0	1,728,880
15-19 years	Female	Other	1990-1994	0.0	0	2,041,343
15-19 years	Female	Other	1995-1999	0.0	0	2,536,582
15-19 years	Female	Other	2000-2004	0.0	0	3,051,250
15-19 years	Female	Other	2005-2009	0.0	0	3,562,625
15-19 years	Female	Other	2010-2016	0.0	0	5,511,787
20-24 years	Male and female	All races	1975-1979	0.3	335	101,309,601
20-24 years	Male and female	All races	1980-1984	0.4	456	107,890,170
20-24 years	Male and female	All races	1985-1989	0.5	496	101,112,146
20-24 years	Male and female	All races	1990-1994	0.6	528	95,218,801
20-24 years	Male and female	All races	1995-1999	0.6	588	90,845,467
20-24 years	Male and female	All races	2000-2004	0.5	501	100,554,966
20-24 years	Male and female	All races	2005-2009	0.4	449	105,638,823
20-24 years	Male and female	All races	2010-2016	0.5	762	157,229,485
20-24 years	Male and female	White	1975-1979	0.3	229	86,573,613
20-24 years	Male and female	White	1980-1984	0.3	295	90,823,158
20-24 years	Male and female	White	1985-1989	0.3	288	83,428,189
20-24 years	Male and female	White	1990-1994	0.4	304	76,923,523
20-24 years	Male and female	White	1995-1999	0.5	333	71,979,046
20-24 years	Male and female	White	2000-2004	0.4	285	78,833,620
20-24 years	Male and female	White	2005-2009	0.3	220	81,791,364
20-24 years	Male and female	White	2010-2016	0.3	413	118,357,532
20-24 years	Male and female	Black	1975-1979	0.8	96	12,552,646
20-24 years	Male and female	Black	1980-1984	1.1	149	14,095,378
20-24 years	Male and female	Black	1985-1989	1.4	195	13,914,768
20-24 years	Male and female	Black	1990-1994	1.5	206	13,474,448
20-24 years	Male and female	Black	1995-1999	1.8	235	13,351,183
20-24 years	Male and female	Black	2000-2004	1.3	198	14,960,345
20-24 years	Male and female	Black	2005-2009	1.4	218	16,023,445
20-24 years	Male and female	Black	2010-2016	1.2	311	26,092,381
20-24 years	Male and female	Other	1975-1979	0.5	10	2,183,342
20-24 years	Male and female	Other	1980-1984	0.4	12	2,971,634
20-24 years	Male and female	Other	1985-1989	0.3	13	3,769,189
20-24 years	Male and female	Other	1990-1994	0.4	18	4,820,830
20-24 years	Male and female	Other	1995-1999	0.4	20	5,515,238

20-24 years	Male and female	Other	2000-2004	0.3	18	6,761,001
20-24 years	Male and female	Other	2005-2009	0.1	11	7,824,014
20-24 years	Male and female	Other	2010-2016	0.3	38	12,779,572
20-24 years	Male	All races	1975-1979	0.4	187	50,641,852
20-24 years	Male	All races	1980-1984	0.5	257	54,149,373
20-24 years	Male	All races	1985-1989	0.5	269	51,178,547
20-24 years	Male	All races	1990-1994	0.6	301	48,459,100
20-24 years	Male	All races	1995-1999	0.8	355	46,308,414
20-24 years	Male	All races	2000-2004	0.6	289	51,327,593
20-24 years	Male	All races	2005-2009	0.5	276	54,108,091
20-24 years	Male	All races	2010-2016	0.6	505	80,480,998
20-24 years	Male	White	1975-1979	0.3	126	43,563,342
20-24 years	Male	White	1980-1984	0.4	164	45,870,936
20-24 years	Male	White	1985-1989	0.3	143	42,454,559
20-24 years	Male	White	1990-1994	0.4	157	39,380,166
20-24 years	Male	White	1995-1999	0.5	187	36,992,098
20-24 years	Male	White	2000-2004	0.4	163	40,559,544
20-24 years	Male	White	2005-2009	0.3	134	42,150,031
20-24 years	Male	White	2010-2016	0.4	264	60,832,694
20-24 years	Male	Black	1975-1979	0.9	55	5,976,050
20-24 years	Male	Black	1980-1984	1.3	88	6,767,045
20-24 years	Male	Black	1985-1989	1.7	118	6,771,490
20-24 years	Male	Black	1990-1994	2.0	131	6,594,542
20-24 years	Male	Black	1995-1999	2.4	155	6,515,010
20-24 years	Male	Black	2000-2004	1.5	113	7,350,157
20-24 years	Male	Black	2005-2009	1.7	137	7,974,354
20-24 years	Male	Black	2010-2016	1.6	216	13,129,229
20-24 years	Male	Other	1975-1979	0.0	0	1,102,460
20-24 years	Male	Other	1980-1984	0.0	0	1,511,392
20-24 years	Male	Other	1985-1989	0.0	0	1,952,498
20-24 years	Male	Other	1990-1994	0.5	13	2,484,392
20-24 years	Male	Other	1995-1999	0.5	13	2,801,306
20-24 years	Male	Other	2000-2004	0.4	13	3,417,892
20-24 years	Male	Other	2005-2009	0.0	0	3,983,706
20-24 years	Male	Other	2010-2016	0.4	25	6,519,075
20-24 years	Female	All races	1975-1979	0.3	148	50,667,749
20-24 years	Female	All races	1980-1984	0.4	199	53,740,797
20-24 years	Female	All races	1985-1989	0.5	227	49,933,599
20-24 years	Female	All races	1990-1994	0.5	227	46,759,701
20-24 years	Female	All races	1995-1999	0.5	233	44,537,053
20-24 years	Female	All races	2000-2004	0.4	212	49,227,373
20-24 years	Female	All races	2005-2009	0.3	173	51,530,732
20-24 years	Female	All races	2010-2016	0.3	257	76,748,487

20-24 years	Female	White	1975-1979	0.2	103	43,010,271
20-24 years	Female	White	1980-1984	0.3	131	44,952,222
20-24 years	Female	White	1985-1989	0.4	145	40,973,630
20-24 years	Female	White	1990-1994	0.4	147	37,543,357
20-24 years	Female	White	1995-1999	0.4	146	34,986,948
20-24 years	Female	White	2000-2004	0.3	122	38,274,076
20-24 years	Female	White	2005-2009	0.2	86	39,641,333
20-24 years	Female	White	2010-2016	0.3	149	57,524,838
20-24 years	Female	Black	1975-1979	0.6	41	6,576,596
20-24 years	Female	Black	1980-1984	0.8	61	7,328,333
20-24 years	Female	Black	1985-1989	1.1	77	7,143,278
20-24 years	Female	Black	1990-1994	1.1	75	6,879,906
20-24 years	Female	Black	1995-1999	1.2	80	6,836,173
20-24 years	Female	Black	2000-2004	1.1	85	7,610,188
20-24 years	Female	Black	2005-2009	1.0	81	8,049,091
20-24 years	Female	Black	2010-2016	0.7	95	12,963,152
20-24 years	Female	Other	1975-1979	0.0	0	1,080,882
20-24 years	Female	Other	1980-1984	0.0	0	1,460,242
20-24 years	Female	Other	1985-1989	0.0	0	1,816,691
20-24 years	Female	Other	1990-1994	0.0	0	2,336,438
20-24 years	Female	Other	1995-1999	0.0	0	2,713,932
20-24 years	Female	Other	2000-2004	0.0	0	3,343,109
20-24 years	Female	Other	2005-2009	0.0	0	3,840,308
20-24 years	Female	Other	2010-2016	0.2	13	6,260,497
25-29 years	Male and female	All races	1975-1979	0.4	386	91,244,201
25-29 years	Male and female	All races	1980-1984	0.5	506	103,156,673
25-29 years	Male and female	All races	1985-1989	0.5	587	108,718,504
25-29 years	Male and female	All races	1990-1994	0.6	659	102,511,259
25-29 years	Male and female	All races	1995-1999	0.8	759	98,821,817
25-29 years	Male and female	All races	2000-2004	0.6	573	94,669,590
25-29 years	Male and female	All races	2005-2009	0.6	583	102,168,315
25-29 years	Male and female	All races	2010-2016	0.6	915	152,732,128
25-29 years	Male and female	White	1975-1979	0.3	241	78,834,898
25-29 years	Male and female	White	1980-1984	0.4	310	87,403,224
25-29 years	Male and female	White	1985-1989	0.4	358	90,816,978
25-29 years	Male and female	White	1990-1994	0.4	369	83,853,532
25-29 years	Male and female	White	1995-1999	0.5	423	79,243,792
25-29 years	Male and female	White	2000-2004	0.4	320	74,277,516
25-29 years	Male and female	White	2005-2009	0.4	342	79,177,231
25-29 years	Male and female	White	2010-2016	0.5	533	116,143,829
25-29 years	Male and female	Black	1975-1979	1.3	137	10,268,609
25-29 years	Male and female	Black	1980-1984	1.5	185	12,642,621
25-29 years	Male and female	Black	1985-1989	1.5	210	13,853,993

25-29 years	Male and female	Black	1990-1994	1.9	266	13,703,017
25-29 years	Male and female	Black	1995-1999	2.3	312	13,513,225
25-29 years	Male and female	Black	2000-2004	1.8	233	13,221,510
25-29 years	Male and female	Black	2005-2009	1.5	223	14,604,130
25-29 years	Male and female	Black	2010-2016	1.5	346	22,960,445
25-29 years	Male and female	Other	1975-1979	0.0	0	2,140,694
25-29 years	Male and female	Other	1980-1984	0.4	11	3,110,828
25-29 years	Male and female	Other	1985-1989	0.5	19	4,047,533
25-29 years	Male and female	Other	1990-1994	0.5	24	4,954,710
25-29 years	Male and female	Other	1995-1999	0.4	24	6,064,800
25-29 years	Male and female	Other	2000-2004	0.3	20	7,170,564
25-29 years	Male and female	Other	2005-2009	0.2	18	8,386,954
25-29 years	Male and female	Other	2010-2016	0.3	36	13,627,854
25-29 years	Male	All races	1975-1979	0.4	201	45,294,267
25-29 years	Male	All races	1980-1984	0.5	268	51,466,212
25-29 years	Male	All races	1985-1989	0.6	311	54,503,468
25-29 years	Male	All races	1990-1994	0.7	352	51,534,406
25-29 years	Male	All races	1995-1999	0.8	385	49,781,993
25-29 years	Male	All races	2000-2004	0.7	327	47,799,334
25-29 years	Male	All races	2005-2009	0.7	338	51,351,687
25-29 years	Male	All races	2010-2016	0.7	554	77,393,188
25-29 years	Male	White	1975-1979	0.3	125	39,520,315
25-29 years	Male	White	1980-1984	0.4	170	44,033,159
25-29 years	Male	White	1985-1989	0.4	172	45,945,879
25-29 years	Male	White	1990-1994	0.4	185	42,564,087
25-29 years	Male	White	1995-1999	0.5	201	40,393,695
25-29 years	Male	White	2000-2004	0.4	165	38,005,358
25-29 years	Male	White	2005-2009	0.5	191	40,324,125
25-29 years	Male	White	2010-2016	0.5	310	59,431,133
25-29 years	Male	Black	1975-1979	1.5	72	4,756,484
25-29 years	Male	Black	1980-1984	1.5	92	5,945,625
25-29 years	Male	Black	1985-1989	1.9	127	6,572,109
25-29 years	Male	Black	1990-1994	2.4	156	6,524,666
25-29 years	Male	Black	1995-1999	2.6	167	6,422,115
25-29 years	Male	Black	2000-2004	2.4	148	6,260,999
25-29 years	Male	Black	2005-2009	1.9	133	6,952,778
25-29 years	Male	Black	2010-2016	2.0	224	11,234,868
25-29 years	Male	Other	1975-1979	0.0	0	1,017,468
25-29 years	Male	Other	1980-1984	0.0	0	1,487,428
25-29 years	Male	Other	1985-1989	0.6	12	1,985,480
25-29 years	Male	Other	1990-1994	0.4	11	2,445,653
25-29 years	Male	Other	1995-1999	0.6	17	2,966,183
25-29 years	Male	Other	2000-2004	0.4	14	3,532,977

25-29 years	Male	Other	2005-2009	0.3	14	4,074,784
25-29 years	Male	Other	2010-2016	0.3	20	6,727,187
25-29 years	Female	All races	1975-1979	0.4	185	45,949,934
25-29 years	Female	All races	1980-1984	0.5	238	51,690,461
25-29 years	Female	All races	1985-1989	0.5	276	54,215,036
25-29 years	Female	All races	1990-1994	0.6	307	50,976,853
25-29 years	Female	All races	1995-1999	0.8	374	49,039,824
25-29 years	Female	All races	2000-2004	0.5	246	46,870,256
25-29 years	Female	All races	2005-2009	0.5	245	50,816,628
25-29 years	Female	All races	2010-2016	0.5	361	75,338,940
25-29 years	Female	White	1975-1979	0.3	116	39,314,583
25-29 years	Female	White	1980-1984	0.3	140	43,370,065
25-29 years	Female	White	1985-1989	0.4	186	44,871,099
25-29 years	Female	White	1990-1994	0.4	184	41,289,445
25-29 years	Female	White	1995-1999	0.6	222	38,850,097
25-29 years	Female	White	2000-2004	0.4	155	36,272,158
25-29 years	Female	White	2005-2009	0.4	151	38,853,106
25-29 years	Female	White	2010-2016	0.4	223	56,712,696
25-29 years	Female	Black	1975-1979	1.2	65	5,512,125
25-29 years	Female	Black	1980-1984	1.4	93	6,696,996
25-29 years	Female	Black	1985-1989	1.1	83	7,281,884
25-29 years	Female	Black	1990-1994	1.5	110	7,178,351
25-29 years	Female	Black	1995-1999	2.0	145	7,091,110
25-29 years	Female	Black	2000-2004	1.2	85	6,960,511
25-29 years	Female	Black	2005-2009	1.2	90	7,651,352
25-29 years	Female	Black	2010-2016	1.0	122	11,725,577
25-29 years	Female	Other	1975-1979	0.0	0	1,123,226
25-29 years	Female	Other	1980-1984	0.0	0	1,623,400
25-29 years	Female	Other	1985-1989	0.0	0	2,062,053
25-29 years	Female	Other	1990-1994	0.5	13	2,509,057
25-29 years	Female	Other	1995-1999	0.0	0	3,098,617
25-29 years	Female	Other	2000-2004	0.0	0	3,637,587
25-29 years	Female	Other	2005-2009	0.0	0	4,312,170
25-29 years	Female	Other	2010-2016	0.2	16	6,900,667
30-34 years	Male and female	All races	1975-1979	0.6	449	77,429,639
30-34 years	Male and female	All races	1980-1984	0.6	595	93,757,456
30-34 years	Male and female	All races	1985-1989	0.7	775	104,554,219
30-34 years	Male and female	All races	1990-1994	0.8	910	111,907,483
30-34 years	Male and female	All races	1995-1999	0.9	981	107,319,492
30-34 years	Male and female	All races	2000-2004	0.8	851	102,466,426
30-34 years	Male and female	All races	2005-2009	0.7	728	97,129,789
30-34 years	Male and female	All races	2010-2016	0.8	1,213	147,817,282
30-34 years	Male and female	White	1975-1979	0.4	299	67,451,385

30-34 years	Male and female	White	1980-1984	0.4	354	80,018,859
30-34 years	Male and female	White	1985-1989	0.5	456	87,764,967
30-34 years	Male and female	White	1990-1994	0.6	545	92,735,161
30-34 years	Male and female	White	1995-1999	0.6	555	87,267,549
30-34 years	Male and female	White	2000-2004	0.6	510	81,400,742
30-34 years	Male and female	White	2005-2009	0.6	422	75,378,502
30-34 years	Male and female	White	2010-2016	0.7	737	113,083,611
30-34 years	Male and female	Black	1975-1979	1.8	142	8,089,731
30-34 years	Male and female	Black	1980-1984	2.1	223	10,651,543
30-34 years	Male and female	Black	1985-1989	2.4	303	12,665,868
30-34 years	Male and female	Black	1990-1994	2.4	340	14,071,812
30-34 years	Male and female	Black	1995-1999	2.7	388	14,181,501
30-34 years	Male and female	Black	2000-2004	2.3	321	13,828,091
30-34 years	Male and female	Black	2005-2009	2.1	284	13,486,553
30-34 years	Male and female	Black	2010-2016	2.0	422	21,201,341
30-34 years	Male and female	Other	1975-1979	0.0	0	1,888,523
30-34 years	Male and female	Other	1980-1984	0.6	18	3,087,054
30-34 years	Male and female	Other	1985-1989	0.4	16	4,123,384
30-34 years	Male and female	Other	1990-1994	0.5	25	5,100,510
30-34 years	Male and female	Other	1995-1999	0.6	38	5,870,442
30-34 years	Male and female	Other	2000-2004	0.3	20	7,237,593
30-34 years	Male and female	Other	2005-2009	0.3	22	8,264,734
30-34 years	Male and female	Other	2010-2016	0.4	54	13,532,330
30-34 years	Male	All races	1975-1979	0.6	236	38,211,373
30-34 years	Male	All races	1980-1984	0.6	296	46,431,118
30-34 years	Male	All races	1985-1989	0.8	391	51,995,068
30-34 years	Male	All races	1990-1994	0.8	459	55,915,673
30-34 years	Male	All races	1995-1999	0.9	484	53,843,625
30-34 years	Male	All races	2000-2004	0.9	456	51,502,147
30-34 years	Male	All races	2005-2009	0.7	357	48,696,945
30-34 years	Male	All races	2010-2016	0.9	672	74,177,426
30-34 years	Male	White	1975-1979	0.5	164	33,615,715
30-34 years	Male	White	1980-1984	0.4	175	40,044,999
30-34 years	Male	White	1985-1989	0.5	221	44,105,704
30-34 years	Male	White	1990-1994	0.6	274	46,831,848
30-34 years	Male	White	1995-1999	0.6	265	44,313,378
30-34 years	Male	White	2000-2004	0.6	255	41,436,708
30-34 years	Male	White	2005-2009	0.5	186	38,355,781
30-34 years	Male	White	2010-2016	0.7	384	57,529,271
30-34 years	Male	Black	1975-1979	1.8	68	3,710,198
30-34 years	Male	Black	1980-1984	2.3	114	4,925,645
30-34 years	Male	Black	1985-1989	2.7	161	5,901,744
30-34 years	Male	Black	1990-1994	2.7	175	6,596,742

30-34 years	Male	Black	1995-1999	3.0	202	6,688,671
30-34 years	Male	Black	2000-2004	2.9	190	6,521,567
30-34 years	Male	Black	2005-2009	2.5	156	6,348,864
30-34 years	Male	Black	2010-2016	2.5	254	10,138,545
30-34 years	Male	Other	1975-1979	0.0	0	885,460
30-34 years	Male	Other	1980-1984	0.0	0	1,460,474
30-34 years	Male	Other	1985-1989	0.0	0	1,987,620
30-34 years	Male	Other	1990-1994	0.4	10	2,487,083
30-34 years	Male	Other	1995-1999	0.6	17	2,841,576
30-34 years	Male	Other	2000-2004	0.3	11	3,543,872
30-34 years	Male	Other	2005-2009	0.4	15	3,992,300
30-34 years	Male	Other	2010-2016	0.5	34	6,509,610
30-34 years	Female	All races	1975-1979	0.5	213	39,218,266
30-34 years	Female	All races	1980-1984	0.6	299	47,326,338
30-34 years	Female	All races	1985-1989	0.7	384	52,559,151
30-34 years	Female	All races	1990-1994	0.8	451	55,991,810
30-34 years	Female	All races	1995-1999	0.9	497	53,475,867
30-34 years	Female	All races	2000-2004	0.8	395	50,964,279
30-34 years	Female	All races	2005-2009	0.8	371	48,432,844
30-34 years	Female	All races	2010-2016	0.7	541	73,639,856
30-34 years	Female	White	1975-1979	0.4	135	33,835,670
30-34 years	Female	White	1980-1984	0.4	179	39,973,860
30-34 years	Female	White	1985-1989	0.5	235	43,659,263
30-34 years	Female	White	1990-1994	0.6	271	45,903,313
30-34 years	Female	White	1995-1999	0.7	290	42,954,171
30-34 years	Female	White	2000-2004	0.6	255	39,964,034
30-34 years	Female	White	2005-2009	0.6	236	37,022,721
30-34 years	Female	White	2010-2016	0.6	353	55,554,340
30-34 years	Female	Black	1975-1979	1.7	74	4,379,533
30-34 years	Female	Black	1980-1984	1.9	109	5,725,898
30-34 years	Female	Black	1985-1989	2.1	142	6,764,124
30-34 years	Female	Black	1990-1994	2.2	165	7,475,070
30-34 years	Female	Black	1995-1999	2.5	186	7,492,830
30-34 years	Female	Black	2000-2004	1.8	131	7,306,524
30-34 years	Female	Black	2005-2009	1.8	128	7,137,689
30-34 years	Female	Black	2010-2016	1.5	168	11,062,796
30-34 years	Female	Other	1975-1979	0.0	0	1,003,063
30-34 years	Female	Other	1980-1984	0.7	11	1,626,580
30-34 years	Female	Other	1985-1989	0.0	0	2,135,764
30-34 years	Female	Other	1990-1994	0.6	15	2,613,427
30-34 years	Female	Other	1995-1999	0.7	21	3,028,866
30-34 years	Female	Other	2000-2004	0.0	0	3,693,721
30-34 years	Female	Other	2005-2009	0.0	0	4,272,434

30-34 years	Female	Other	2010-2016	0.3	20	7,022,720
35-39 years	Male and female	All races	1975-1979	1.1	708	62,444,763
35-39 years	Male and female	All races	1980-1984	1.1	841	76,993,823
35-39 years	Male and female	All races	1985-1989	1.2	1,127	93,280,475
35-39 years	Male and female	All races	1990-1994	1.3	1,328	105,613,244
35-39 years	Male and female	All races	1995-1999	1.4	1,573	113,989,738
35-39 years	Male and female	All races	2000-2004	1.5	1,583	108,776,796
35-39 years	Male and female	All races	2005-2009	1.2	1,204	104,029,036
35-39 years	Male and female	All races	2010-2016	1.2	1,704	139,890,361
35-39 years	Male and female	White	1975-1979	0.9	480	54,428,589
35-39 years	Male and female	White	1980-1984	0.8	539	66,577,135
35-39 years	Male and female	White	1985-1989	0.9	707	79,098,187
35-39 years	Male and female	White	1990-1994	0.9	819	88,044,941
35-39 years	Male and female	White	1995-1999	1.1	990	93,761,011
35-39 years	Male and female	White	2000-2004	1.2	1,031	87,631,231
35-39 years	Male and female	White	2005-2009	1.0	794	81,925,609
35-39 years	Male and female	White	2010-2016	1.0	1,092	107,323,037
35-39 years	Male and female	Black	1975-1979	3.3	217	6,541,672
35-39 years	Male and female	Black	1980-1984	3.5	283	8,042,333
35-39 years	Male and female	Black	1985-1989	3.7	395	10,587,774
35-39 years	Male and female	Black	1990-1994	3.7	472	12,864,499
35-39 years	Male and female	Black	1995-1999	3.7	541	14,467,147
35-39 years	Male and female	Black	2000-2004	3.5	510	14,417,480
35-39 years	Male and female	Black	2005-2009	2.6	372	14,054,835
35-39 years	Male and female	Black	2010-2016	2.8	554	19,694,660
35-39 years	Male and female	Other	1975-1979	0.7	11	1,474,502
35-39 years	Male and female	Other	1980-1984	0.8	19	2,374,355
35-39 years	Male and female	Other	1985-1989	0.7	25	3,594,514
35-39 years	Male and female	Other	1990-1994	0.8	37	4,703,804
35-39 years	Male and female	Other	1995-1999	0.7	42	5,761,580
35-39 years	Male and female	Other	2000-2004	0.6	42	6,728,085
35-39 years	Male and female	Other	2005-2009	0.5	38	8,048,592
35-39 years	Male and female	Other	2010-2016	0.5	58	12,872,664
35-39 years	Male	All races	1975-1979	1.2	357	30,575,504
35-39 years	Male	All races	1980-1984	1.1	420	37,877,784
35-39 years	Male	All races	1985-1989	1.3	592	46,087,233
35-39 years	Male	All races	1990-1994	1.3	662	52,463,124
35-39 years	Male	All races	1995-1999	1.3	761	56,800,105
35-39 years	Male	All races	2000-2004	1.4	749	54,260,668
35-39 years	Male	All races	2005-2009	1.1	591	51,895,151
35-39 years	Male	All races	2010-2016	1.2	856	69,738,592
35-39 years	Male	White	1975-1979	0.9	229	26,927,531
35-39 years	Male	White	1980-1984	0.8	266	33,086,487

35-39 years	Male	White	1985-1989	0.9	362	39,511,663
35-39 years	Male	White	1990-1994	1.0	426	44,209,486
35-39 years	Male	White	1995-1999	1.0	473	47,223,820
35-39 years	Male	White	2000-2004	1.1	477	44,218,585
35-39 years	Male	White	2005-2009	0.9	369	41,421,290
35-39 years	Male	White	2010-2016	1.0	528	54,289,335
35-39 years	Male	Black	1975-1979	4.2	123	2,947,165
35-39 years	Male	Black	1980-1984	4.0	146	3,665,174
35-39 years	Male	Black	1985-1989	4.4	216	4,879,787
35-39 years	Male	Black	1990-1994	3.7	220	5,997,080
35-39 years	Male	Black	1995-1999	3.9	262	6,786,216
35-39 years	Male	Black	2000-2004	3.7	251	6,776,755
35-39 years	Male	Black	2005-2009	3.0	200	6,607,778
35-39 years	Male	Black	2010-2016	3.1	292	9,299,080
35-39 years	Male	Other	1975-1979	0.0	0	700,808
35-39 years	Male	Other	1980-1984	0.0	0	1,126,123
35-39 years	Male	Other	1985-1989	0.8	14	1,695,783
35-39 years	Male	Other	1990-1994	0.7	16	2,256,558
35-39 years	Male	Other	1995-1999	0.9	26	2,790,069
35-39 years	Male	Other	2000-2004	0.6	21	3,265,328
35-39 years	Male	Other	2005-2009	0.6	22	3,866,083
35-39 years	Male	Other	2010-2016	0.6	36	6,150,177
35-39 years	Female	All races	1975-1979	1.1	351	31,869,259
35-39 years	Female	All races	1980-1984	1.1	421	39,116,039
35-39 years	Female	All races	1985-1989	1.1	535	47,193,242
35-39 years	Female	All races	1990-1994	1.3	666	53,150,120
35-39 years	Female	All races	1995-1999	1.4	812	57,189,633
35-39 years	Female	All races	2000-2004	1.5	834	54,516,128
35-39 years	Female	All races	2005-2009	1.2	613	52,133,885
35-39 years	Female	All races	2010-2016	1.2	848	70,151,769
35-39 years	Female	White	1975-1979	0.9	251	27,501,058
35-39 years	Female	White	1980-1984	0.8	273	33,490,648
35-39 years	Female	White	1985-1989	0.9	345	39,586,524
35-39 years	Female	White	1990-1994	0.9	393	43,835,455
35-39 years	Female	White	1995-1999	1.1	517	46,537,191
35-39 years	Female	White	2000-2004	1.3	554	43,412,646
35-39 years	Female	White	2005-2009	1.0	425	40,504,319
35-39 years	Female	White	2010-2016	1.1	564	53,033,702
35-39 years	Female	Black	1975-1979	2.6	94	3,594,507
35-39 years	Female	Black	1980-1984	3.1	137	4,377,159
35-39 years	Female	Black	1985-1989	3.1	179	5,707,987
35-39 years	Female	Black	1990-1994	3.7	252	6,867,419
35-39 years	Female	Black	1995-1999	3.6	279	7,680,931

35-39 years	Female	Black	2000-2004	3.4	259	7,640,725
35-39 years	Female	Black	2005-2009	2.3	172	7,447,057
35-39 years	Female	Black	2010-2016	2.5	262	10,395,580
35-39 years	Female	Other	1975-1979	0.0	0	773,694
35-39 years	Female	Other	1980-1984	0.9	11	1,248,232
35-39 years	Female	Other	1985-1989	0.6	11	1,898,731
35-39 years	Female	Other	1990-1994	0.9	21	2,447,246
35-39 years	Female	Other	1995-1999	0.5	16	2,971,511
35-39 years	Female	Other	2000-2004	0.6	21	3,462,757
35-39 years	Female	Other	2005-2009	0.4	16	4,182,509
35-39 years	Female	Other	2010-2016	0.3	22	6,722,487
40-44 years	Male and female	All races	1975-1979	2.6	1,461	56,377,794
40-44 years	Male and female	All races	1980-1984	2.3	1,435	63,007,401
40-44 years	Male and female	All races	1985-1989	2.3	1,804	77,240,556
40-44 years	Male and female	All races	1990-1994	2.3	2,138	94,206,209
40-44 years	Male and female	All races	1995-1999	2.6	2,773	106,325,739
40-44 years	Male and female	All races	2000-2004	2.8	3,161	114,038,929
40-44 years	Male and female	All races	2005-2009	2.6	2,878	109,196,830
40-44 years	Male and female	All races	2010-2016	2.3	3,390	144,346,186
40-44 years	Male and female	White	1975-1979	2.3	1,118	49,089,711
40-44 years	Male and female	White	1980-1984	1.9	1,017	54,421,676
40-44 years	Male and female	White	1985-1989	1.9	1,274	66,405,513
40-44 years	Male and female	White	1990-1994	1.8	1,429	79,478,971
40-44 years	Male and female	White	1995-1999	2.1	1,895	88,196,608
40-44 years	Male and female	White	2000-2004	2.4	2,273	93,188,250
40-44 years	Male and female	White	2005-2009	2.5	2,144	87,342,598
40-44 years	Male and female	White	2010-2016	2.2	2,514	112,292,557
40-44 years	Male and female	Black	1975-1979	5.4	327	6,039,866
40-44 years	Male and female	Black	1980-1984	5.8	391	6,734,618
40-44 years	Male and female	Black	1985-1989	6.2	495	8,018,709
40-44 years	Male and female	Black	1990-1994	6.2	657	10,652,050
40-44 years	Male and female	Black	1995-1999	6.2	801	12,915,423
40-44 years	Male and female	Black	2000-2004	5.6	814	14,507,767
40-44 years	Male and female	Black	2005-2009	4.5	651	14,529,357
40-44 years	Male and female	Black	2010-2016	4.0	788	19,720,573
40-44 years	Male and female	Other	1975-1979	1.3	16	1,248,217
40-44 years	Male and female	Other	1980-1984	1.5	27	1,851,107
40-44 years	Male and female	Other	1985-1989	1.2	35	2,816,334
40-44 years	Male and female	Other	1990-1994	1.3	52	4,075,188
40-44 years	Male and female	Other	1995-1999	1.5	77	5,213,708
40-44 years	Male and female	Other	2000-2004	1.2	74	6,342,912
40-44 years	Male and female	Other	2005-2009	1.1	83	7,324,875
40-44 years	Male and female	Other	2010-2016	0.7	88	12,333,056

40-44 years	Male	All races	1975-1979	2.8	765	27,600,854
40-44 years	Male	All races	1980-1984	2.4	726	30,876,426
40-44 years	Male	All races	1985-1989	2.5	951	37,997,931
40-44 years	Male	All races	1990-1994	2.4	1,102	46,507,613
40-44 years	Male	All races	1995-1999	2.4	1,260	52,604,399
40-44 years	Male	All races	2000-2004	2.7	1,500	56,541,429
40-44 years	Male	All races	2005-2009	2.5	1,367	54,198,416
40-44 years	Male	All races	2010-2016	2.2	1,581	71,699,515
40-44 years	Male	White	1975-1979	2.4	581	24,267,382
40-44 years	Male	White	1980-1984	1.9	507	26,913,990
40-44 years	Male	White	1985-1989	2.0	648	32,983,374
40-44 years	Male	White	1990-1994	1.9	734	39,670,749
40-44 years	Male	White	1995-1999	2.0	871	44,116,979
40-44 years	Male	White	2000-2004	2.3	1,084	46,699,271
40-44 years	Male	White	2005-2009	2.3	1,005	43,853,026
40-44 years	Male	White	2010-2016	2.0	1,134	56,542,596
40-44 years	Male	Black	1975-1979	6.4	174	2,732,959
40-44 years	Male	Black	1980-1984	6.8	207	3,062,147
40-44 years	Male	Black	1985-1989	7.7	283	3,675,757
40-44 years	Male	Black	1990-1994	6.9	342	4,921,396
40-44 years	Male	Black	1995-1999	5.8	350	6,014,458
40-44 years	Male	Black	2000-2004	5.6	378	6,806,286
40-44 years	Male	Black	2005-2009	4.6	317	6,836,366
40-44 years	Male	Black	2010-2016	4.4	407	9,281,924
40-44 years	Male	Other	1975-1979	1.7	10	600,513
40-44 years	Male	Other	1980-1984	1.3	12	900,289
40-44 years	Male	Other	1985-1989	1.5	20	1,338,800
40-44 years	Male	Other	1990-1994	1.4	26	1,915,468
40-44 years	Male	Other	1995-1999	1.6	39	2,472,962
40-44 years	Male	Other	2000-2004	1.3	38	3,035,872
40-44 years	Male	Other	2005-2009	1.3	45	3,509,024
40-44 years	Male	Other	2010-2016	0.7	40	5,874,995
40-44 years	Female	All races	1975-1979	2.4	696	28,776,940
40-44 years	Female	All races	1980-1984	2.2	709	32,130,975
40-44 years	Female	All races	1985-1989	2.2	853	39,242,625
40-44 years	Female	All races	1990-1994	2.2	1,036	47,698,596
40-44 years	Female	All races	1995-1999	2.8	1,513	53,721,340
40-44 years	Female	All races	2000-2004	2.9	1,661	57,497,500
40-44 years	Female	All races	2005-2009	2.7	1,511	54,998,414
40-44 years	Female	All races	2010-2016	2.5	1,809	72,646,671
40-44 years	Female	White	1975-1979	2.2	537	24,822,329
40-44 years	Female	White	1980-1984	1.9	510	27,507,686
40-44 years	Female	White	1985-1989	1.9	626	33,422,139

40-44 years	Female	White	1990-1994	1.7	695	39,808,222
40-44 years	Female	White	1995-1999	2.3	1,024	44,079,629
40-44 years	Female	White	2000-2004	2.6	1,189	46,488,979
40-44 years	Female	White	2005-2009	2.6	1,139	43,489,572
40-44 years	Female	White	2010-2016	2.5	1,380	55,749,961
40-44 years	Female	Black	1975-1979	4.6	153	3,306,907
40-44 years	Female	Black	1980-1984	5.0	184	3,672,471
40-44 years	Female	Black	1985-1989	4.9	212	4,342,952
40-44 years	Female	Black	1990-1994	5.5	315	5,730,654
40-44 years	Female	Black	1995-1999	6.5	451	6,900,965
40-44 years	Female	Black	2000-2004	5.7	436	7,701,481
40-44 years	Female	Black	2005-2009	4.3	334	7,692,991
40-44 years	Female	Black	2010-2016	3.6	381	10,438,649
40-44 years	Female	Other	1975-1979	0.0	0	647,704
40-44 years	Female	Other	1980-1984	1.6	15	950,818
40-44 years	Female	Other	1985-1989	1.0	15	1,477,534
40-44 years	Female	Other	1990-1994	1.2	26	2,159,720
40-44 years	Female	Other	1995-1999	1.4	38	2,740,746
40-44 years	Female	Other	2000-2004	1.1	36	3,307,040
40-44 years	Female	Other	2005-2009	1.0	38	3,815,851
40-44 years	Female	Other	2010-2016	0.7	48	6,458,061
45-49 years	Male and female	All races	1975-1979	6.1	3,506	57,503,988
45-49 years	Male and female	All races	1980-1984	5.8	3,207	55,674,387
45-49 years	Male and female	All races	1985-1989	5.6	3,451	62,152,711
45-49 years	Male and female	All races	1990-1994	5.3	4,018	76,174,700
45-49 years	Male and female	All races	1995-1999	5.2	4,889	93,689,277
45-49 years	Male and female	All races	2000-2004	5.7	6,035	105,958,736
45-49 years	Male and female	All races	2005-2009	6.2	7,060	113,615,436
45-49 years	Male and female	All races	2010-2016	5.7	8,576	150,384,506
45-49 years	Male and female	White	1975-1979	5.6	2,860	50,707,936
45-49 years	Male and female	White	1980-1984	5.3	2,555	48,324,721
45-49 years	Male and female	White	1985-1989	5.0	2,694	53,472,118
45-49 years	Male and female	White	1990-1994	4.6	3,018	65,315,143
45-49 years	Male and female	White	1995-1999	4.6	3,616	78,789,125
45-49 years	Male and female	White	2000-2004	5.4	4,692	87,508,407
45-49 years	Male and female	White	2005-2009	6.1	5,656	92,314,349
45-49 years	Male and female	White	2010-2016	5.8	6,941	119,186,992
45-49 years	Male and female	Black	1975-1979	10.7	616	5,736,920
45-49 years	Male and female	Black	1980-1984	10.5	620	5,896,802
45-49 years	Male and female	Black	1985-1989	10.6	701	6,600,881
45-49 years	Male and female	Black	1990-1994	11.9	935	7,824,438
45-49 years	Male and female	Black	1995-1999	11.1	1,167	10,501,527
45-49 years	Male and female	Black	2000-2004	9.5	1,215	12,777,911

45-49 years	Male and female	Black	2005-2009	8.7	1,259	14,459,057
45-49 years	Male and female	Black	2010-2016	7.2	1,451	20,066,688
45-49 years	Male and female	Other	1975-1979	2.8	30	1,059,132
45-49 years	Male and female	Other	1980-1984	2.2	32	1,452,864
45-49 years	Male and female	Other	1985-1989	2.7	56	2,079,712
45-49 years	Male and female	Other	1990-1994	2.1	65	3,035,119
45-49 years	Male and female	Other	1995-1999	2.4	106	4,398,625
45-49 years	Male and female	Other	2000-2004	2.3	128	5,672,418
45-49 years	Male and female	Other	2005-2009	2.1	145	6,842,030
45-49 years	Male and female	Other	2010-2016	1.7	184	11,130,826
45-49 years	Male	All races	1975-1979	7.5	2,085	27,911,520
45-49 years	Male	All races	1980-1984	6.3	1,701	27,137,341
45-49 years	Male	All races	1985-1989	5.7	1,740	30,424,842
45-49 years	Male	All races	1990-1994	5.5	2,071	37,387,779
45-49 years	Male	All races	1995-1999	5.3	2,457	46,044,576
45-49 years	Male	All races	2000-2004	6.0	3,119	52,199,091
45-49 years	Male	All races	2005-2009	6.0	3,366	56,077,123
45-49 years	Male	All races	2010-2016	5.1	3,826	74,394,817
45-49 years	Male	White	1975-1979	6.8	1,676	24,816,638
45-49 years	Male	White	1980-1984	5.6	1,322	23,771,213
45-49 years	Male	White	1985-1989	5.1	1,341	26,408,298
45-49 years	Male	White	1990-1994	4.9	1,573	32,367,143
45-49 years	Male	White	1995-1999	4.7	1,826	39,148,365
45-49 years	Male	White	2000-2004	5.7	2,467	43,568,736
45-49 years	Male	White	2005-2009	5.9	2,710	46,037,878
45-49 years	Male	White	2010-2016	5.2	3,076	59,653,387
45-49 years	Male	Black	1975-1979	15.0	389	2,597,144
45-49 years	Male	Black	1980-1984	13.5	360	2,667,050
45-49 years	Male	Black	1985-1989	12.2	366	3,000,412
45-49 years	Male	Black	1990-1994	13.0	466	3,577,741
45-49 years	Male	Black	1995-1999	11.9	577	4,839,591
45-49 years	Male	Black	2000-2004	9.9	588	5,960,660
45-49 years	Male	Black	2005-2009	8.4	573	6,794,336
45-49 years	Male	Black	2010-2016	7.0	657	9,446,219
45-49 years	Male	Other	1975-1979	4.0	20	497,738
45-49 years	Male	Other	1980-1984	2.7	19	699,078
45-49 years	Male	Other	1985-1989	3.2	33	1,016,132
45-49 years	Male	Other	1990-1994	2.2	32	1,442,895
45-49 years	Male	Other	1995-1999	2.6	54	2,056,620
45-49 years	Male	Other	2000-2004	2.4	64	2,669,695
45-49 years	Male	Other	2005-2009	2.6	83	3,244,909
45-49 years	Male	Other	2010-2016	1.8	93	5,295,211
45-49 years	Female	All races	1975-1979	4.8	1,421	29,592,468

45-49 years	Female	All races	1980-1984	5.3	1,506	28,537,046
45-49 years	Female	All races	1985-1989	5.4	1,711	31,727,869
45-49 years	Female	All races	1990-1994	5.0	1,947	38,786,921
45-49 years	Female	All races	1995-1999	5.1	2,432	47,644,701
45-49 years	Female	All races	2000-2004	5.4	2,916	53,759,645
45-49 years	Female	All races	2005-2009	6.4	3,694	57,538,313
45-49 years	Female	All races	2010-2016	6.3	4,750	75,989,689
45-49 years	Female	White	1975-1979	4.6	1,184	25,891,298
45-49 years	Female	White	1980-1984	5.0	1,233	24,553,508
45-49 years	Female	White	1985-1989	5.0	1,353	27,063,820
45-49 years	Female	White	1990-1994	4.4	1,445	32,948,000
45-49 years	Female	White	1995-1999	4.5	1,790	39,640,760
45-49 years	Female	White	2000-2004	5.1	2,225	43,939,671
45-49 years	Female	White	2005-2009	6.4	2,946	46,276,471
45-49 years	Female	White	2010-2016	6.5	3,865	59,533,605
45-49 years	Female	Black	1975-1979	7.2	227	3,139,776
45-49 years	Female	Black	1980-1984	8.1	260	3,229,752
45-49 years	Female	Black	1985-1989	9.3	335	3,600,469
45-49 years	Female	Black	1990-1994	11.0	469	4,246,697
45-49 years	Female	Black	1995-1999	10.4	590	5,661,936
45-49 years	Female	Black	2000-2004	9.2	627	6,817,251
45-49 years	Female	Black	2005-2009	9.0	686	7,664,721
45-49 years	Female	Black	2010-2016	7.5	794	10,620,469
45-49 years	Female	Other	1975-1979	1.8	10	561,394
45-49 years	Female	Other	1980-1984	1.7	13	753,786
45-49 years	Female	Other	1985-1989	2.2	23	1,063,580
45-49 years	Female	Other	1990-1994	2.1	33	1,592,224
45-49 years	Female	Other	1995-1999	2.2	52	2,342,005
45-49 years	Female	Other	2000-2004	2.1	64	3,002,723
45-49 years	Female	Other	2005-2009	1.7	62	3,597,121
45-49 years	Female	Other	2010-2016	1.6	91	5,835,615
50-54 years	Male and female	All races	1975-1979	13.5	8,021	59,368,570
50-54 years	Male and female	All races	1980-1984	13.7	7,756	56,814,559
50-54 years	Male and female	All races	1985-1989	14.2	7,765	54,642,885
50-54 years	Male and female	All races	1990-1994	13.1	8,040	61,416,658
50-54 years	Male and female	All races	1995-1999	12.3	9,444	76,578,111
50-54 years	Male and female	All races	2000-2004	11.8	11,063	93,868,584
50-54 years	Male and female	All races	2005-2009	13.3	14,050	105,434,101
50-54 years	Male and female	All races	2010-2016	14.5	22,716	156,779,379
50-54 years	Male and female	White	1975-1979	13.2	7,003	52,952,699
50-54 years	Male and female	White	1980-1984	13.4	6,672	49,934,433
50-54 years	Male and female	White	1985-1989	13.7	6,476	47,339,556
50-54 years	Male and female	White	1990-1994	12.7	6,724	52,768,918

50-54 years	Male and female	White	1995-1999	12.1	7,906	65,391,137
50-54 years	Male and female	White	2000-2004	11.6	9,110	78,527,710
50-54 years	Male and female	White	2005-2009	13.5	11,695	86,643,532
50-54 years	Male and female	White	2010-2016	15.2	19,256	126,399,538
50-54 years	Male and female	Black	1975-1979	17.7	977	5,507,581
50-54 years	Male and female	Black	1980-1984	18.2	1,025	5,638,097
50-54 years	Male and female	Black	1985-1989	20.7	1,173	5,654,737
50-54 years	Male and female	Black	1990-1994	19.1	1,218	6,361,075
50-54 years	Male and female	Black	1995-1999	17.7	1,388	7,849,700
50-54 years	Male and female	Black	2000-2004	16.8	1,764	10,530,635
50-54 years	Male and female	Black	2005-2009	16.6	2,113	12,747,324
50-54 years	Male and female	Black	2010-2016	15.4	3,101	20,121,880
50-54 years	Male and female	Other	1975-1979	4.5	41	908,290
50-54 years	Male and female	Other	1980-1984	4.8	59	1,242,029
50-54 years	Male and female	Other	1985-1989	7.0	116	1,648,592
50-54 years	Male and female	Other	1990-1994	4.3	98	2,286,665
50-54 years	Male and female	Other	1995-1999	4.5	150	3,337,274
50-54 years	Male and female	Other	2000-2004	3.9	189	4,810,239
50-54 years	Male and female	Other	2005-2009	4.0	242	6,043,245
50-54 years	Male and female	Other	2010-2016	3.5	359	10,257,961
50-54 years	Male	All races	1975-1979	17.7	5,039	28,430,296
50-54 years	Male	All races	1980-1984	16.6	4,555	27,368,751
50-54 years	Male	All races	1985-1989	15.7	4,149	26,472,414
50-54 years	Male	All races	1990-1994	13.7	4,097	29,882,124
50-54 years	Male	All races	1995-1999	12.5	4,691	37,402,955
50-54 years	Male	All races	2000-2004	12.3	5,671	45,920,301
50-54 years	Male	All races	2005-2009	14.0	7,233	51,612,958
50-54 years	Male	All races	2010-2016	13.8	10,612	76,929,120
50-54 years	Male	White	1975-1979	17.1	4,353	25,526,261
50-54 years	Male	White	1980-1984	16.0	3,879	24,274,955
50-54 years	Male	White	1985-1989	14.7	3,397	23,140,735
50-54 years	Male	White	1990-1994	13.1	3,403	25,904,278
50-54 years	Male	White	1995-1999	12.1	3,916	32,239,526
50-54 years	Male	White	2000-2004	12.0	4,655	38,819,396
50-54 years	Male	White	2005-2009	14.1	6,039	42,859,191
50-54 years	Male	White	2010-2016	14.4	9,003	62,674,400
50-54 years	Male	Black	1975-1979	26.5	657	2,481,609
50-54 years	Male	Black	1980-1984	25.2	637	2,526,547
50-54 years	Male	Black	1985-1989	27.3	695	2,545,883
50-54 years	Male	Black	1990-1994	22.2	639	2,877,298
50-54 years	Male	Black	1995-1999	19.5	698	3,583,180
50-54 years	Male	Black	2000-2004	18.6	903	4,860,298
50-54 years	Male	Black	2005-2009	18.0	1,069	5,935,602

50-54 years	Male	Black	2010-2016	15.1	1,427	9,424,510
50-54 years	Male	Other	1975-1979	6.9	29	422,426
50-54 years	Male	Other	1980-1984	6.9	39	567,249
50-54 years	Male	Other	1985-1989	7.3	57	785,796
50-54 years	Male	Other	1990-1994	5.0	55	1,100,548
50-54 years	Male	Other	1995-1999	4.9	77	1,580,249
50-54 years	Male	Other	2000-2004	5.0	113	2,240,607
50-54 years	Male	Other	2005-2009	4.4	125	2,818,165
50-54 years	Male	Other	2010-2016	3.8	182	4,830,210
50-54 years	Female	All races	1975-1979	9.6	2,982	30,938,274
50-54 years	Female	All races	1980-1984	10.9	3,201	29,445,808
50-54 years	Female	All races	1985-1989	12.8	3,616	28,170,471
50-54 years	Female	All races	1990-1994	12.5	3,943	31,534,534
50-54 years	Female	All races	1995-1999	12.1	4,753	39,175,156
50-54 years	Female	All races	2000-2004	11.2	5,392	47,948,283
50-54 years	Female	All races	2005-2009	12.7	6,817	53,821,143
50-54 years	Female	All races	2010-2016	15.2	12,104	79,850,259
50-54 years	Female	White	1975-1979	9.7	2,650	27,426,438
50-54 years	Female	White	1980-1984	10.9	2,793	25,659,478
50-54 years	Female	White	1985-1989	12.7	3,079	24,198,821
50-54 years	Female	White	1990-1994	12.4	3,321	26,864,640
50-54 years	Female	White	1995-1999	12.0	3,990	33,151,611
50-54 years	Female	White	2000-2004	11.2	4,455	39,708,314
50-54 years	Female	White	2005-2009	12.9	5,656	43,784,341
50-54 years	Female	White	2010-2016	16.1	10,253	63,725,138
50-54 years	Female	Black	1975-1979	10.6	320	3,025,972
50-54 years	Female	Black	1980-1984	12.5	388	3,111,550
50-54 years	Female	Black	1985-1989	15.4	478	3,108,854
50-54 years	Female	Black	1990-1994	16.6	579	3,483,777
50-54 years	Female	Black	1995-1999	16.2	690	4,266,520
50-54 years	Female	Black	2000-2004	15.2	861	5,670,337
50-54 years	Female	Black	2005-2009	15.3	1,044	6,811,722
50-54 years	Female	Black	2010-2016	15.6	1,674	10,697,370
50-54 years	Female	Other	1975-1979	2.5	12	485,864
50-54 years	Female	Other	1980-1984	3.0	20	674,780
50-54 years	Female	Other	1985-1989	6.8	59	862,796
50-54 years	Female	Other	1990-1994	3.6	43	1,186,117
50-54 years	Female	Other	1995-1999	4.2	73	1,757,025
50-54 years	Female	Other	2000-2004	3.0	76	2,569,632
50-54 years	Female	Other	2005-2009	3.6	117	3,225,080
50-54 years	Female	Other	2010-2016	3.3	177	5,427,751
55-59 years	Male and female	All races	1975-1979	28.5	15,705	55,188,233
55-59 years	Male and female	All races	1980-1984	30.0	17,233	57,437,550

55-59 years	Male and female	All races	1985-1989	32.5	17,717	54,588,564
55-59 years	Male and female	All races	1990-1994	31.8	16,909	53,187,067
55-59 years	Male and female	All races	1995-1999	30.0	18,046	60,242,353
55-59 years	Male and female	All races	2000-2004	27.9	20,930	75,099,712
55-59 years	Male and female	All races	2005-2009	26.3	24,304	92,450,023
55-59 years	Male and female	All races	2010-2016	29.5	43,420	147,278,183
55-59 years	Male and female	White	1975-1979	28.7	14,221	49,549,841
55-59 years	Male and female	White	1980-1984	30.1	15,379	51,100,947
55-59 years	Male and female	White	1985-1989	32.5	15,598	47,931,591
55-59 years	Male and female	White	1990-1994	32.1	14,779	46,031,230
55-59 years	Male and female	White	1995-1999	30.4	15,691	51,680,339
55-59 years	Male and female	White	2000-2004	28.6	18,282	64,015,750
55-59 years	Male and female	White	2005-2009	27.4	21,113	77,154,798
55-59 years	Male and female	White	2010-2016	31.2	37,504	120,394,037
55-59 years	Male and female	Black	1975-1979	29.0	1,420	4,899,414
55-59 years	Male and female	Black	1980-1984	33.0	1,739	5,277,555
55-59 years	Male and female	Black	1985-1989	36.8	1,939	5,270,459
55-59 years	Male and female	Black	1990-1994	35.5	1,906	5,361,802
55-59 years	Male and female	Black	1995-1999	34.2	2,096	6,128,596
55-59 years	Male and female	Black	2000-2004	30.5	2,317	7,589,971
55-59 years	Male and female	Black	2005-2009	27.5	2,823	10,277,141
55-59 years	Male and female	Black	2010-2016	29.2	5,212	17,861,816
55-59 years	Male and female	Other	1975-1979	8.7	64	738,978
55-59 years	Male and female	Other	1980-1984	10.9	115	1,059,048
55-59 years	Male and female	Other	1985-1989	13.0	180	1,386,514
55-59 years	Male and female	Other	1990-1994	12.5	224	1,794,035
55-59 years	Male and female	Other	1995-1999	10.6	259	2,433,418
55-59 years	Male and female	Other	2000-2004	9.5	331	3,493,991
55-59 years	Male and female	Other	2005-2009	7.3	368	5,018,084
55-59 years	Male and female	Other	2010-2016	7.8	704	9,022,330
55-59 years	Male	All races	1975-1979	40.3	10,524	26,113,638
55-59 years	Male	All races	1980-1984	39.3	10,636	27,093,324
55-59 years	Male	All races	1985-1989	38.5	9,997	25,987,806
55-59 years	Male	All races	1990-1994	35.8	9,117	25,495,513
55-59 years	Male	All races	1995-1999	32.1	9,322	29,038,066
55-59 years	Male	All races	2000-2004	29.6	10,779	36,389,812
55-59 years	Male	All races	2005-2009	28.5	12,791	44,811,171
55-59 years	Male	All races	2010-2016	30.6	21,898	71,448,760
55-59 years	Male	White	1975-1979	40.2	9,451	23,539,009
55-59 years	Male	White	1980-1984	38.8	9,405	24,239,335
55-59 years	Male	White	1985-1989	37.9	8,734	23,017,314
55-59 years	Male	White	1990-1994	35.4	7,891	22,281,313
55-59 years	Male	White	1995-1999	32.0	8,037	25,141,241

55-59 years	Male	White	2000-2004	29.7	9,306	31,311,502
55-59 years	Male	White	2005-2009	29.2	11,054	37,804,279
55-59 years	Male	White	2010-2016	32.1	18,957	59,043,433
55-59 years	Male	Black	1975-1979	46.4	1,030	2,218,177
55-59 years	Male	Black	1980-1984	48.8	1,155	2,367,739
55-59 years	Male	Black	1985-1989	49.1	1,153	2,347,321
55-59 years	Male	Black	1990-1994	46.5	1,105	2,377,275
55-59 years	Male	Black	1995-1999	41.4	1,134	2,742,052
55-59 years	Male	Black	2000-2004	37.5	1,290	3,442,431
55-59 years	Male	Black	2005-2009	32.7	1,539	4,699,954
55-59 years	Male	Black	2010-2016	31.1	2,561	8,237,813
55-59 years	Male	Other	1975-1979	12.1	43	356,452
55-59 years	Male	Other	1980-1984	15.6	76	486,250
55-59 years	Male	Other	1985-1989	17.7	110	623,171
55-59 years	Male	Other	1990-1994	14.5	121	836,925
55-59 years	Male	Other	1995-1999	13.1	151	1,154,773
55-59 years	Male	Other	2000-2004	11.2	183	1,635,879
55-59 years	Male	Other	2005-2009	8.6	198	2,306,938
55-59 years	Male	Other	2010-2016	9.1	380	4,167,514
55-59 years	Female	All races	1975-1979	17.8	5,181	29,074,595
55-59 years	Female	All races	1980-1984	21.7	6,597	30,344,226
55-59 years	Female	All races	1985-1989	27.0	7,720	28,600,758
55-59 years	Female	All races	1990-1994	28.1	7,792	27,691,554
55-59 years	Female	All races	1995-1999	28.0	8,724	31,204,287
55-59 years	Female	All races	2000-2004	26.2	10,151	38,709,900
55-59 years	Female	All races	2005-2009	24.2	11,513	47,638,852
55-59 years	Female	All races	2010-2016	28.4	21,522	75,829,423
55-59 years	Female	White	1975-1979	18.3	4,770	26,010,832
55-59 years	Female	White	1980-1984	22.2	5,974	26,861,612
55-59 years	Female	White	1985-1989	27.6	6,864	24,914,277
55-59 years	Female	White	1990-1994	29.0	6,888	23,749,917
55-59 years	Female	White	1995-1999	28.8	7,654	26,539,098
55-59 years	Female	White	2000-2004	27.4	8,976	32,704,248
55-59 years	Female	White	2005-2009	25.6	10,059	39,350,519
55-59 years	Female	White	2010-2016	30.2	18,547	61,350,604
55-59 years	Female	Black	1975-1979	14.5	390	2,681,237
55-59 years	Female	Black	1980-1984	20.1	584	2,909,816
55-59 years	Female	Black	1985-1989	26.9	786	2,923,138
55-59 years	Female	Black	1990-1994	26.8	801	2,984,527
55-59 years	Female	Black	1995-1999	28.4	962	3,386,544
55-59 years	Female	Black	2000-2004	24.8	1,027	4,147,540
55-59 years	Female	Black	2005-2009	23.0	1,284	5,577,187
55-59 years	Female	Black	2010-2016	27.5	2,651	9,624,003

55-59 years	Female	Other	1975-1979	5.5	21	382,526
55-59 years	Female	Other	1980-1984	6.8	39	572,798
55-59 years	Female	Other	1985-1989	9.2	70	763,343
55-59 years	Female	Other	1990-1994	10.8	103	957,110
55-59 years	Female	Other	1995-1999	8.4	108	1,278,645
55-59 years	Female	Other	2000-2004	8.0	148	1,858,112
55-59 years	Female	Other	2005-2009	6.3	170	2,711,146
55-59 years	Female	Other	2010-2016	6.7	324	4,854,816
60-64 years	Male and female	All races	1975-1979	56.7	27,397	48,354,085
60-64 years	Male and female	All races	1980-1984	59.0	31,016	52,528,574
60-64 years	Male and female	All races	1985-1989	65.2	35,230	54,047,409
60-64 years	Male and female	All races	1990-1994	67.0	34,955	52,146,101
60-64 years	Male and female	All races	1995-1999	65.1	33,556	51,555,947
60-64 years	Male and female	All races	2000-2004	61.7	36,086	58,452,230
60-64 years	Male and female	All races	2005-2009	57.4	41,797	72,812,684
60-64 years	Male and female	All races	2010-2016	53.8	68,779	127,834,769
60-64 years	Male and female	White	1975-1979	58.4	25,423	43,512,307
60-64 years	Male and female	White	1980-1984	60.2	28,401	47,149,282
60-64 years	Male and female	White	1985-1989	66.5	31,932	48,040,311
60-64 years	Male and female	White	1990-1994	69.2	31,617	45,703,788
60-64 years	Male and female	White	1995-1999	67.7	30,122	44,488,083
60-64 years	Male and female	White	2000-2004	65.0	32,523	50,019,194
60-64 years	Male and female	White	2005-2009	60.9	37,752	61,975,542
60-64 years	Male and female	White	2010-2016	57.2	60,717	106,226,312
60-64 years	Male and female	Black	1975-1979	43.8	1,864	4,253,957
60-64 years	Male and female	Black	1980-1984	54.0	2,446	4,527,480
60-64 years	Male and female	Black	1985-1989	62.2	2,992	4,806,964
60-64 years	Male and female	Black	1990-1994	60.5	2,976	4,920,184
60-64 years	Male and female	Black	1995-1999	58.2	2,987	5,129,832
60-64 years	Male and female	Black	2000-2004	52.8	3,087	5,843,569
60-64 years	Male and female	Black	2005-2009	48.4	3,499	7,231,809
60-64 years	Male and female	Black	2010-2016	49.3	6,986	14,162,265
60-64 years	Male and female	Other	1975-1979	18.7	110	587,821
60-64 years	Male and female	Other	1980-1984	19.8	169	851,812
60-64 years	Male and female	Other	1985-1989	25.5	306	1,200,134
60-64 years	Male and female	Other	1990-1994	23.8	362	1,522,129
60-64 years	Male and female	Other	1995-1999	23.1	447	1,938,032
60-64 years	Male and female	Other	2000-2004	18.4	476	2,589,467
60-64 years	Male and female	Other	2005-2009	15.1	546	3,605,333
60-64 years	Male and female	Other	2010-2016	14.5	1,076	7,446,192
60-64 years	Male	All races	1975-1979	86.4	19,370	22,431,135
60-64 years	Male	All races	1980-1984	81.9	19,966	24,375,120
60-64 years	Male	All races	1985-1989	83.0	20,798	25,046,997

60-64 years	Male	All races	1990-1994	79.2	19,354	24,437,615
60-64 years	Male	All races	1995-1999	73.1	17,854	24,410,237
60-64 years	Male	All races	2000-2004	66.6	18,542	27,854,925
60-64 years	Male	All races	2005-2009	62.0	21,630	34,891,097
60-64 years	Male	All races	2010-2016	59.2	36,197	61,193,341
60-64 years	Male	White	1975-1979	88.4	17,904	20,246,193
60-64 years	Male	White	1980-1984	82.5	18,147	21,992,215
60-64 years	Male	White	1985-1989	83.4	18,707	22,428,464
60-64 years	Male	White	1990-1994	80.2	17,341	21,632,915
60-64 years	Male	White	1995-1999	74.5	15,839	21,270,383
60-64 years	Male	White	2000-2004	68.6	16,505	24,052,682
60-64 years	Male	White	2005-2009	64.4	19,315	29,997,821
60-64 years	Male	White	2010-2016	61.9	31,848	51,455,919
60-64 years	Male	Black	1975-1979	73.6	1,396	1,896,970
60-64 years	Male	Black	1980-1984	85.5	1,701	1,988,440
60-64 years	Male	Black	1985-1989	91.4	1,903	2,081,951
60-64 years	Male	Black	1990-1994	84.6	1,805	2,133,385
60-64 years	Male	Black	1995-1999	78.3	1,761	2,248,244
60-64 years	Male	Black	2000-2004	67.8	1,753	2,584,501
60-64 years	Male	Black	2005-2009	61.8	1,996	3,229,971
60-64 years	Male	Black	2010-2016	59.1	3,756	6,359,484
60-64 years	Male	Other	1975-1979	24.3	70	287,972
60-64 years	Male	Other	1980-1984	29.9	118	394,465
60-64 years	Male	Other	1985-1989	35.0	188	536,582
60-64 years	Male	Other	1990-1994	31.0	208	671,315
60-64 years	Male	Other	1995-1999	28.5	254	891,610
60-64 years	Male	Other	2000-2004	23.3	284	1,217,742
60-64 years	Male	Other	2005-2009	19.2	319	1,663,305
60-64 years	Male	Other	2010-2016	17.6	593	3,377,938
60-64 years	Female	All races	1975-1979	31.0	8,027	25,922,950
60-64 years	Female	All races	1980-1984	39.2	11,050	28,153,454
60-64 years	Female	All races	1985-1989	49.8	14,432	29,000,412
60-64 years	Female	All races	1990-1994	56.3	15,601	27,708,486
60-64 years	Female	All races	1995-1999	57.8	15,702	27,145,710
60-64 years	Female	All races	2000-2004	57.3	17,544	30,597,305
60-64 years	Female	All races	2005-2009	53.2	20,167	37,921,587
60-64 years	Female	All races	2010-2016	48.9	32,582	66,641,428
60-64 years	Female	White	1975-1979	32.3	7,519	23,266,114
60-64 years	Female	White	1980-1984	40.8	10,254	25,157,067
60-64 years	Female	White	1985-1989	51.6	13,225	25,611,847
60-64 years	Female	White	1990-1994	59.3	14,276	24,070,873
60-64 years	Female	White	1995-1999	61.5	14,283	23,217,700
60-64 years	Female	White	2000-2004	61.7	16,018	25,966,512

60-64 years	Female	White	2005-2009	57.7	18,437	31,977,721
60-64 years	Female	White	2010-2016	52.7	28,869	54,770,393
60-64 years	Female	Black	1975-1979	19.9	468	2,356,987
60-64 years	Female	Black	1980-1984	29.3	745	2,539,040
60-64 years	Female	Black	1985-1989	40.0	1,089	2,725,013
60-64 years	Female	Black	1990-1994	42.0	1,171	2,786,799
60-64 years	Female	Black	1995-1999	42.5	1,226	2,881,588
60-64 years	Female	Black	2000-2004	40.9	1,334	3,259,068
60-64 years	Female	Black	2005-2009	37.6	1,503	4,001,838
60-64 years	Female	Black	2010-2016	41.4	3,230	7,802,781
60-64 years	Female	Other	1975-1979	13.3	40	299,849
60-64 years	Female	Other	1980-1984	11.2	51	457,347
60-64 years	Female	Other	1985-1989	17.8	118	663,552
60-64 years	Female	Other	1990-1994	18.1	154	850,814
60-64 years	Female	Other	1995-1999	18.4	193	1,046,422
60-64 years	Female	Other	2000-2004	14.0	192	1,371,725
60-64 years	Female	Other	2005-2009	11.7	227	1,942,028
60-64 years	Female	Other	2010-2016	11.9	483	4,068,254
65-69 years	Male and female	All races	1975-1979	95.6	39,686	41,502,333
65-69 years	Male and female	All races	1980-1984	106.9	48,167	45,053,831
65-69 years	Male and female	All races	1985-1989	115.3	55,818	48,407,302
65-69 years	Male and female	All races	1990-1994	122.8	61,597	50,164,366
65-69 years	Male and female	All races	1995-1999	124.5	60,998	49,009,165
65-69 years	Male and female	All races	2000-2004	119.7	58,204	48,628,848
65-69 years	Male and female	All races	2005-2009	112.9	62,674	55,488,787
65-69 years	Male and female	All races	2010-2016	102.8	105,037	102,195,357
65-69 years	Male and female	White	1975-1979	100.2	37,389	37,312,733
65-69 years	Male and female	White	1980-1984	111.4	45,053	40,456,800
65-69 years	Male and female	White	1985-1989	118.8	51,507	43,350,310
65-69 years	Male and female	White	1990-1994	127.3	56,706	44,544,395
65-69 years	Male and female	White	1995-1999	130.5	55,960	42,896,700
65-69 years	Male and female	White	2000-2004	127.3	53,295	41,866,447
65-69 years	Male and female	White	2005-2009	120.9	57,312	47,418,200
65-69 years	Male and female	White	2010-2016	110.1	95,276	86,525,931
65-69 years	Male and female	Black	1975-1979	58.1	2,155	3,707,977
65-69 years	Male and female	Black	1980-1984	72.3	2,845	3,933,236
65-69 years	Male and female	Black	1985-1989	94.8	3,904	4,119,431
65-69 years	Male and female	Black	1990-1994	98.4	4,299	4,368,702
65-69 years	Male and female	Black	1995-1999	95.2	4,318	4,537,812
65-69 years	Male and female	Black	2000-2004	87.8	4,181	4,759,258
65-69 years	Male and female	Black	2005-2009	84.2	4,568	5,426,555
65-69 years	Male and female	Black	2010-2016	81.7	8,275	10,125,763
65-69 years	Male and female	Other	1975-1979	29.5	142	481,623

65-69 years	Male and female	Other	1980-1984	40.5	269	663,795
65-69 years	Male and female	Other	1985-1989	43.4	407	937,561
65-69 years	Male and female	Other	1990-1994	47.3	592	1,251,269
65-69 years	Male and female	Other	1995-1999	45.7	720	1,574,653
65-69 years	Male and female	Other	2000-2004	36.3	728	2,003,143
65-69 years	Male and female	Other	2005-2009	30.0	794	2,644,032
65-69 years	Male and female	Other	2010-2016	26.8	1,486	5,543,663
65-69 years	Male	All races	1975-1979	160.2	29,488	18,411,774
65-69 years	Male	All races	1980-1984	158.7	31,841	20,058,942
65-69 years	Male	All races	1985-1989	156.1	33,875	21,702,282
65-69 years	Male	All races	1990-1994	153.3	34,569	22,554,707
65-69 years	Male	All races	1995-1999	146.5	32,897	22,456,458
65-69 years	Male	All races	2000-2004	132.3	29,877	22,580,860
65-69 years	Male	All races	2005-2009	122.9	31,993	26,029,494
65-69 years	Male	All races	2010-2016	112.0	54,044	48,258,343
65-69 years	Male	White	1975-1979	166.9	27,647	16,563,400
65-69 years	Male	White	1980-1984	163.4	29,541	18,082,433
65-69 years	Male	White	1985-1989	158.3	30,943	19,541,490
65-69 years	Male	White	1990-1994	156.4	31,535	20,164,002
65-69 years	Male	White	1995-1999	150.7	29,894	19,842,053
65-69 years	Male	White	2000-2004	137.8	27,069	19,637,961
65-69 years	Male	White	2005-2009	129.0	28,967	22,461,533
65-69 years	Male	White	2010-2016	117.4	48,530	41,330,272
65-69 years	Male	Black	1975-1979	107.9	1,731	1,604,153
65-69 years	Male	Black	1980-1984	126.4	2,112	1,670,254
65-69 years	Male	Black	1985-1989	153.5	2,668	1,737,966
65-69 years	Male	Black	1990-1994	143.9	2,655	1,844,632
65-69 years	Male	Black	1995-1999	133.0	2,569	1,932,039
65-69 years	Male	Black	2000-2004	117.9	2,403	2,037,720
65-69 years	Male	Black	2005-2009	110.0	2,581	2,345,856
65-69 years	Male	Black	2010-2016	105.6	4,663	4,417,362
65-69 years	Male	Other	1975-1979	45.0	110	244,221
65-69 years	Male	Other	1980-1984	61.4	188	306,255
65-69 years	Male	Other	1985-1989	62.4	264	422,826
65-69 years	Male	Other	1990-1994	69.4	379	546,073
65-69 years	Male	Other	1995-1999	63.6	434	682,366
65-69 years	Male	Other	2000-2004	44.7	405	905,179
65-69 years	Male	Other	2005-2009	36.4	445	1,222,105
65-69 years	Male	Other	2010-2016	33.9	851	2,510,709
65-69 years	Female	All races	1975-1979	44.2	10,198	23,090,559
65-69 years	Female	All races	1980-1984	65.3	16,326	24,994,889
65-69 years	Female	All races	1985-1989	82.2	21,943	26,705,020
65-69 years	Female	All races	1990-1994	97.9	27,028	27,609,659

65-69 years	Female	All races	1995-1999	105.8	28,101	26,552,707
65-69 years	Female	All races	2000-2004	108.7	28,327	26,047,988
65-69 years	Female	All races	2005-2009	104.1	30,681	29,459,293
65-69 years	Female	All races	2010-2016	94.5	50,993	53,937,014
65-69 years	Female	White	1975-1979	47.0	9,742	20,749,333
65-69 years	Female	White	1980-1984	69.3	15,512	22,374,367
65-69 years	Female	White	1985-1989	86.4	20,564	23,808,820
65-69 years	Female	White	1990-1994	103.2	25,171	24,380,393
65-69 years	Female	White	1995-1999	113.1	26,066	23,054,647
65-69 years	Female	White	2000-2004	118.0	26,226	22,228,486
65-69 years	Female	White	2005-2009	113.6	28,345	24,956,667
65-69 years	Female	White	2010-2016	103.4	46,746	45,195,659
65-69 years	Female	Black	1975-1979	20.2	424	2,103,824
65-69 years	Female	Black	1980-1984	32.4	733	2,262,982
65-69 years	Female	Black	1985-1989	51.9	1,236	2,381,465
65-69 years	Female	Black	1990-1994	65.1	1,644	2,524,070
65-69 years	Female	Black	1995-1999	67.1	1,749	2,605,773
65-69 years	Female	Black	2000-2004	65.3	1,778	2,721,538
65-69 years	Female	Black	2005-2009	64.5	1,987	3,080,699
65-69 years	Female	Black	2010-2016	63.3	3,612	5,708,401
65-69 years	Female	Other	1975-1979	13.5	32	237,402
65-69 years	Female	Other	1980-1984	22.7	81	357,540
65-69 years	Female	Other	1985-1989	27.8	143	514,735
65-69 years	Female	Other	1990-1994	30.2	213	705,196
65-69 years	Female	Other	1995-1999	32.1	286	892,287
65-69 years	Female	Other	2000-2004	29.4	323	1,097,964
65-69 years	Female	Other	2005-2009	24.5	349	1,421,927
65-69 years	Female	Other	2010-2016	20.9	635	3,032,954
70-74 years	Male and female	All races	1975-1979	142.0	45,065	31,735,649
70-74 years	Male and female	All races	1980-1984	170.8	60,945	35,677,877
70-74 years	Male and female	All races	1985-1989	195.2	75,154	38,508,900
70-74 years	Male and female	All races	1990-1994	201.7	85,109	42,201,953
70-74 years	Male and female	All races	1995-1999	211.0	93,606	44,354,441
70-74 years	Male and female	All races	2000-2004	210.1	91,801	43,696,251
70-74 years	Male and female	All races	2005-2009	198.5	87,425	44,033,442
70-74 years	Male and female	All races	2010-2016	189.4	140,013	73,937,771
70-74 years	Male and female	White	1975-1979	149.0	42,858	28,773,243
70-74 years	Male and female	White	1980-1984	178.6	57,559	32,233,076
70-74 years	Male and female	White	1985-1989	202.8	70,414	34,718,807
70-74 years	Male and female	White	1990-1994	209.4	79,431	37,938,430
70-74 years	Male and female	White	1995-1999	220.0	87,029	39,559,939
70-74 years	Male and female	White	2000-2004	222.4	85,235	38,328,725
70-74 years	Male and female	White	2005-2009	213.4	80,935	37,934,868

70-74 years	Male and female	White	2010-2016	203.7	128,623	63,138,727
70-74 years	Male and female	Black	1975-1979	76.3	1,994	2,612,899
70-74 years	Male and female	Black	1980-1984	103.4	3,062	2,961,228
70-74 years	Male and female	Black	1985-1989	134.8	4,219	3,130,764
70-74 years	Male and female	Black	1990-1994	145.4	4,883	3,358,469
70-74 years	Male and female	Black	1995-1999	155.1	5,544	3,573,540
70-74 years	Male and female	Black	2000-2004	142.5	5,438	3,817,235
70-74 years	Male and female	Black	2005-2009	129.1	5,354	4,146,251
70-74 years	Male and female	Black	2010-2016	134.8	9,377	6,958,600
70-74 years	Male and female	Other	1975-1979	60.9	213	349,507
70-74 years	Male and female	Other	1980-1984	67.0	324	483,573
70-74 years	Male and female	Other	1985-1989	79.0	521	659,329
70-74 years	Male and female	Other	1990-1994	87.8	795	905,054
70-74 years	Male and female	Other	1995-1999	84.6	1,033	1,220,962
70-74 years	Male and female	Other	2000-2004	72.8	1,128	1,550,291
70-74 years	Male and female	Other	2005-2009	58.2	1,136	1,952,323
70-74 years	Male and female	Other	2010-2016	52.4	2,013	3,840,444
70-74 years	Male	All races	1975-1979	258.2	34,407	13,328,186
70-74 years	Male	All races	1980-1984	277.6	41,569	14,973,279
70-74 years	Male	All races	1985-1989	283.6	46,112	16,261,107
70-74 years	Male	All races	1990-1994	263.9	47,939	18,163,393
70-74 years	Male	All races	1995-1999	260.2	50,276	19,321,502
70-74 years	Male	All races	2000-2004	243.6	47,385	19,450,564
70-74 years	Male	All races	2005-2009	220.9	44,108	19,968,325
70-74 years	Male	All races	2010-2016	207.1	70,421	34,002,448
70-74 years	Male	White	1975-1979	270.8	32,618	12,046,519
70-74 years	Male	White	1980-1984	288.1	38,988	13,532,804
70-74 years	Male	White	1985-1989	290.3	42,732	14,720,381
70-74 years	Male	White	1990-1994	269.2	44,223	16,427,388
70-74 years	Male	White	1995-1999	266.6	46,325	17,375,249
70-74 years	Male	White	2000-2004	252.3	43,518	17,246,601
70-74 years	Male	White	2005-2009	232.8	40,480	17,390,409
70-74 years	Male	White	2010-2016	218.2	64,046	29,357,583
70-74 years	Male	Black	1975-1979	147.6	1,628	1,103,197
70-74 years	Male	Black	1980-1984	193.0	2,329	1,206,785
70-74 years	Male	Black	1985-1989	242.7	3,020	1,244,125
70-74 years	Male	Black	1990-1994	238.3	3,180	1,334,504
70-74 years	Male	Black	1995-1999	232.3	3,295	1,418,422
70-74 years	Male	Black	2000-2004	206.9	3,188	1,541,142
70-74 years	Male	Black	2005-2009	173.8	2,975	1,711,886
70-74 years	Male	Black	2010-2016	180.0	5,238	2,909,379
70-74 years	Male	Other	1975-1979	90.2	161	178,470
70-74 years	Male	Other	1980-1984	107.8	252	233,690

70-74 years	Male	Other	1985-1989	121.4	360	296,601
70-74 years	Male	Other	1990-1994	133.5	536	401,501
70-74 years	Male	Other	1995-1999	124.3	656	527,831
70-74 years	Male	Other	2000-2004	102.4	679	662,821
70-74 years	Male	Other	2005-2009	75.4	653	866,030
70-74 years	Male	Other	2010-2016	65.5	1,137	1,735,486
70-74 years	Female	All races	1975-1979	57.9	10,658	18,407,463
70-74 years	Female	All races	1980-1984	93.6	19,376	20,704,598
70-74 years	Female	All races	1985-1989	130.5	29,042	22,247,793
70-74 years	Female	All races	1990-1994	154.6	37,170	24,038,560
70-74 years	Female	All races	1995-1999	173.1	43,330	25,032,939
70-74 years	Female	All races	2000-2004	183.2	44,416	24,245,687
70-74 years	Female	All races	2005-2009	180.0	43,317	24,065,117
70-74 years	Female	All races	2010-2016	174.3	69,592	39,935,323
70-74 years	Female	White	1975-1979	61.2	10,240	16,726,724
70-74 years	Female	White	1980-1984	99.3	18,571	18,700,272
70-74 years	Female	White	1985-1989	138.4	27,682	19,998,426
70-74 years	Female	White	1990-1994	163.7	35,208	21,511,042
70-74 years	Female	White	1995-1999	183.5	40,704	22,184,690
70-74 years	Female	White	2000-2004	197.9	41,717	21,082,124
70-74 years	Female	White	2005-2009	196.9	40,455	20,544,459
70-74 years	Female	White	2010-2016	191.2	64,577	33,781,144
70-74 years	Female	Black	1975-1979	24.2	366	1,509,702
70-74 years	Female	Black	1980-1984	41.8	733	1,754,443
70-74 years	Female	Black	1985-1989	63.6	1,199	1,886,639
70-74 years	Female	Black	1990-1994	84.1	1,703	2,023,965
70-74 years	Female	Black	1995-1999	104.4	2,249	2,155,118
70-74 years	Female	Black	2000-2004	98.9	2,250	2,276,093
70-74 years	Female	Black	2005-2009	97.7	2,379	2,434,365
70-74 years	Female	Black	2010-2016	102.2	4,139	4,049,221
70-74 years	Female	Other	1975-1979	30.4	52	171,037
70-74 years	Female	Other	1980-1984	28.8	72	249,883
70-74 years	Female	Other	1985-1989	44.4	161	362,728
70-74 years	Female	Other	1990-1994	51.4	259	503,553
70-74 years	Female	Other	1995-1999	54.4	377	693,131
70-74 years	Female	Other	2000-2004	50.6	449	887,470
70-74 years	Female	Other	2005-2009	44.5	483	1,086,293
70-74 years	Female	Other	2010-2016	41.6	876	2,104,958
75-79 years	Male and female	All races	1975-1979	184.7	40,930	22,159,084
75-79 years	Male and female	All races	1980-1984	228.7	58,303	25,497,161
75-79 years	Male and female	All races	1985-1989	275.2	79,469	28,874,873
75-79 years	Male and female	All races	1990-1994	300.0	95,716	31,908,470
75-79 years	Male and female	All races	1995-1999	315.5	111,310	35,281,124

75-79 years	Male and female	All races	2000-2004	324.8	121,237	37,323,734
75-79 years	Male and female	All races	2005-2009	316.5	116,939	36,951,813
75-79 years	Male and female	All races	2010-2016	299.3	162,548	54,301,356
75-79 years	Male and female	White	1975-1979	193.3	39,002	20,173,020
75-79 years	Male and female	White	1980-1984	240.4	55,572	23,114,106
75-79 years	Male and female	White	1985-1989	287.5	75,095	26,121,954
75-79 years	Male and female	White	1990-1994	313.0	90,376	28,870,131
75-79 years	Male and female	White	1995-1999	328.7	104,440	31,773,008
75-79 years	Male and female	White	2000-2004	341.4	113,620	33,281,364
75-79 years	Male and female	White	2005-2009	336.2	109,214	32,480,098
75-79 years	Male and female	White	2010-2016	322.8	150,787	46,708,645
75-79 years	Male and female	Black	1975-1979	97.8	1,717	1,756,175
75-79 years	Male and female	Black	1980-1984	116.8	2,409	2,063,040
75-79 years	Male and female	Black	1985-1989	162.3	3,755	2,313,031
75-79 years	Male and female	Black	1990-1994	184.4	4,541	2,462,256
75-79 years	Male and female	Black	1995-1999	209.2	5,651	2,701,117
75-79 years	Male and female	Black	2000-2004	208.2	6,093	2,926,882
75-79 years	Male and female	Black	2005-2009	199.7	6,125	3,067,508
75-79 years	Male and female	Black	2010-2016	188.2	9,249	4,913,180
75-79 years	Male and female	Other	1975-1979	91.8	211	229,889
75-79 years	Male and female	Other	1980-1984	100.6	322	320,015
75-79 years	Male and female	Other	1985-1989	140.7	619	439,888
75-79 years	Male and female	Other	1990-1994	138.7	799	576,083
75-79 years	Male and female	Other	1995-1999	151.1	1,219	806,999
75-79 years	Male and female	Other	2000-2004	136.6	1,524	1,115,488
75-79 years	Male and female	Other	2005-2009	113.9	1,600	1,404,207
75-79 years	Male and female	Other	2010-2016	93.7	2,512	2,679,531
75-79 years	Male	All races	1975-1979	365.6	31,318	8,565,875
75-79 years	Male	All races	1980-1984	414.7	40,764	9,829,637
75-79 years	Male	All races	1985-1989	440.0	49,341	11,214,387
75-79 years	Male	All races	1990-1994	423.6	53,644	12,663,765
75-79 years	Male	All races	1995-1999	408.4	58,691	14,369,445
75-79 years	Male	All races	2000-2004	394.6	61,198	15,507,845
75-79 years	Male	All races	2005-2009	366.7	58,107	15,843,920
75-79 years	Male	All races	2010-2016	332.7	79,675	23,949,218
75-79 years	Male	White	1975-1979	384.5	29,792	7,747,318
75-79 years	Male	White	1980-1984	436.4	38,716	8,871,525
75-79 years	Male	White	1985-1989	455.1	46,143	10,139,941
75-79 years	Male	White	1990-1994	435.5	50,098	11,504,642
75-79 years	Male	White	1995-1999	419.0	54,511	13,009,087
75-79 years	Male	White	2000-2004	406.4	56,669	13,942,436
75-79 years	Male	White	2005-2009	381.1	53,719	14,094,442
75-79 years	Male	White	2010-2016	351.4	73,258	20,846,660

75-79 years	Male	Black	1975-1979	194.6	1,379	708,805
75-79 years	Male	Black	1980-1984	225.6	1,816	805,132
75-79 years	Male	Black	1985-1989	312.6	2,728	872,574
75-79 years	Male	Black	1990-1994	333.6	3,024	906,607
75-79 years	Male	Black	1995-1999	336.7	3,415	1,014,349
75-79 years	Male	Black	2000-2004	320.7	3,520	1,097,749
75-79 years	Male	Black	2005-2009	295.5	3,444	1,165,666
75-79 years	Male	Black	2010-2016	257.6	4,977	1,931,860
75-79 years	Male	Other	1975-1979	133.9	147	109,752
75-79 years	Male	Other	1980-1984	151.7	232	152,980
75-79 years	Male	Other	1985-1989	232.8	470	201,872
75-79 years	Male	Other	1990-1994	206.7	522	252,516
75-79 years	Male	Other	1995-1999	221.1	765	346,009
75-79 years	Male	Other	2000-2004	215.8	1,009	467,660
75-79 years	Male	Other	2005-2009	161.7	944	583,812
75-79 years	Male	Other	2010-2016	123.0	1,440	1,170,698
75-79 years	Female	All races	1975-1979	70.7	9,612	13,593,209
75-79 years	Female	All races	1980-1984	111.9	17,539	15,667,524
75-79 years	Female	All races	1985-1989	170.6	30,128	17,660,486
75-79 years	Female	All races	1990-1994	218.6	42,072	19,244,705
75-79 years	Female	All races	1995-1999	251.6	52,619	20,911,679
75-79 years	Female	All races	2000-2004	275.2	60,039	21,815,889
75-79 years	Female	All races	2005-2009	278.7	58,832	21,107,893
75-79 years	Female	All races	2010-2016	273.0	82,873	30,352,138
75-79 years	Female	White	1975-1979	74.1	9,210	12,425,702
75-79 years	Female	White	1980-1984	118.3	16,856	14,242,581
75-79 years	Female	White	1985-1989	181.2	28,952	15,982,013
75-79 years	Female	White	1990-1994	231.9	40,278	17,365,489
75-79 years	Female	White	1995-1999	266.1	49,929	18,763,921
75-79 years	Female	White	2000-2004	294.5	56,951	19,338,928
75-79 years	Female	White	2005-2009	301.8	55,495	18,385,656
75-79 years	Female	White	2010-2016	299.8	77,529	25,861,985
75-79 years	Female	Black	1975-1979	32.3	338	1,047,370
75-79 years	Female	Black	1980-1984	47.1	593	1,257,908
75-79 years	Female	Black	1985-1989	71.3	1,027	1,440,457
75-79 years	Female	Black	1990-1994	97.5	1,517	1,555,649
75-79 years	Female	Black	1995-1999	132.6	2,236	1,686,768
75-79 years	Female	Black	2000-2004	140.7	2,573	1,829,133
75-79 years	Female	Black	2005-2009	141.0	2,681	1,901,842
75-79 years	Female	Black	2010-2016	143.3	4,272	2,981,320
75-79 years	Female	Other	1975-1979	53.3	64	120,137
75-79 years	Female	Other	1980-1984	53.9	90	167,035
75-79 years	Female	Other	1985-1989	62.6	149	238,016

75-79 years	Female	Other	1990-1994	85.6	277	323,567
75-79 years	Female	Other	1995-1999	98.5	454	460,990
75-79 years	Female	Other	2000-2004	79.5	515	647,828
75-79 years	Female	Other	2005-2009	80.0	656	820,395
75-79 years	Female	Other	2010-2016	71.0	1,072	1,508,833
80-84 years	Male and female	All races	1975-1979	208.1	29,296	14,077,436
80-84 years	Male and female	All races	1980-1984	271.2	42,201	15,558,897
80-84 years	Male and female	All races	1985-1989	341.1	61,350	17,984,192
80-84 years	Male and female	All races	1990-1994	391.2	81,376	20,802,725
80-84 years	Male and female	All races	1995-1999	439.2	103,296	23,518,541
80-84 years	Male and female	All races	2000-2004	456.8	120,342	26,344,248
80-84 years	Male and female	All races	2005-2009	456.1	129,562	28,404,456
80-84 years	Male and female	All races	2010-2016	443.9	179,811	40,507,396
80-84 years	Male and female	White	1975-1979	216.2	28,132	13,013,528
80-84 years	Male and female	White	1980-1984	281.3	40,224	14,297,641
80-84 years	Male and female	White	1985-1989	354.2	58,224	16,436,226
80-84 years	Male and female	White	1990-1994	406.3	77,026	18,955,931
80-84 years	Male and female	White	1995-1999	457.2	97,766	21,383,995
80-84 years	Male and female	White	2000-2004	478.0	113,732	23,792,274
80-84 years	Male and female	White	2005-2009	479.5	121,802	25,401,652
80-84 years	Male and female	White	2010-2016	473.2	167,801	35,462,818
80-84 years	Male and female	Black	1975-1979	108.5	1,015	935,449
80-84 years	Male and female	Black	1980-1984	155.3	1,696	1,091,914
80-84 years	Male and female	Black	1985-1989	203.6	2,679	1,315,635
80-84 years	Male and female	Black	1990-1994	235.4	3,596	1,527,757
80-84 years	Male and female	Black	1995-1999	265.6	4,460	1,678,960
80-84 years	Male and female	Black	2000-2004	276.6	5,201	1,880,512
80-84 years	Male and female	Black	2005-2009	278.2	5,812	2,088,771
80-84 years	Male and female	Black	2010-2016	269.7	8,846	3,280,188
80-84 years	Male and female	Other	1975-1979	116.0	149	128,459
80-84 years	Male and female	Other	1980-1984	165.9	281	169,342
80-84 years	Male and female	Other	1985-1989	192.4	447	232,331
80-84 years	Male and female	Other	1990-1994	236.3	754	319,037
80-84 years	Male and female	Other	1995-1999	234.9	1,070	455,586
80-84 years	Male and female	Other	2000-2004	209.8	1,409	671,462
80-84 years	Male and female	Other	2005-2009	213.1	1,948	914,033
80-84 years	Male and female	Other	2010-2016	179.3	3,164	1,764,390
80-84 years	Male	All races	1975-1979	447.3	22,238	4,971,599
80-84 years	Male	All races	1980-1984	535.7	28,824	5,380,912
80-84 years	Male	All races	1985-1989	609.5	37,788	6,199,757
80-84 years	Male	All races	1990-1994	620.0	45,126	7,278,895
80-84 years	Male	All races	1995-1999	615.4	52,574	8,542,529
80-84 years	Male	All races	2000-2004	583.2	57,981	9,941,137

80-84 years	Male	All races	2005-2009	558.1	61,860	11,084,255
80-84 years	Male	All races	2010-2016	510.4	84,789	16,612,455
80-84 years	Male	White	1975-1979	467.7	21,330	4,560,585
80-84 years	Male	White	1980-1984	557.2	27,358	4,909,578
80-84 years	Male	White	1985-1989	632.1	35,624	5,635,751
80-84 years	Male	White	1990-1994	638.0	42,267	6,625,047
80-84 years	Male	White	1995-1999	630.5	49,191	7,801,394
80-84 years	Male	White	2000-2004	599.9	54,181	9,031,157
80-84 years	Male	White	2005-2009	575.9	57,620	10,004,406
80-84 years	Male	White	2010-2016	533.4	78,583	14,731,555
80-84 years	Male	Black	1975-1979	224.8	800	355,947
80-84 years	Male	Black	1980-1984	314.4	1,253	398,591
80-84 years	Male	Black	1985-1989	400.8	1,842	459,562
80-84 years	Male	Black	1990-1994	460.5	2,359	512,246
80-84 years	Male	Black	1995-1999	491.8	2,707	550,425
80-84 years	Male	Black	2000-2004	459.7	2,927	636,666
80-84 years	Male	Black	2005-2009	433.7	3,105	715,943
80-84 years	Male	Black	2010-2016	373.4	4,354	1,166,195
80-84 years	Male	Other	1975-1979	196.1	108	55,067
80-84 years	Male	Other	1980-1984	292.8	213	72,743
80-84 years	Male	Other	1985-1989	308.3	322	104,444
80-84 years	Male	Other	1990-1994	353.1	500	141,602
80-84 years	Male	Other	1995-1999	354.5	676	190,710
80-84 years	Male	Other	2000-2004	319.4	873	273,314
80-84 years	Male	Other	2005-2009	311.9	1,135	363,906
80-84 years	Male	Other	2010-2016	259.1	1,852	714,705
80-84 years	Female	All races	1975-1979	77.5	7,058	9,105,837
80-84 years	Female	All races	1980-1984	131.4	13,377	10,177,985
80-84 years	Female	All races	1985-1989	199.9	23,562	11,784,435
80-84 years	Female	All races	1990-1994	268.0	36,250	13,523,830
80-84 years	Female	All races	1995-1999	338.7	50,722	14,976,012
80-84 years	Female	All races	2000-2004	380.2	62,361	16,403,111
80-84 years	Female	All races	2005-2009	390.9	67,702	17,320,201
80-84 years	Female	All races	2010-2016	397.7	95,022	23,894,941
80-84 years	Female	White	1975-1979	80.5	6,802	8,452,943
80-84 years	Female	White	1980-1984	137.0	12,866	9,388,063
80-84 years	Female	White	1985-1989	209.3	22,600	10,800,475
80-84 years	Female	White	1990-1994	281.9	34,759	12,330,884
80-84 years	Female	White	1995-1999	357.6	48,575	13,582,601
80-84 years	Female	White	2000-2004	403.4	59,551	14,761,117
80-84 years	Female	White	2005-2009	416.8	64,182	15,397,246
80-84 years	Female	White	2010-2016	430.4	89,218	20,731,263
80-84 years	Female	Black	1975-1979	37.1	215	579,502

80-84 years	Female	Black	1980-1984	63.9	443	693,323
80-84 years	Female	Black	1985-1989	97.8	837	856,073
80-84 years	Female	Black	1990-1994	121.8	1,237	1,015,511
80-84 years	Female	Black	1995-1999	155.3	1,753	1,128,535
80-84 years	Female	Black	2000-2004	182.8	2,274	1,243,846
80-84 years	Female	Black	2005-2009	197.2	2,707	1,372,828
80-84 years	Female	Black	2010-2016	212.5	4,492	2,113,993
80-84 years	Female	Other	1975-1979	55.9	41	73,392
80-84 years	Female	Other	1980-1984	70.4	68	96,599
80-84 years	Female	Other	1985-1989	97.7	125	127,887
80-84 years	Female	Other	1990-1994	143.2	254	177,435
80-84 years	Female	Other	1995-1999	148.7	394	264,876
80-84 years	Female	Other	2000-2004	134.6	536	398,148
80-84 years	Female	Other	2005-2009	147.8	813	550,127
80-84 years	Female	Other	2010-2016	125.0	1,312	1,049,685
85+ years	Male and female	All races	1975-1979	215.6	21,396	9,922,337
85+ years	Male and female	All races	1980-1984	295.9	36,011	12,170,858
85+ years	Male and female	All races	1985-1989	387.1	54,522	14,086,125
85+ years	Male and female	All races	1990-1994	464.8	77,024	16,569,997
85+ years	Male and female	All races	1995-1999	568.1	111,157	19,567,643
85+ years	Male and female	All races	2000-2004	657.4	144,344	21,955,833
85+ years	Male and female	All races	2005-2009	679.0	170,844	25,161,914
85+ years	Male and female	All races	2010-2016	690.2	289,559	41,952,277
85+ years	Male and female	White	1975-1979	223.7	20,413	9,126,999
85+ years	Male and female	White	1980-1984	308.1	34,434	11,174,675
85+ years	Male and female	White	1985-1989	402.4	51,854	12,885,857
85+ years	Male and female	White	1990-1994	483.0	72,987	15,111,624
85+ years	Male and female	White	1995-1999	590.4	104,890	17,764,872
85+ years	Male and female	White	2000-2004	684.8	136,251	19,896,570
85+ years	Male and female	White	2005-2009	708.6	160,876	22,703,838
85+ years	Male and female	White	2010-2016	728.4	271,152	37,225,527
85+ years	Male and female	Black	1975-1979	113.3	796	702,724
85+ years	Male and female	Black	1980-1984	147.4	1,294	878,006
85+ years	Male and female	Black	1985-1989	210.5	2,183	1,036,930
85+ years	Male and female	Black	1990-1994	265.8	3,287	1,236,843
85+ years	Male and female	Black	1995-1999	336.0	4,974	1,480,243
85+ years	Male and female	Black	2000-2004	383.5	6,053	1,578,531
85+ years	Male and female	Black	2005-2009	406.8	7,171	1,762,654
85+ years	Male and female	Black	2010-2016	399.9	12,525	3,132,407
85+ years	Male and female	Other	1975-1979	201.9	187	92,614
85+ years	Male and female	Other	1980-1984	239.5	283	118,177
85+ years	Male and female	Other	1985-1989	296.9	485	163,338
85+ years	Male and female	Other	1990-1994	338.6	750	221,530

85+ years	Male and female	Other	1995-1999	400.9	1,293	322,528
85+ years	Male and female	Other	2000-2004	424.4	2,040	480,732
85+ years	Male and female	Other	2005-2009	402.2	2,797	695,422
85+ years	Male and female	Other	2010-2016	368.9	5,882	1,594,343
85+ years	Male	All races	1975-1979	472.8	14,720	3,113,247
85+ years	Male	All races	1980-1984	629.7	22,574	3,584,840
85+ years	Male	All races	1985-1989	768.5	30,588	3,979,982
85+ years	Male	All races	1990-1994	842.6	38,658	4,587,826
85+ years	Male	All races	1995-1999	919.2	50,773	5,523,831
85+ years	Male	All races	2000-2004	937.1	60,967	6,505,837
85+ years	Male	All races	2005-2009	883.9	70,304	7,953,631
85+ years	Male	All races	2010-2016	838.3	119,033	14,199,029
85+ years	Male	White	1975-1979	493.9	13,995	2,833,405
85+ years	Male	White	1980-1984	661.7	21,520	3,252,319
85+ years	Male	White	1985-1989	803.7	28,886	3,594,312
85+ years	Male	White	1990-1994	875.8	36,292	4,143,993
85+ years	Male	White	1995-1999	950.2	47,422	4,990,737
85+ years	Male	White	2000-2004	962.9	56,856	5,904,774
85+ years	Male	White	2005-2009	908.7	65,566	7,215,155
85+ years	Male	White	2010-2016	869.0	110,407	12,704,745
85+ years	Male	Black	1975-1979	239.2	575	240,374
85+ years	Male	Black	1980-1984	299.5	861	287,437
85+ years	Male	Black	1985-1989	432.6	1,386	320,352
85+ years	Male	Black	1990-1994	534.0	1,899	355,648
85+ years	Male	Black	1995-1999	633.5	2,583	407,764
85+ years	Male	Black	2000-2004	697.2	2,942	421,995
85+ years	Male	Black	2005-2009	662.6	3,203	483,363
85+ years	Male	Black	2010-2016	598.3	5,454	911,560
85+ years	Male	Other	1975-1979	380.1	150	39,468
85+ years	Male	Other	1980-1984	428.1	193	45,084
85+ years	Male	Other	1985-1989	483.8	316	65,318
85+ years	Male	Other	1990-1994	529.6	467	88,185
85+ years	Male	Other	1995-1999	612.8	768	125,330
85+ years	Male	Other	2000-2004	652.8	1,169	179,068
85+ years	Male	Other	2005-2009	601.7	1,535	255,113
85+ years	Male	Other	2010-2016	544.3	3,172	582,724
85+ years	Female	All races	1975-1979	98.0	6,676	6,809,090
85+ years	Female	All races	1980-1984	156.5	13,437	8,586,018
85+ years	Female	All races	1985-1989	236.8	23,934	10,106,143
85+ years	Female	All races	1990-1994	320.2	38,366	11,982,171
85+ years	Female	All races	1995-1999	430.0	60,384	14,043,812
85+ years	Female	All races	2000-2004	539.7	83,377	15,449,996
85+ years	Female	All races	2005-2009	584.3	100,540	17,208,283

85+ years	Female	All races	2010-2016	614.4	170,526	27,753,248
85+ years	Female	White	1975-1979	102.0	6,418	6,293,594
85+ years	Female	White	1980-1984	163.0	12,914	7,922,356
85+ years	Female	White	1985-1989	247.2	22,968	9,291,545
85+ years	Female	White	1990-1994	334.6	36,695	10,967,631
85+ years	Female	White	1995-1999	449.9	57,468	12,774,135
85+ years	Female	White	2000-2004	567.4	79,395	13,991,796
85+ years	Female	White	2005-2009	615.4	95,310	15,488,683
85+ years	Female	White	2010-2016	655.5	160,745	24,520,782
85+ years	Female	Black	1975-1979	47.8	221	462,350
85+ years	Female	Black	1980-1984	73.3	433	590,569
85+ years	Female	Black	1985-1989	111.2	797	716,578
85+ years	Female	Black	1990-1994	157.5	1,388	881,195
85+ years	Female	Black	1995-1999	222.9	2,391	1,072,479
85+ years	Female	Black	2000-2004	269.0	3,111	1,156,536
85+ years	Female	Black	2005-2009	310.2	3,968	1,279,291
85+ years	Female	Black	2010-2016	318.4	7,071	2,220,847
85+ years	Female	Other	1975-1979	69.6	37	53,146
85+ years	Female	Other	1980-1984	123.1	90	73,093
85+ years	Female	Other	1985-1989	172.4	169	98,020
85+ years	Female	Other	1990-1994	212.2	283	133,345
85+ years	Female	Other	1995-1999	266.2	525	197,198
85+ years	Female	Other	2000-2004	288.7	871	301,664
85+ years	Female	Other	2005-2009	286.6	1,262	440,309
85+ years	Female	Other	2010-2016	267.9	2,710	1,011,619
