# Supplementary material for: Deaths from COPD in patients with cancer: a population-based study
Source: Aging (Albany NY). 2021 Apr 27;13(9):12641–59. doi: 10.18632/aging.202939 (PMC8148461; doi:10.18632/aging.202939)
Supplement: Supplementary Table 4 [file aging-13-202939-s005.pdf]

Supplementary Table 4. COPD mortality in all patients with cancer by age at diagnosis, sex, race and calendar year of diagnosis

Age at diagnosis	Race	Sex	Calendar year of diagnosis	No. of patients with cancer	Person years of follow-up in total	No. of deaths from COPD	Mortality rates (per 100,000 person-years)
40-44 years	Black	Female	1975-1979	644	9,379	4	42.7
40-44 years	Black	Female	1980-1984	752	11,249	3	26.7
40-44 years	Black	Female	1985-1989	1,017	15,026	6	39.9
40-44 years	Black	Female	1990-1994	2,244	29,476	5	17.0
40-44 years	Black	Female	1995-1999	1,854	21,457	4	18.6
40-44 years	Black	Female	2000-2004	5,520	52,729	8	15.2
40-44 years	Black	Female	2005-2009	6,129	43,323	6	13.8
40-44 years	Black	Female	2010-2016	8,288	23,865	2	8.4
40-44 years	Black	Male	1975-1979	402	2,583	2	77.4
40-44 years	Black	Male	1980-1984	489	3,082	3	97.3
40-44 years	Black	Male	1985-1989	613	4,115	5	121.5
40-44 years	Black	Male	1990-1994	1,446	9,941	6	60.4
40-44 years	Black	Male	1995-1999	1,096	8,374	1	11.9
40-44 years	Black	Male	2000-2004	3,099	22,415	5	22.3
40-44 years	Black	Male	2005-2009	3,123	18,865	7	37.1
40-44 years	Black	Male	2010-2016	3,878	10,177	1	9.8
40-44 years	Other	Female	1975-1979	424	9,436	4	42.4
40-44 years	Other	Female	1980-1984	511	9,429	2	21.2
40-44 years	Other	Female	1985-1989	736	14,184	3	21.2
40-44 years	Other	Female	1990-1994	1,948	31,926	3	9.4
40-44 years	Other	Female	1995-1999	1,865	26,009	3	11.5
40-44 years	Other	Female	2000-2004	4,076	44,838	1	2.2
40-44 years	Other	Female	2005-2009	5,047	38,826	0	0.0
40-44 years	Other	Female	2010-2016	8,428	24,141	1	4.1
40-44 years	Other	Male	1975-1979	173	2,109	0	0.0
40-44 years	Other	Male	1980-1984	228	2,678	0	0.0
40-44 years	Other	Male	1985-1989	270	2,912	0	0.0
40-44 years	Other	Male	1990-1994	795	7,489	1	13.4
40-44 years	Other	Male	1995-1999	705	6,343	2	31.5
40-44 years	Other	Male	2000-2004	1,450	11,599	1	8.6
40-44 years	Other	Male	2005-2009	1,741	10,716	0	0.0
40-44 years	Other	Male	2010-2016	2,695	6,598	3	45.5
40-44 years	White	Female	1975-1979	5,640	119,412	120	100.5
40-44 years	White	Female	1980-1984	6,319	124,547	87	69.9
40-44 years	White	Female	1985-1989	8,963	176,180	76	43.1
40-44 years	White	Female	1990-1994	16,076	277,613	64	23.1

40-44 years	White	Female	1995-1999	13,520	196,376	36	18.3
40-44 years	White	Female	2000-2004	38,734	444,041	60	13.5
40-44 years	White	Female	2005-2009	39,718	314,428	27	8.6
40-44 years	White	Female	2010-2016	52,453	158,361	15	9.5
40-44 years	White	Male	1975-1979	2,843	42,173	48	113.8
40-44 years	White	Male	1980-1984	3,436	51,274	38	74.1
40-44 years	White	Male	1985-1989	4,978	64,531	22	34.1
40-44 years	White	Male	1990-1994	9,373	109,930	41	37.3
40-44 years	White	Male	1995-1999	6,807	79,377	25	31.5
40-44 years	White	Male	2000-2004	19,844	192,606	43	22.3
40-44 years	White	Male	2005-2009	19,978	140,496	11	7.8
40-44 years	White	Male	2010-2016	24,558	69,233	5	7.2
45-49 years	Black	Female	1975-1979	962	12,127	7	57.7
45-49 years	Black	Female	1980-1984	961	12,228	9	73.6
45-49 years	Black	Female	1985-1989	1,137	14,462	8	55.3
45-49 years	Black	Female	1990-1994	2,552	31,500	21	66.7
45-49 years	Black	Female	1995-1999	2,427	27,113	13	47.9
45-49 years	Black	Female	2000-2004	7,592	68,237	23	33.7
45-49 years	Black	Female	2005-2009	8,602	57,695	21	36.4
45-49 years	Black	Female	2010-2016	12,249	34,904	2	5.7
45-49 years	Black	Male	1975-1979	768	4,182	9	215.2
45-49 years	Black	Male	1980-1984	811	4,379	4	91.4
45-49 years	Black	Male	1985-1989	962	5,792	11	189.9
45-49 years	Black	Male	1990-1994	1,947	13,414	13	96.9
45-49 years	Black	Male	1995-1999	1,817	14,298	11	76.9
45-49 years	Black	Male	2000-2004	5,718	41,116	34	82.7
45-49 years	Black	Male	2005-2009	6,148	35,253	13	36.9
45-49 years	Black	Male	2010-2016	7,922	21,060	3	14.2
45-49 years	Other	Female	1975-1979	569	11,552	7	60.6
45-49 years	Other	Female	1980-1984	584	11,564	5	43.2
45-49 years	Other	Female	1985-1989	828	14,723	4	27.2
45-49 years	Other	Female	1990-1994	2,211	35,592	8	22.5
45-49 years	Other	Female	1995-1999	2,572	35,511	4	11.3
45-49 years	Other	Female	2000-2004	5,585	60,320	4	6.6
45-49 years	Other	Female	2005-2009	6,913	51,761	6	11.6
45-49 years	Other	Female	2010-2016	11,227	31,626	1	3.2
45-49 years	Other	Male	1975-1979	277	2,998	0	0.0
45-49 years	Other	Male	1980-1984	307	3,530	1	28.3
45-49 years	Other	Male	1985-1989	374	3,601	1	27.8
45-49 years	Other	Male	1990-1994	1,012	8,964	3	33.5
45-49 years	Other	Male	1995-1999	1,036	8,594	1	11.6
45-49 years	Other	Male	2000-2004	2,323	17,366	6	34.6
45-49 years	Other	Male	2005-2009	2,748	15,919	3	18.8

45-49 years	Other	Male	2010-2016	4,089	9,692	2	20.6
45-49 years	White	Female	1975-1979	9,272	172,571	207	120.0
45-49 years	White	Female	1980-1984	8,530	155,373	167	107.5
45-49 years	White	Female	1985-1989	10,707	194,759	130	66.7
45-49 years	White	Female	1990-1994	20,765	343,102	146	42.6
45-49 years	White	Female	1995-1999	18,242	258,833	72	27.8
45-49 years	White	Female	2000-2004	53,043	596,145	157	26.3
45-49 years	White	Female	2005-2009	60,021	463,381	94	20.3
45-49 years	White	Female	2010-2016	77,766	233,273	43	18.4
45-49 years	White	Male	1975-1979	5,502	60,984	118	193.5
45-49 years	White	Male	1980-1984	5,229	61,527	81	131.6
45-49 years	White	Male	1985-1989	6,315	74,755	73	97.7
45-49 years	White	Male	1990-1994	12,363	142,697	98	68.7
45-49 years	White	Male	1995-1999	10,311	114,304	53	46.4
45-49 years	White	Male	2000-2004	32,516	296,588	124	41.8
45-49 years	White	Male	2005-2009	36,149	240,736	70	29.1
45-49 years	White	Male	2010-2016	43,698	120,642	31	25.7
50-54 years	Black	Female	1975-1979	1,202	12,395	17	137.2
50-54 years	Black	Female	1980-1984	1,267	12,702	11	86.6
50-54 years	Black	Female	1985-1989	1,360	15,120	13	86.0
50-54 years	Black	Female	1990-1994	2,673	28,704	25	87.1
50-54 years	Black	Female	1995-1999	2,513	26,286	15	57.1
50-54 years	Black	Female	2000-2004	8,848	76,585	31	40.5
50-54 years	Black	Female	2005-2009	10,856	67,809	38	56.0
50-54 years	Black	Female	2010-2016	16,971	44,882	21	46.8
50-54 years	Black	Male	1975-1979	1,304	6,234	17	272.7
50-54 years	Black	Male	1980-1984	1,412	7,103	13	183.0
50-54 years	Black	Male	1985-1989	1,438	8,490	12	141.3
50-54 years	Black	Male	1990-1994	2,870	22,124	23	104.0
50-54 years	Black	Male	1995-1999	2,726	23,938	26	108.6
50-54 years	Black	Male	2000-2004	9,617	72,493	53	73.1
50-54 years	Black	Male	2005-2009	11,133	63,267	47	74.3
50-54 years	Black	Male	2010-2016	15,629	39,073	24	61.4
50-54 years	Other	Female	1975-1979	695	12,662	11	86.9
50-54 years	Other	Female	1980-1984	736	11,682	4	34.2
50-54 years	Other	Female	1985-1989	933	15,785	10	63.4
50-54 years	Other	Female	1990-1994	2,197	31,665	8	25.3
50-54 years	Other	Female	1995-1999	2,530	33,140	6	18.1
50-54 years	Other	Female	2000-2004	6,089	64,049	6	9.4
50-54 years	Other	Female	2005-2009	7,909	57,622	3	5.2
50-54 years	Other	Female	2010-2016	13,329	36,428	2	5.5
50-54 years	Other	Male	1975-1979	468	3,738	7	187.3
50-54 years	Other	Male	1980-1984	507	3,990	3	75.2

50-54 years	Other	Male	1985-1989	584	4,908	1	20.4
50-54 years	Other	Male	1990-1994	1,289	11,624	7	60.2
50-54 years	Other	Male	1995-1999	1,426	12,617	7	55.5
50-54 years	Other	Male	2000-2004	3,438	26,256	9	34.3
50-54 years	Other	Male	2005-2009	4,580	26,122	9	34.5
50-54 years	Other	Male	2010-2016	6,941	16,459	3	18.2
50-54 years	White	Female	1975-1979	13,633	225,565	367	162.7
50-54 years	White	Female	1980-1984	12,141	189,094	268	141.7
50-54 years	White	Female	1985-1989	12,419	202,496	245	121.0
50-54 years	White	Female	1990-1994	22,243	333,751	274	82.1
50-54 years	White	Female	1995-1999	21,466	289,532	158	54.6
50-54 years	White	Female	2000-2004	64,169	692,277	309	44.6
50-54 years	White	Female	2005-2009	72,257	535,616	191	35.7
50-54 years	White	Female	2010-2016	106,375	305,779	96	31.4
50-54 years	White	Male	1975-1979	10,021	91,104	212	232.7
50-54 years	White	Male	1980-1984	9,650	95,929	206	214.7
50-54 years	White	Male	1985-1989	9,381	99,081	164	165.5
50-54 years	White	Male	1990-1994	17,384	196,240	202	102.9
50-54 years	White	Male	1995-1999	16,352	182,416	140	76.7
50-54 years	White	Male	2000-2004	54,024	499,380	290	58.1
50-54 years	White	Male	2005-2009	61,189	399,900	222	55.5
50-54 years	White	Male	2010-2016	83,822	225,430	94	41.7
55-59 years	Black	Female	1975-1979	1,355	11,328	12	105.9
55-59 years	Black	Female	1980-1984	1,662	14,065	28	199.1
55-59 years	Black	Female	1985-1989	1,697	15,105	16	105.9
55-59 years	Black	Female	1990-1994	2,902	27,293	36	131.9
55-59 years	Black	Female	1995-1999	2,621	24,196	27	111.6
55-59 years	Black	Female	2000-2004	8,407	66,232	49	74.0
55-59 years	Black	Female	2005-2009	11,298	68,271	52	76.2
55-59 years	Black	Female	2010-2016	19,494	48,055	39	81.2
55-59 years	Black	Male	1975-1979	1,871	8,842	28	316.7
55-59 years	Black	Male	1980-1984	2,234	11,300	34	300.9
55-59 years	Black	Male	1985-1989	2,074	11,921	28	234.9
55-59 years	Black	Male	1990-1994	4,056	33,650	55	163.4
55-59 years	Black	Male	1995-1999	3,469	30,935	40	129.3
55-59 years	Black	Male	2000-2004	12,178	94,485	91	96.3
55-59 years	Black	Male	2005-2009	15,345	88,154	83	94.2
55-59 years	Black	Male	2010-2016	23,259	55,955	43	76.8
55-59 years	Other	Female	1975-1979	673	9,956	5	50.2
55-59 years	Other	Female	1980-1984	875	12,184	11	90.3
55-59 years	Other	Female	1985-1989	1,186	16,119	8	49.6
55-59 years	Other	Female	1990-1994	2,357	31,472	19	60.4
55-59 years	Other	Female	1995-1999	2,276	27,950	13	46.5

55-59 years	Other	Female	2000-2004	5,710	56,462	7	12.4
55-59 years	Other	Female	2005-2009	8,011	56,039	4	7.1
55-59 years	Other	Female	2010-2016	13,993	36,688	6	16.4
55-59 years	Other	Male	1975-1979	574	4,241	11	259.3
55-59 years	Other	Male	1980-1984	836	7,205	10	138.8
55-59 years	Other	Male	1985-1989	874	7,151	13	181.8
55-59 years	Other	Male	1990-1994	1,887	15,826	24	151.6
55-59 years	Other	Male	1995-1999	1,822	15,765	17	107.8
55-59 years	Other	Male	2000-2004	4,559	35,413	14	39.5
55-59 years	Other	Male	2005-2009	6,066	35,875	14	39.0
55-59 years	Other	Male	2010-2016	9,830	22,524	11	48.8
55-59 years	White	Female	1975-1979	16,772	228,980	452	197.4
55-59 years	White	Female	1980-1984	16,691	217,236	461	212.2
55-59 years	White	Female	1985-1989	16,545	226,664	462	203.8
55-59 years	White	Female	1990-1994	24,429	324,843	529	162.8
55-59 years	White	Female	1995-1999	21,597	265,126	290	109.4
55-59 years	White	Female	2000-2004	69,643	703,078	719	102.3
55-59 years	White	Female	2005-2009	79,393	566,563	375	66.2
55-59 years	White	Female	2010-2016	122,438	331,787	234	70.5
55-59 years	White	Male	1975-1979	15,085	116,720	386	330.7
55-59 years	White	Male	1980-1984	16,466	138,086	399	289.0
55-59 years	White	Male	1985-1989	16,030	148,537	421	283.4
55-59 years	White	Male	1990-1994	25,388	278,377	444	159.5
55-59 years	White	Male	1995-1999	22,195	239,809	314	130.9
55-59 years	White	Male	2000-2004	74,823	687,145	682	99.3
55-59 years	White	Male	2005-2009	89,299	589,121	507	86.1
55-59 years	White	Male	2010-2016	124,772	320,924	284	88.5
60-64 years	Black	Female	1975-1979	1,329	9,580	13	135.7
60-64 years	Black	Female	1980-1984	1,722	12,340	28	226.9
60-64 years	Black	Female	1985-1989	2,168	17,346	40	230.6
60-64 years	Black	Female	1990-1994	3,294	26,550	47	177.0
60-64 years	Black	Female	1995-1999	2,698	21,751	36	165.5
60-64 years	Black	Female	2000-2004	8,286	59,375	74	124.6
60-64 years	Black	Female	2005-2009	10,623	60,101	68	113.1
60-64 years	Black	Female	2010-2016	20,209	47,804	51	106.7
60-64 years	Black	Male	1975-1979	1,950	9,369	33	352.2
60-64 years	Black	Male	1980-1984	2,780	12,873	52	403.9
60-64 years	Black	Male	1985-1989	2,833	15,701	51	324.8
60-64 years	Black	Male	1990-1994	5,461	43,705	109	249.4
60-64 years	Black	Male	1995-1999	4,389	37,865	85	224.5
60-64 years	Black	Male	2000-2004	13,015	98,939	162	163.7
60-64 years	Black	Male	2005-2009	15,795	92,384	124	134.2
60-64 years	Black	Male	2010-2016	25,940	61,583	85	138.0

60-64 years	Other	Female	1975-1979	584	6,747	11	163.0
60-64 years	Other	Female	1980-1984	873	9,924	14	141.1
60-64 years	Other	Female	1985-1989	1,293	16,568	15	90.5
60-64 years	Other	Female	1990-1994	2,564	29,994	20	66.7
60-64 years	Other	Female	1995-1999	2,417	26,040	20	76.8
60-64 years	Other	Female	2000-2004	4,992	46,053	17	36.9
60-64 years	Other	Female	2005-2009	6,922	45,353	15	33.1
60-64 years	Other	Female	2010-2016	14,552	36,890	13	35.2
60-64 years	Other	Male	1975-1979	742	4,794	12	250.3
60-64 years	Other	Male	1980-1984	983	6,699	10	149.3
60-64 years	Other	Male	1985-1989	1,334	9,988	21	210.3
60-64 years	Other	Male	1990-1994	2,717	23,074	43	186.4
60-64 years	Other	Male	1995-1999	2,419	20,257	28	138.2
60-64 years	Other	Male	2000-2004	5,392	40,435	31	76.7
60-64 years	Other	Male	2005-2009	6,902	39,907	27	67.7
60-64 years	Other	Male	2010-2016	12,407	28,642	15	52.4
60-64 years	White	Female	1975-1979	17,888	197,489	465	235.5
60-64 years	White	Female	1980-1984	20,112	220,238	564	256.1
60-64 years	White	Female	1985-1989	21,947	260,207	681	261.7
60-64 years	White	Female	1990-1994	30,464	351,380	862	245.3
60-64 years	White	Female	1995-1999	22,312	243,256	466	191.6
60-64 years	White	Female	2000-2004	67,663	618,890	1,097	177.3
60-64 years	White	Female	2005-2009	82,094	548,145	770	140.5
60-64 years	White	Female	2010-2016	134,201	357,633	438	122.5
60-64 years	White	Male	1975-1979	19,617	127,486	508	398.5
60-64 years	White	Male	1980-1984	22,337	164,134	695	423.4
60-64 years	White	Male	1985-1989	24,484	205,647	738	358.9
60-64 years	White	Male	1990-1994	39,057	400,293	1,059	264.6
60-64 years	White	Male	1995-1999	28,034	286,877	597	208.1
60-64 years	White	Male	2000-2004	87,445	759,427	1,408	185.4
60-64 years	White	Male	2005-2009	105,177	676,831	1,034	152.8
60-64 years	White	Male	2010-2016	156,420	407,648	600	147.2
65-69 years	Black	Female	1975-1979	1,316	7,835	7	89.3
65-69 years	Black	Female	1980-1984	1,679	9,750	19	194.9
65-69 years	Black	Female	1985-1989	2,199	14,056	33	234.8
65-69 years	Black	Female	1990-1994	3,823	25,765	72	279.4
65-69 years	Black	Female	1995-1999	2,881	20,342	62	304.8
65-69 years	Black	Female	2000-2004	8,443	53,553	93	173.7
65-69 years	Black	Female	2005-2009	9,923	52,538	86	163.7
65-69 years	Black	Female	2010-2016	18,289	41,214	62	150.4
65-69 years	Black	Male	1975-1979	2,108	8,516	32	375.8
65-69 years	Black	Male	1980-1984	2,687	11,305	48	424.6
65-69 years	Black	Male	1985-1989	3,336	17,109	68	397.5

65-69 years	Black	Male	1990-1994	6,629	48,147	155	321.9
65-69 years	Black	Male	1995-1999	4,575	35,795	115	321.3
65-69 years	Black	Male	2000-2004	13,371	96,329	241	250.2
65-69 years	Black	Male	2005-2009	14,600	82,574	172	208.3
65-69 years	Black	Male	2010-2016	23,438	55,024	123	223.5
65-69 years	Other	Female	1975-1979	482	4,069	5	122.9
65-69 years	Other	Female	1980-1984	826	7,571	3	39.6
65-69 years	Other	Female	1985-1989	1,235	12,951	16	123.5
65-69 years	Other	Female	1990-1994	2,911	29,734	30	100.9
65-69 years	Other	Female	1995-1999	2,608	25,281	16	63.3
65-69 years	Other	Female	2000-2004	5,319	42,729	29	67.9
65-69 years	Other	Female	2005-2009	6,508	40,061	14	34.9
65-69 years	Other	Female	2010-2016	13,422	32,337	15	46.4
65-69 years	Other	Male	1975-1979	877	4,678	15	320.6
65-69 years	Other	Male	1980-1984	1,114	6,594	25	379.1
65-69 years	Other	Male	1985-1989	1,616	10,943	27	246.7
65-69 years	Other	Male	1990-1994	3,978	33,688	92	273.1
65-69 years	Other	Male	1995-1999	3,261	27,318	57	208.7
65-69 years	Other	Male	2000-2004	6,503	49,803	70	140.6
65-69 years	Other	Male	2005-2009	7,927	45,339	63	139.0
65-69 years	Other	Male	2010-2016	13,829	32,031	31	96.8
65-69 years	White	Female	1975-1979	17,383	150,419	364	242.0
65-69 years	White	Female	1980-1984	21,018	185,134	581	313.8
65-69 years	White	Female	1985-1989	25,071	248,643	871	350.3
65-69 years	White	Female	1990-1994	37,633	371,673	1,317	354.3
65-69 years	White	Female	1995-1999	26,268	246,372	842	341.8
65-69 years	White	Female	2000-2004	70,663	580,819	1,833	315.6
65-69 years	White	Female	2005-2009	78,115	484,144	1,243	256.7
65-69 years	White	Female	2010-2016	138,669	350,334	784	223.8
65-69 years	White	Male	1975-1979	21,278	113,872	608	533.9
65-69 years	White	Male	1980-1984	25,872	156,448	869	555.5
65-69 years	White	Male	1985-1989	30,056	219,145	1,019	465.0
65-69 years	White	Male	1990-1994	54,531	497,015	2,027	407.8
65-69 years	White	Male	1995-1999	36,113	330,383	1,072	324.5
65-69 years	White	Male	2000-2004	98,336	800,166	2,342	292.7
65-69 years	White	Male	2005-2009	107,286	663,923	1,685	253.8
65-69 years	White	Male	2010-2016	171,425	433,808	944	217.6
70-74 years	Black	Female	1975-1979	1,051	4,938	3	60.8
70-74 years	Black	Female	1980-1984	1,526	7,204	14	194.3
70-74 years	Black	Female	1985-1989	1,879	10,500	20	190.5
70-74 years	Black	Female	1990-1994	3,476	19,832	59	297.5
70-74 years	Black	Female	1995-1999	2,899	16,938	53	312.9
70-74 years	Black	Female	2000-2004	7,843	42,368	115	271.4

70-74 years	Black	Female	2005-2009	8,757	40,960	106	258.8
70-74 years	Black	Female	2010-2016	14,056	30,085	89	295.8
70-74 years	Black	Male	1975-1979	1,694	6,174	25	404.9
70-74 years	Black	Male	1980-1984	2,337	9,056	44	485.9
70-74 years	Black	Male	1985-1989	2,647	11,272	44	390.3
70-74 years	Black	Male	1990-1994	5,946	35,983	147	408.5
70-74 years	Black	Male	1995-1999	4,218	27,264	118	432.8
70-74 years	Black	Male	2000-2004	10,806	65,673	213	324.3
70-74 years	Black	Male	2005-2009	10,884	55,243	188	340.3
70-74 years	Black	Male	2010-2016	15,450	34,664	133	383.7
70-74 years	Other	Female	1975-1979	480	2,496	4	160.2
70-74 years	Other	Female	1980-1984	575	4,285	9	210.0
70-74 years	Other	Female	1985-1989	1,069	9,118	11	120.6
70-74 years	Other	Female	1990-1994	2,619	22,152	37	167.0
70-74 years	Other	Female	1995-1999	2,609	20,761	33	159.0
70-74 years	Other	Female	2000-2004	5,206	35,660	43	120.6
70-74 years	Other	Female	2005-2009	6,240	33,161	34	102.5
70-74 years	Other	Female	2010-2016	10,611	24,179	24	99.3
70-74 years	Other	Male	1975-1979	903	4,040	13	321.8
70-74 years	Other	Male	1980-1984	1,181	5,898	23	389.9
70-74 years	Other	Male	1985-1989	1,569	9,198	36	391.4
70-74 years	Other	Male	1990-1994	4,268	30,710	123	400.5
70-74 years	Other	Male	1995-1999	3,762	26,405	79	299.2
70-74 years	Other	Male	2000-2004	6,736	45,671	121	264.9
70-74 years	Other	Male	2005-2009	7,470	38,638	78	201.9
70-74 years	Other	Male	2010-2016	11,362	25,135	49	194.9
70-74 years	White	Female	1975-1979	15,801	105,115	265	252.1
70-74 years	White	Female	1980-1984	19,571	134,821	463	343.4
70-74 years	White	Female	1985-1989	23,649	181,151	729	402.4
70-74 years	White	Female	1990-1994	39,574	316,187	1,453	459.5
70-74 years	White	Female	1995-1999	29,701	237,064	1,150	485.1
70-74 years	White	Female	2000-2004	74,782	519,486	2,270	437.0
70-74 years	White	Female	2005-2009	72,405	397,972	1,509	379.2
70-74 years	White	Female	2010-2016	114,949	269,983	1,020	377.8
70-74 years	White	Male	1975-1979	20,427	89,992	572	635.6
70-74 years	White	Male	1980-1984	24,534	120,216	840	698.7
70-74 years	White	Male	1985-1989	30,191	178,265	1,096	614.8
70-74 years	White	Male	1990-1994	56,730	428,661	2,238	522.1
70-74 years	White	Male	1995-1999	37,716	287,316	1,442	501.9
70-74 years	White	Male	2000-2004	101,442	706,343	3,253	460.5
70-74 years	White	Male	2005-2009	94,791	531,785	2,106	396.0
70-74 years	White	Male	2010-2016	136,498	325,561	1,210	371.7
75-79 years	Black	Female	1975-1979	830	3,443	4	116.2

75-79 years	Black	Female	1980-1984	1,139	4,510	10	221.7
75-79 years	Black	Female	1985-1989	1,486	6,383	17	266.3
75-79 years	Black	Female	1990-1994	2,789	12,670	33	260.5
75-79 years	Black	Female	1995-1999	2,491	11,874	43	362.1
75-79 years	Black	Female	2000-2004	7,126	31,790	115	361.8
75-79 years	Black	Female	2005-2009	7,318	28,380	99	348.8
75-79 years	Black	Female	2010-2016	11,090	21,631	74	342.1
75-79 years	Black	Male	1975-1979	1,145	3,419	15	438.8
75-79 years	Black	Male	1980-1984	1,581	4,801	29	604.0
75-79 years	Black	Male	1985-1989	1,906	6,345	28	441.3
75-79 years	Black	Male	1990-1994	3,985	18,744	103	549.5
75-79 years	Black	Male	1995-1999	3,002	14,991	81	540.3
75-79 years	Black	Male	2000-2004	7,947	38,277	222	580.0
75-79 years	Black	Male	2005-2009	7,233	30,419	129	424.1
75-79 years	Black	Male	2010-2016	9,632	19,485	106	544.0
75-79 years	Other	Female	1975-1979	379	1,708	4	234.1
75-79 years	Other	Female	1980-1984	518	2,512	7	278.7
75-79 years	Other	Female	1985-1989	783	4,432	8	180.5
75-79 years	Other	Female	1990-1994	2,040	12,517	22	175.8
75-79 years	Other	Female	1995-1999	2,158	13,421	25	186.3
75-79 years	Other	Female	2000-2004	4,482	26,066	70	268.5
75-79 years	Other	Female	2005-2009	5,488	25,357	55	216.9
75-79 years	Other	Female	2010-2016	8,710	17,642	24	136.0
75-79 years	Other	Male	1975-1979	717	2,668	14	524.8
75-79 years	Other	Male	1980-1984	934	3,666	14	381.9
75-79 years	Other	Male	1985-1989	1,405	6,483	35	539.8
75-79 years	Other	Male	1990-1994	3,319	18,363	89	484.7
75-79 years	Other	Male	1995-1999	2,983	17,211	77	447.4
75-79 years	Other	Male	2000-2004	6,073	33,363	149	446.6
75-79 years	Other	Male	2005-2009	6,247	28,003	121	432.1
75-79 years	Other	Male	2010-2016	9,161	17,981	64	355.9
75-79 years	White	Female	1975-1979	13,668	68,290	128	187.4
75-79 years	White	Female	1980-1984	16,928	90,123	301	334.0
75-79 years	White	Female	1985-1989	20,537	120,942	464	383.7
75-79 years	White	Female	1990-1994	34,556	208,834	1,104	528.6
75-79 years	White	Female	1995-1999	27,925	174,491	1,026	588.0
75-79 years	White	Female	2000-2004	77,284	446,711	2,592	580.2
75-79 years	White	Female	2005-2009	71,578	340,981	1,847	541.7
75-79 years	White	Female	2010-2016	94,449	208,010	1,126	541.3
75-79 years	White	Male	1975-1979	16,255	57,135	423	740.4
75-79 years	White	Male	1980-1984	19,585	76,187	646	847.9
75-79 years	White	Male	1985-1989	24,071	110,743	929	838.9
75-79 years	White	Male	1990-1994	43,903	246,200	1,738	705.9

75-79 years	White	Male	1995-1999	31,281	184,010	1,253	680.9
75-79 years	White	Male	2000-2004	87,989	492,547	3,203	650.3
75-79 years	White	Male	2005-2009	82,508	393,446	2,267	576.2
75-79 years	White	Male	2010-2016	101,770	221,780	1,267	571.3
80-84 years	Black	Female	1975-1979	524	1,522	0	0.0
80-84 years	Black	Female	1980-1984	751	2,075	3	144.6
80-84 years	Black	Female	1985-1989	968	3,021	8	264.8
80-84 years	Black	Female	1990-1994	1,901	6,223	22	353.5
80-84 years	Black	Female	1995-1999	1,619	5,638	20	354.7
80-84 years	Black	Female	2000-2004	5,042	17,147	67	390.7
80-84 years	Black	Female	2005-2009	5,501	17,406	85	488.3
80-84 years	Black	Female	2010-2016	7,692	12,896	77	597.1
80-84 years	Black	Male	1975-1979	571	1,523	9	591.1
80-84 years	Black	Male	1980-1984	854	2,141	13	607.3
80-84 years	Black	Male	1985-1989	1,090	3,125	23	736.0
80-84 years	Black	Male	1990-1994	2,119	7,341	52	708.4
80-84 years	Black	Male	1995-1999	1,450	5,003	43	859.6
80-84 years	Black	Male	2000-2004	4,477	15,048	118	784.2
80-84 years	Black	Male	2005-2009	4,303	13,722	98	714.2
80-84 years	Black	Male	2010-2016	5,209	8,704	77	884.7
80-84 years	Other	Female	1975-1979	269	862	1	116.0
80-84 years	Other	Female	1980-1984	363	1,318	2	151.7
80-84 years	Other	Female	1985-1989	491	2,153	4	185.8
80-84 years	Other	Female	1990-1994	1,219	5,024	11	219.0
80-84 years	Other	Female	1995-1999	1,364	5,966	21	352.0
80-84 years	Other	Female	2000-2004	3,193	13,563	42	309.7
80-84 years	Other	Female	2005-2009	4,339	16,136	42	260.3
80-84 years	Other	Female	2010-2016	7,075	12,102	41	338.8
80-84 years	Other	Male	1975-1979	363	1,047	4	382.2
80-84 years	Other	Male	1980-1984	584	1,824	13	712.9
80-84 years	Other	Male	1985-1989	852	2,906	16	550.6
80-84 years	Other	Male	1990-1994	2,165	8,633	48	556.0
80-84 years	Other	Male	1995-1999	1,734	7,039	40	568.2
80-84 years	Other	Male	2000-2004	3,831	15,679	103	656.9
80-84 years	Other	Male	2005-2009	4,366	15,458	104	672.8
80-84 years	Other	Male	2010-2016	6,416	10,449	68	650.8
80-84 years	White	Female	1975-1979	10,670	38,492	52	135.1
80-84 years	White	Female	1980-1984	12,594	47,204	126	266.9
80-84 years	White	Female	1985-1989	15,045	63,597	258	405.7
80-84 years	White	Female	1990-1994	25,631	112,260	631	562.1
80-84 years	White	Female	1995-1999	20,376	91,184	593	650.3
80-84 years	White	Female	2000-2004	60,372	261,343	1,705	652.4
80-84 years	White	Female	2005-2009	62,668	242,854	1,681	692.2

80-84 years	White	Female	2010-2016	76,835	152,362	1,071	702.9
80-84 years	White	Male	1975-1979	11,224	30,433	245	805.0
80-84 years	White	Male	1980-1984	12,442	37,042	368	993.5
80-84 years	White	Male	1985-1989	14,642	49,708	457	919.4
80-84 years	White	Male	1990-1994	26,019	102,770	951	925.4
80-84 years	White	Male	1995-1999	18,971	74,412	708	951.5
80-84 years	White	Male	2000-2004	56,783	228,414	2,076	908.9
80-84 years	White	Male	2005-2009	58,050	214,149	1,824	851.7
80-84 years	White	Male	2010-2016	72,665	136,151	1,187	871.8
85+ years	Black	Female	1975-1979	394	897	1	111.5
85+ years	Black	Female	1980-1984	565	1,069	1	93.5
85+ years	Black	Female	1985-1989	781	1,678	4	238.3
85+ years	Black	Female	1990-1994	1,615	3,619	15	414.5
85+ years	Black	Female	1995-1999	1,392	2,957	13	439.7
85+ years	Black	Female	2000-2004	4,617	9,743	52	533.7
85+ years	Black	Female	2005-2009	5,089	10,848	54	497.8
85+ years	Black	Female	2010-2016	7,300	9,158	66	720.7
85+ years	Black	Male	1975-1979	358	646	2	309.8
85+ years	Black	Male	1980-1984	501	962	9	935.2
85+ years	Black	Male	1985-1989	639	1,278	19	1,486.8
85+ years	Black	Male	1990-1994	1,246	2,830	24	848.0
85+ years	Black	Male	1995-1999	950	1,977	32	1,618.3
85+ years	Black	Male	2000-2004	2,794	6,174	61	988.1
85+ years	Black	Male	2005-2009	2,693	5,606	59	1,052.4
85+ years	Black	Male	2010-2016	3,527	4,211	57	1,353.6
85+ years	Other	Female	1975-1979	198	375	0	0.0
85+ years	Other	Female	1980-1984	324	624	2	320.7
85+ years	Other	Female	1985-1989	482	1,049	2	190.6
85+ years	Other	Female	1990-1994	1,023	2,252	11	488.6
85+ years	Other	Female	1995-1999	1,057	2,792	13	465.5
85+ years	Other	Female	2000-2004	2,360	5,893	24	407.2
85+ years	Other	Female	2005-2009	3,580	8,515	41	481.5
85+ years	Other	Female	2010-2016	6,949	8,792	56	636.9
85+ years	Other	Male	1975-1979	311	478	8	1,673.6
85+ years	Other	Male	1980-1984	368	681	8	1,175.2
85+ years	Other	Male	1985-1989	553	1,214	10	823.9
85+ years	Other	Male	1990-1994	1,329	3,550	32	901.3
85+ years	Other	Male	1995-1999	1,233	3,132	45	1,436.7
85+ years	Other	Male	2000-2004	2,418	6,076	73	1,201.5
85+ years	Other	Male	2005-2009	3,063	6,963	94	1,350.0
85+ years	Other	Male	2010-2016	5,428	6,553	76	1,159.7
85+ years	White	Female	1975-1979	8,807	20,212	30	148.4
85+ years	White	Female	1980-1984	11,757	27,559	70	254.0

85+ years	White	Female	1985-1989	13,527	34,210	106	309.9
85+ years	White	Female	1990-1994	22,522	59,073	338	572.2
85+ years	White	Female	1995-1999	18,495	48,492	330	680.5
85+ years	White	Female	2000-2004	51,942	135,350	1,088	803.8
85+ years	White	Female	2005-2009	58,586	147,327	1,285	872.2
85+ years	White	Female	2010-2016	82,046	120,496	1,189	986.8
85+ years	White	Male	1975-1979	7,500	14,599	114	780.9
85+ years	White	Male	1980-1984	9,025	18,354	190	1,035.2
85+ years	White	Male	1985-1989	9,732	22,228	226	1,016.7
85+ years	White	Male	1990-1994	15,997	39,574	437	1,104.3
85+ years	White	Male	1995-1999	11,833	28,249	360	1,274.4
85+ years	White	Male	2000-2004	35,669	89,294	1,092	1,222.9
85+ years	White	Male	2005-2009	40,776	98,335	1,214	1,234.6
85+ years	White	Male	2010-2016	60,198	84,303	975	1,156.5
